# cis-Locked Ru(II)-DMSO Precursors for the Microwave-Assisted Synthesis of Bis-Heteroleptic Polypyridyl Compounds

**DOI:** 10.1021/acs.inorgchem.1c00240

**Published:** 2021-04-28

**Authors:** Alessio Vidal, Rudy Calligaro, Gilles Gasser, Roger Alberto, Gabriele Balducci, Enzo Alessio

**Affiliations:** † Department of Chemical and Pharmaceutical Sciences, University of Trieste, Via L. Giorgieri 1, 34127 Trieste, Italy; ‡ Chimie ParisTech, PSL University, CNRS, Institute of Chemistry for Life and Health Sciences, Laboratory for Inorganic Chemistry, 75005 Paris, France; § Department of Chemistry, University of Zurich, Winterthurerstrasse 190, CH-8057 Zurich, Switzerland

## Abstract

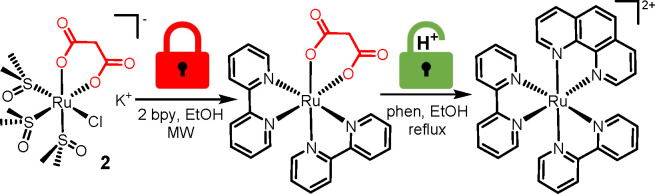

We describe a synthetic strategy for the preparation of bis-heteroleptic polypyridyl Ru(II) complexes of the type [Ru(L1)_2_(L2)]^2+^ (L1 and L2 = diimine ligands) from well-defined Ru(II) precursors. For this purpose, a series of six neutral, anionic, and cationic *cis*-locked Ru(II)-DMSO complexes (**2**–**7**) of the general formula [Y] *fac*-[RuX(DMSO–S)_3_(O–O)]^*n*^ (where O–O is a symmetrical chelating anion: oxalate (ox), malonate (mal), acetylacetonate (acac); X = DMSO–O or Cl^–^; *n* = −1/0/+1 depending on the nature and charge of X and O–O; when present, Y = K^+^ or PF_6_^–^) were efficiently prepared from the well-known *cis*-[RuCl_2_(DMSO)_4_] (**1**). When treated with diimine chelating ligands (L1 = bpy, phen, dpphen), the compounds **2**–**7** afforded the target [Ru(L1)_2_(O–O)]^0/+^ complex together with the undesired (and unexpected) [Ru(L1)_3_]^2+^ species. Nevertheless, we found that the formation of [Ru(L1)_3_]^2+^can be minimized by carefully adjusting the reaction conditions: in particular, high selectivity toward [Ru(L1)_2_(O–O)]^0/+^ and almost complete conversion of the precursor was obtained within minutes, also on a 100–200 mg scale, when the reactions were performed in absolute ethanol at 150 °C in a microwave reactor. Depending on the nature of L1 and concentration, with the oxalate and malonate precursors, the neutral product [Ru(L1)_2_(O–O)] can precipitate spontaneously from the final mixture, in pure form and acceptable-to-good yields. When spontaneous precipitation of the disubstituted product does not occur, purification from [Ru(L1)_3_]^2+^ can be rather easily accomplished by column chromatography or solvent extraction. By comparison, under the same conditions, compound **1** is much less selective, thus demonstrating that locking the geometry of the precursor through the introduction of O–O in the coordination sphere of Ru is a valid strategic approach. By virtue of its proton-sensitive nature, facile and quantitative replacement of O–O in [Ru(L1)_2_(O–O)]^0/+^ by L2, selectively affording [Ru(L1)_2_(L2)]^2+^, was accomplished in refluxing ethanol in the presence of a slight excess of trifluoroacetic acid or HPF_6_.

## Abstract 2

*cis*-Locked Ru(II)-DMSO complexes bearing a symmetrical chelating anion, such as [K] *fac*-[RuCl(DMSO−S)_3_(η^2^-mal)] (**2**), are suitable precursors for the two-step selective preparation of bis-heteroleptic polypyridyl compounds of the type [Ru(L1)_2_(L2)]^2+^ (L1 and L2 = diimine ligands).

## Introduction

**Figure 1:**
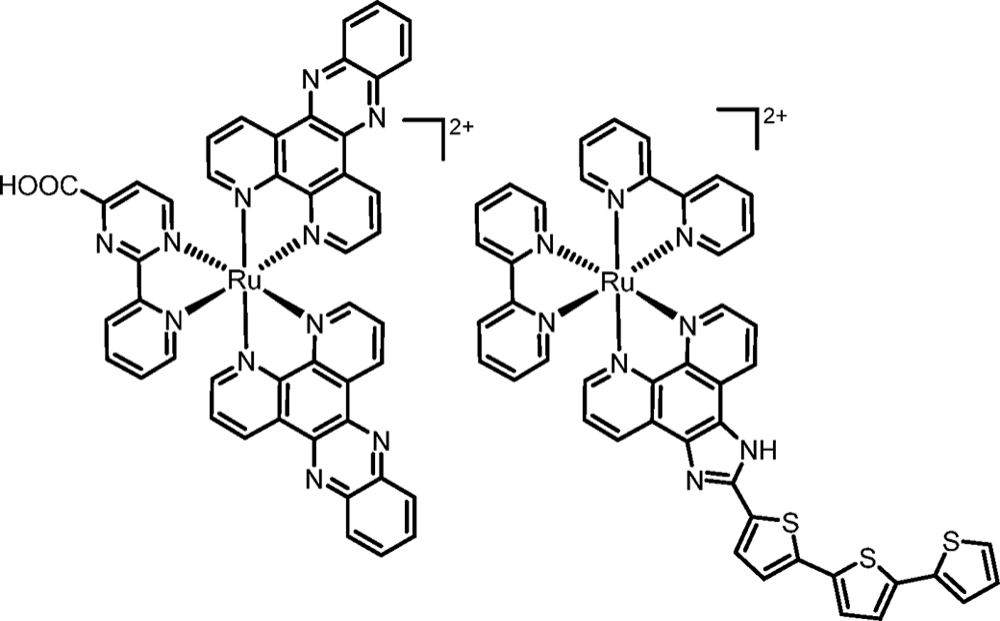
Two representative examples of bis-heteroleptic [Ru(L1)_2_(L2)]^2+^ compounds that are relevant in medicinal inorganic chemistry: [Ru(dppz)_2_(cppH)]^2+^ (left, cppH = 2-(2′-pyridyl)pyrimidine-4-carboxylic acid) and TLD1433 (right).

Ruthenium(II) polypyridyl complexes are well-known to the inorganic chemistry community, mainly because of their interesting photophysical and photochemical properties (e.g., strong absorption and emission bands in the visible-to-NIR region, long-lived triplet excited states).^1^ Most importantly, by varying the nature and the number of the polypyridyl ligands around the Ru(II) center such properties can be accurately fine-tuned.^2^ The numerous applications of Ru(II) polypyridyl complexes span widely different fields, from light-into-electrical energy conversion (e.g., in dye-sensitized solar cells, DSSCs)^3,4^ and photoredox catalysis^5,6^ to biomedicine.^2,7−12^ In this latter field, by virtue of their multiple excited state relaxation pathways, Ru(II) polypyridyl complexes can be exploited either for sensing and imaging applications or as photosensitizers in photodynamic therapy (PDT) and photochemotherapy (PCT).^2,7−22^ Remarkably, a ruthenium polypyridyl photosensitizer (named TLD1433, Figure 1) is currently being studied in the clinic for photodynamic therapy.^23,24^ In addition, Ru(II) polypyridyl complexes are being actively investigated for their light-independent anticancer properties.^25−32^

Notwithstanding the widespread interest in this class of compounds,^33^ we found that the synthetic aspects leading to their preparation are still amenable to being improved. The synthetic procedures leading to Ru(II) polypyridyl complexes bearing three equal or different diimine chelating ligands (L) were thoroughly reviewed by Spiccia and co-workers in 2004.^34^ Quite obviously, upon going from homoleptic [Ru(L1)_3_]^2+^ to tris-heterlopetic [Ru(L1)(L2)(L3)]^2+^ compounds, the synthetic procedures become more challenging. Optical resolution of the chiral-at-metal Λ and Δ enantiomers adds an additional level of complexity.^35^ The reader interested in this specific topic is referred to the relatively recent review by Meggers and co-workers.^36^

Bis-heteroleptic [Ru(L1)_2_(L2)]^2+^ compounds (Figure 1) are typically obtained from the neutral intermediate *cis*-[RuCl_2_(L1)_2_],^37^ which is predominantly synthesized by the procedure originally described by Meyer and co-workers in 1978.^38^ It requires treatment of hydrated RuCl_3_ with L1 in hot DMF in the presence of an excess of LiCl (to impede chloride replacement and formation of cationic byproducts) and, in some preparations, of a reducing agent.^39^ In some cases the dichloride intermediate is not isolated but further reacted *in situ* with L2.^35^ Even though apparently straightforward, this procedure—that works well for simple diimines such as bpy and for large-scale preparations—has a number of drawbacks, e.g., poor control of the stoichiometry due to the uncertain nature of the ruthenium precursor,^40^ formation of carbonyl byproducts due to the noninnocent and thermally unstable solvent,^41^ requirement of very high concentrations that are unpractical for small-scale preparations, and difficulty in the removal of excess LiCl. Finally, the unknown redox mechanism is perhaps scarcely relevant in practical terms but is nevertheless unsatisfactory from the scientific point of view. These shortcomings led to the investigation of alternative procedures. Stepwise synthetic methods that might allow also for the preparation of the more demanding tris-heteroleptic compounds are particularly interesting.^4^ Synthetic procedures starting from a Ru(II) precursor would seem particularly logical. There are essentially three such routes, two that utilize an organometallic precursor,^42^ either the oligomeric carbonyl [RuCl_2_(CO)_2_]_*n*_ (that involves either a photoassisted or a chemically assisted decarbonylation route)^43−47^ or the dinuclear half-sandwich compound [Ru(η^6^-C_6_H_6_)Cl_2_]_2_ (that however requires the photoassisted release of the aromatic ring after the insertion of the first diimine ligand),^48−50^ and one that exploits the coordination complex *cis*-[RuCl_2_(DMSO)_4_] (**1**). Compound **1** has a number of advantages over the others, besides its low cost and commercial availability: (*i*) unlike [RuCl_2_(CO)_2_]_*n*_ and RuCl_3_·3H_2_O, it is a well-defined species,^40^ allowing for precise control of the stoichiometry of the reactants; (*ii*) it is obtained in a single step preparation from the universal ruthenium precursor RuCl_3_·3H_2_O in high yield and excellent purity;^51^ (*iii*) it is nontoxic, perfectly stable in air, and soluble in a wide range of solvents, from water to chloroform;^52^ (*iv*) it does not require the use of a photoreactor. The route from complex **1** is treated in more detail below.

### *cis*-[RuCl_2_(DMSO)_4_] As Precursor for the Preparation of Ru(II) Polypyridyl Complexes

When *cis*-[RuCl_2_(DMSO)_4_] is treated with neutral ligands, the DMSO ligands are expected to be preferentially, and in a stepwise manner, replaced to yield neutral products.^40,52^ In particular, consistent with the reactivity observed by us with neutral monodentate pyridyl and azole ligands,^40,53^ a neutral diimine ligand should quite easily replace the DMSO–O (i.e., the weakest ligand) and an adjacent DMSO–S, whereas the two remaining DMSO–S’s are expected to require relatively harsher conditions to be substituted, thus allowing—in principle—for the stepwise introduction of different diimines.^54^ However, contrasting reports are present in the literature, and the outcome of the reactions seems to depend on several factors. In fact, not only are the nature of the solvent (and therefore the temperature) and the L1/Ru ratio relevant but also the nature of L1. For example, Burke and Keyes reported that treatment of **1** with 1 equiv of dppz (dppz = dipyrido[3,2-a:2′,3′-c]phenazine) in refluxing EtOH affords *cis*,*cis*-[RuCl_2_(dppz)(DMSO–S)_2_] selectively and with high yield,^55^ whereas for Grätzel and co-workers the reaction for obtaining the corresponding complex with 4,4′-dimethyl-bpy must be run at lower temperatures (i.e., refluxing CHCl_3_) for being selective.^54^ In fact, according to these authors, protic solvents (e.g., EtOH) or high boiling aprotic solvents afforded mixtures of mono- and bis-substituted complexes. On the other hand, Bowman and co-workers reported that reaction of **1** with 1 equiv of 1,10-phenanthroline (phen) in refluxing CHCl_3_ gave only a low conversion, whereas in refluxing toluene, *cis*,*cis*-[RuCl_2_(DMSO–S)_2_(phen)] precipitated with excellent yield.^56^ Similarly, according to Toyama and co-workers *cis*,*cis*-[RuCl_2_(DMSO–S)_2_(bpy)] (bpy = 2,2′-bipyridine) is selectively obtained by refluxing **1** with 1 equiv of bpy in a 9:1 EtOH/DMSO mixture.^57^ Finally, we and others found that the substitution of two DMSO ligands in **1** with chelating diimines can be accompanied by isomerization of the remaining ligands, affording stereoisomers.^57,58^

According to literature reports, treatment of **1** with 2 equiv of L1 in refluxing organic solvents (ranging from chloroform to ethylene glycol) leads usually to the replacement of all four DMSO ligands affording [RuCl_2_(L1)_2_] species. The reaction can be accompanied by isomerization of the two chlorides from *cis* to *trans* geometry.^59,60^ However, there are examples in which the neutral diimines replace three DMSO ligands and a chloride, yielding cationic complexes of the type *cis*-[RuCl(L1)_2_(DMSO–S)]Cl.^61,62^

Stimulated by these often contradicting literature reports, we investigated the reactions of **1** with selected diimine chelating ligands (e.g., L1 = bpy, phen, and dppz) under different conditions and found that it typically yields mixtures of substitutional isomers, often as stereoisomers (see below). Of note, the formation of stereoisomers with *trans* geometry of the remaining monodentate ligands (e.g., *trans*,*cis*-[RuCl_2_(L1)(DMSO–S)_2_], *cis*,*trans*-[RuCl_2_(L1)(DMSO–S)_2_], and *trans*-[RuCl_2_(L1)_2_]) and/or substitutional isomers (e.g., [RuCl(L1)_2_(DMSO–S)]Cl) creates practical problems (e.g., isolation and characterization of mixtures of intermediates), but it is not necessarily detrimental for the attainment of the final [Ru(L1)_2_(L2)]^2+^ product (even though it cannot be excluded that the required *trans*-to-*cis* stereochemical rearrangement might induce significant kinetic barriers in the process).

Both Meggers and co-workers and, more recently, Burke and Keyes proposed synthetic strategies for the preparation of bis- and tris-heteroleptic polypyridyl Ru complexes from **1** that avoid the formation of stereoisomers:^55,63^ either in the first or second step of the synthetic procedure an auxiliary bidentate ligand of switchable binding strength (aux) is introduced in the coordination sphere of Ru(II), obtaining intermediates of the type [Ru(L1)_2_(aux)]^*n*+^ or [Ru(L1)(L2)(aux)]^*n*+^ (aux = oxalate, *n* = 0; or aux = chiral salicyloxazoline, *n* = 1). The coordination of the last diimine ligand (L3) is preceded by the acid-assisted removal of the aux ligand.

**Figure 2:**
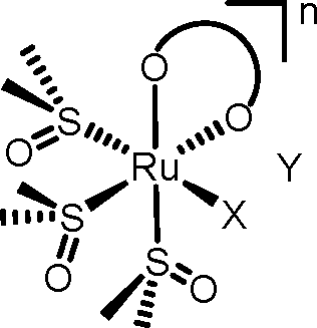
Schematic representation of a *cis*-locked Ru(II)-DMSO precursor with a chelating oxygenated anion (O–O) developed in this work. X = DMSO or Cl. The charge (*n* = −1/0/+1) depends on the nature and charge of X and O–O. When present, Y = K^+^ or PF_6_^–^.

**Scheme 1:**
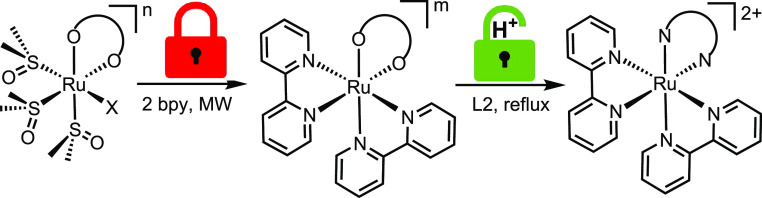
Schematic Representation of the Two-Step Preparation of Bis-Heteroleptic Products [Ru(L1)_2_(L2)]^2+^ (Exemplified for L1 = bpy) from a *cis*-Locked Ru(II)-DMSO Precursor The charge of the starting compound (*n*) and of the intermediate (*m*) depends on the nature and charge of X and O–O. Counterion omitted.

Building on this approach and on our own experience on Ru-DMSO compounds,^40,52^ we thought of developing new *cis*-locked Ru(II)-DMSO complexes by replacing either the two chlorides, or a chloride and a DMSO, in **1** with an inert chelating anion (O–O, Figure 2), and investigating them as precursors for the two-step preparation of bis-heteroleptic products [Ru(L1)_2_(L2)]^2+^, as detailed in Scheme 1.

Such precursors are expected to have, in principle, a number of advantages over compound **1**: (*i*) The presence of a chelate that locks the geometry avoids the formation of stereoisomers. Replacement of the four relatively labile DMSO/Cl ligands by chelating diimines (L1) will thus be stereocontrolled. (*ii*) Since the chelating O–O ligand is supposed to be more strongly bound to ruthenium compared to the chlorides in **1**, the possibility of obtaining substitutional byproducts (e.g., [Ru(L1)_3_]^2+^) should be lower compared to **1**. (*iii*) In the second step, the proton-sensitive nature of O–O is expected to allow its replacement under relatively mild acidic conditions. Thus, contrary to what is often found in the literature, this step might be performed at relatively low temperatures. This feature might become particularly relevant if the auxiliary ligand has a chiral center and is enantiomerically pure: in this case, an excess of one diastereoisomer of the bischelate adduct is obtained, and low temperatures should limit potential racemization in the last step. (*iv*) In principle, provided that the monodentate ligands can be pairwise replaced in two steps with sufficient selectivity, this approach might be suitable also for the preparation of tris-heteroleptic polypyridyl products.

In order to avoid the formation of stereoisomers, the symmetrical chelating anions (O–O) oxalate (ox), malonate (mal), and acetylacetonate (acac) were selected. Even though ox might form stronger chelate rings compared to mal and acac, it has some drawbacks: it is proton-NMR silent and has additional binding modes available (besides the η^2^-ox) in which it bridges two metal ions using all four oxygen atoms (η^4^,μ-ox) or, occasionally, only three of them (η^3^,μ-ox). The dimethyl-malonate ligand (dmmal), which would be excellent for the purpose of NMR detection, was discarded because it preferentially forms the dinuclear species [*fac*-Ru(DMSO–S)_3_(OH_2_)(μ-dmmal)]_2_ when reacted with **1**.^64^

## Results and Discussion

### Reactivity of **1** with Model Diimine Ligands

We reinvestigated some of the reactions of *cis*-[RuCl_2_(DMSO)_4_] (**1**) with the model diimine ligand phen. As detailed in the Supporting Information, we found that (*i*) contrary to what is reported in the literature, the reaction of **1** with 1 equiv of phen in refluxing chloroform for 1 h (i.e., the conditions of refs (34) and (54)), besides being largely incomplete, affords a ca. 1:1 mixture of the two stereoisomers *cis*,*cis*-[RuCl_2_(DMSO–S)_2_(phen)] (**a**) and *trans*,*cis*-[RuCl_2_(DMSO–S)_2_(phen)] (**b**). (*ii*) The same reaction performed in refluxing ethanol for 2 h (i.e., the conditions used in ref (55) for the selective preparation of *cis*,*cis*-[RuCl_2_(DMSO–S)_2_(dppz)]) was complete and afforded a ca. 5:1 mixture of **a** and *cis*,*trans*-[RuCl_2_(DMSO–S)_2_(phen)] (**c**) and a minor amount of the disubstituted cationic product *cis*-[RuCl(DMSO–S)(phen)_2_]Cl (**d**).^65^ (*iii*) Similar results, but with a larger amount of compound **d**, were obtained also when **1** was treated with 2 (rather than one) equiv of phen in refluxing ethanol (up to 8 h). In contrast with literature reports, the expected disubstituted neutral species *cis*-[RuCl_2_(phen)_2_] was not detected among the products. On the positive side, the dead-end trisubstituted species [Ru(phen)_3_]^2+^ was not found among the products either. This finding was somehow surprising because, even though some preparations of [Ru(chel)_3_]^2+^ compounds from **1** are typically performed at a higher temperature, it was nevertheless reported that—for example—treatment of **1** with 3 equiv of dppz in refluxing ethanol produced [Ru(dppz)_3_]^2+^ exclusively.^66^

### Preparation of *cis*-Locked Ru(II)-DMSO Precursors

**Figure 3:**
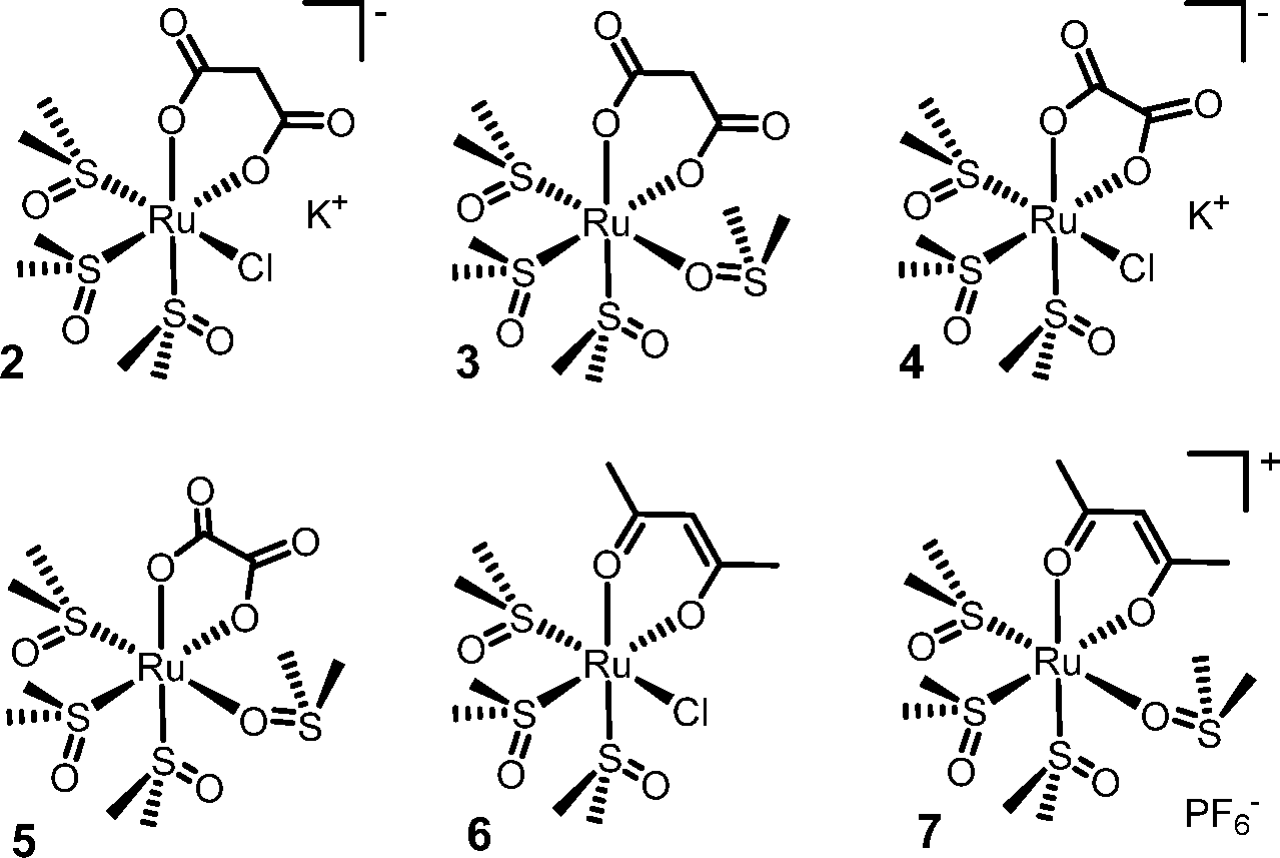
The six *cis*-locked Ru(II)-DMSO precursors [K]*fac*-[RuCl(DMSO–S)_3_(η^2^-mal)] (**2**), *fac*-[Ru(DMSO–O)(DMSO–S)_3_(η^2^-mal)] (**3**), [K] *fac*-[RuCl(DMSO–S)_3_(η^2^-ox)] (**4**), *fac*-[Ru(DMSO–O)(DMSO–S)_3_(η^2^-ox)] (**5**), *fac*-[RuCl(DMSO–S)_3_(η^2^-acac)] (**6**), and *fac*-[Ru(DMSO–O)(DMSO–S)_3_(η^2^-acac)][PF_6_] (**7**).

In the past, we developed, for different reasons, the *cis*-locked dicarboxylate complexes [K]*fac*-[RuCl(DMSO–S)_3_(η^2^-mal)] (**2**), *fac*-[Ru(DMSO–O)(DMSO–S)_3_(η^2^-mal)] (**3**), [K]*fac*-[RuCl(DMSO–S)_3_(η^2^-ox)] (**4**), and *fac*-[Ru(DMSO–O)(DMSO–S)_3_(η^2^-ox)] (**5**; Figure 2).^64^ We report here, besides improved preparations for **4** and **5**, also the synthesis and characterization of two additional complexes of this series with the monoanionic acetylacetonate (acac) ligand: the neutral *fac*-[RuCl(DMSO–S)_3_(η^2^-acac)] (**6**) and the cationic *fac*-[Ru(DMSO–O)(DMSO–S)_3_(η^2^-acac)][PF_6_] (**7**; Figure 3).

**Scheme 2:**
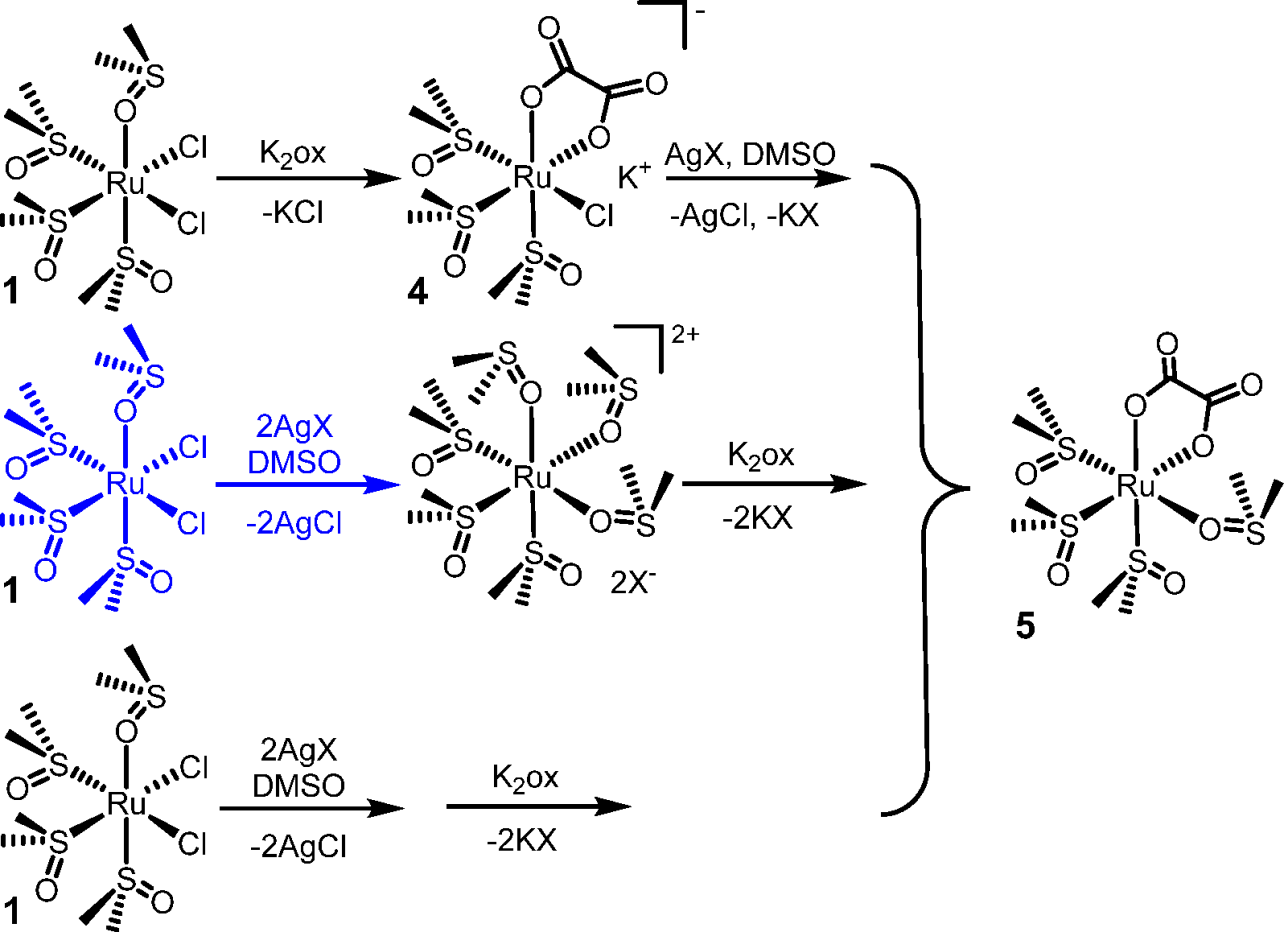
General Procedures for the Preparations of Complexes **3**, **5**, and **7** (X = CF_3_SO_3_, NO_3_, PF_6_) Exemplified in the Case of **5** (O–O = oxalate) The blue part shows the preparation of the chloride-free precursor *fac*-[Ru(DMSO–O)_3_(DMSO–S)_3_][X]_2_.

In summary, both mono- and dianionic O–O chelates react with **1**, replacing the DMSO–O and an adjacent chloride, yielding **2**, **4**, and **6**. We had no evidence of products derived from the spontaneous substitution of both chlorides of **1**, even when the reaction was performed in aqueous DMSO. Chloride-free complexes **3**, **5**, and **7** were prepared by silver-assisted chloride abstraction from the corresponding monochloride intermediates or from the chloride-free precursor *fac*-[Ru(DMSO–O)_3_(DMSO–S)_3_](X)_2_ (which is obtained in one step from **1**, X = CF_3_SO_3_, NO_3_, PF_6_)^40,52^ or, more conveniently, directly from **1** in a one-pot reaction (Scheme 2). The first route requires only 1 equiv of AgX but also the isolation of an intermediate (and thus lower final yields).

**Figure 4:**
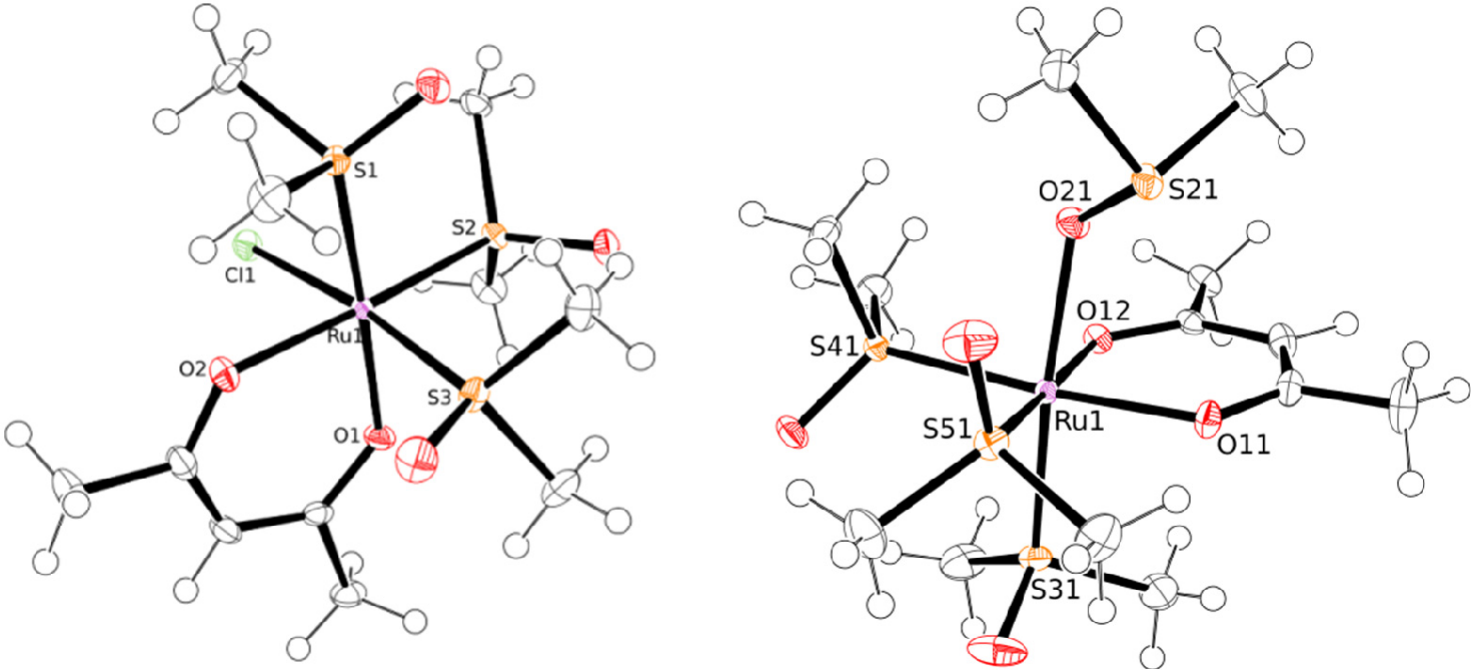
ORTEP representation (50% probability ellipsoids) of complex *fac*-[RuCl(DMSO–S)_3_(η^2^-acac)] (**6**, left) and of the cation of *fac*-[Ru(DMSO–O)(DMSO–S)_3_(η^2^-acac)][PF_6_] (**7**, right).

All compounds **2**–**7** were fully characterized by IR and NMR spectroscopy and ESI MS spectrometry (including isotope distribution). The ^1^H NMR spectra are consistent with the *C*_*s*_ symmetry of the complexes (i.e., the O–O ligand is symmetrically bound *trans* to two DMSO–S ligands). They show a pattern of three singlets (6H each) in the region for S-bonded DMSO, which is typical for the {*fac*-Ru(DMSO-S)_3_} fragment. The DMSO–O in **3**, **5**, and **7** resonates as a singlet at about 2.8 ppm. The diastereotopic protons of the mal ligand in **2** and **3** give two doublets (1H each), whereas the acac ligand in **6** and **7** gives two singlets (1H and 6H, respectively). The X-ray structures of the new compounds **6** and **7** are shown in Figure 4. Geometrical parameters are in line with those previously reported for the dicarboxylate analogues.^64^ At room temperature, all complexes are soluble in water (with the exception of **7**), methanol, and DMSO. The neutral complexes **3** and **6** (but not the ox compound **5**) are soluble also in chloroform, and the cationic PF_6_ complex **7** is soluble in acetone.

Having this homogeneous set of six *cis*-protected complexes in hand, we investigated their reactivities toward the model diimine chelating ligand bpy and, in some cases, phen and 4,7-diphenylphenanthroline (dpphen).

We have previously shown that, in water, the release of the chloride from the anionic complexes *fac*-[RuCl(DMSO–S)_3_(O–O)]^−^ (O–O = ox, mal) is much slower compared to the release of DMSO–O from the corresponding neutral *fac*-[Ru(DMSO–O)(DMSO–S)_3_(O–O)] species, despite the charge difference.^64^ Overall, these findings suggest that the DMSO–O complexes **3**, **5**, and **7** are expected to be more reactive than the corresponding chloride compounds **2**, **4**, and **6**. In addition, when treated with L1, the former complexes do not generate inorganic salts (e.g., KCl) as coproducts; however, the synthetic effort for their preparation from **1** is higher.

### Reactivity of **2**–**7** with bpy

**Scheme 3:**
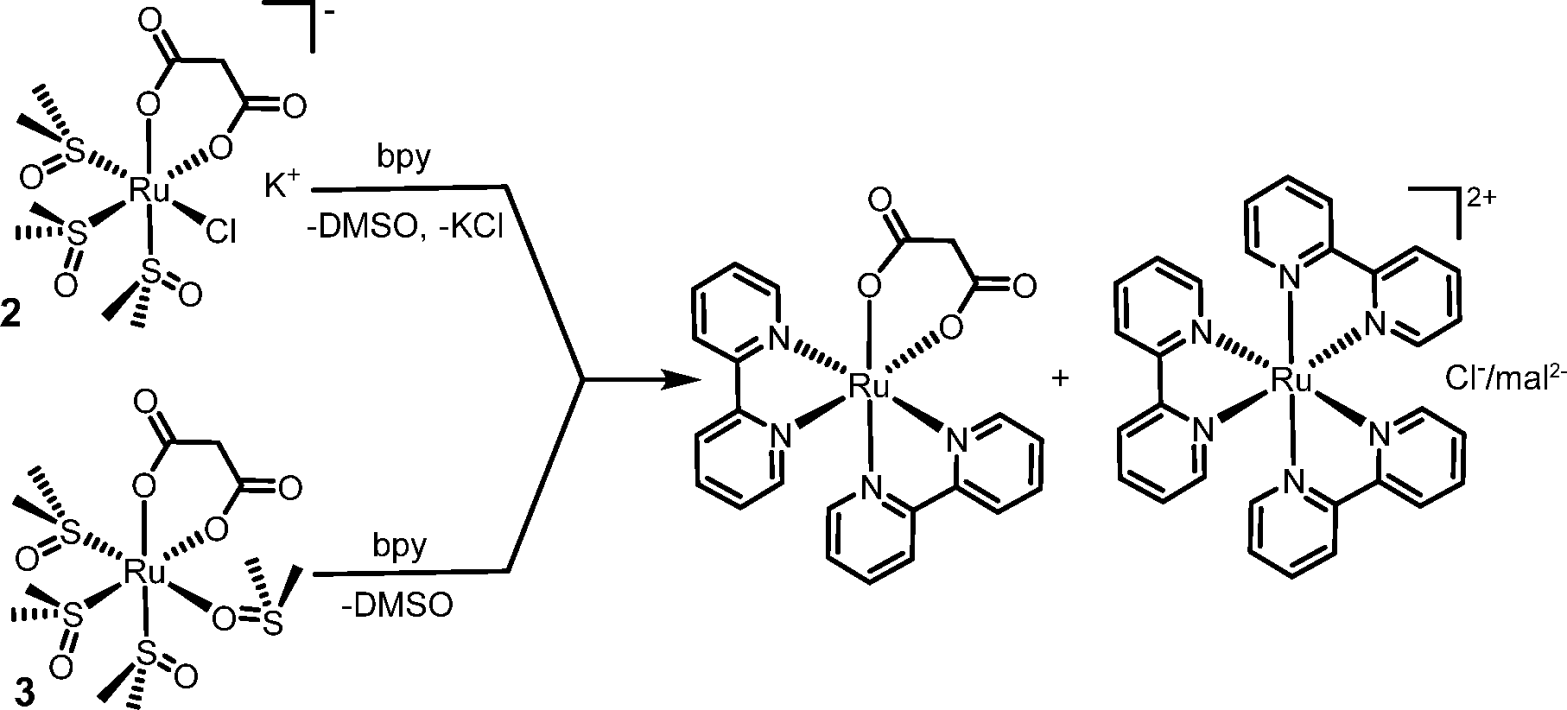
General Reactivity of the *cis*-Locked Dicarboxylate Precursors **2**–**5** towards bpy (Case of Malonate Is Exemplified)

**Scheme 4:**
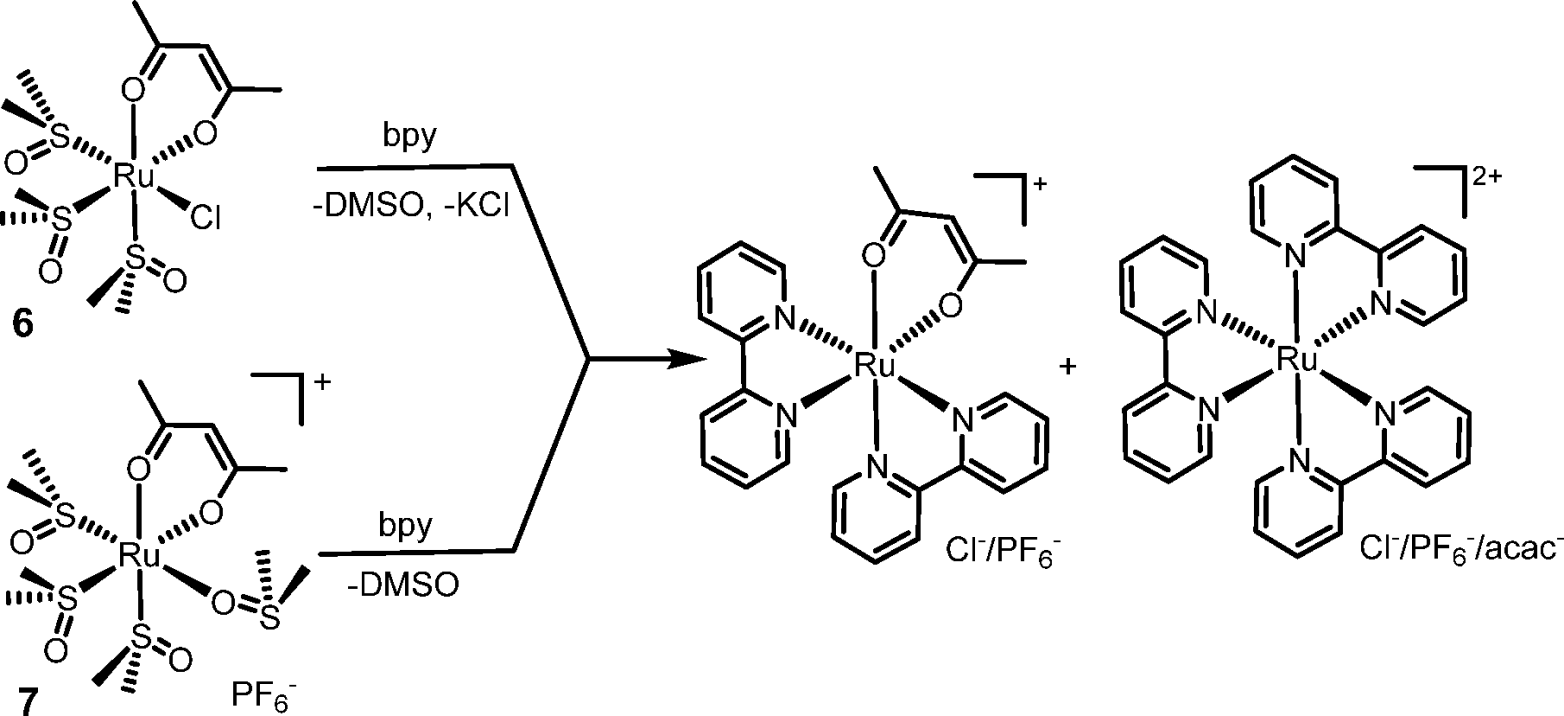
General Reactivity of the *cis*-Locked acac Precursors **6** and **7** towards bpy

The model reaction with bpy was carried out with all six precursors, and a number of parameters, such as solvent, temperature, concentration, reaction time, and bpy/Ru ratio, were systematically investigated. Reactions were carried out in absolute ethanol, which turned out to be the most appropriate solvent—together with methanol—among those screened (that include acetone, chloroform, DMSO, toluene, and acetonitrile). The rather slow reaction rates observed under reflux conditions improved substantially by performing the reactions in a microwave (MW) reactor. NMR and TLC analyses indicated that, at the end of the reaction,^67^ the solution typically contains variable amounts of two products identified as [Ru(bpy)_2_(O–O)]^*n*+^ (*n* = 0, 1 depending on O–O) and the unwanted (and unexpected) [Ru(bpy)_3_]^2+^. We choose ^1^H NMR spectroscopy in DMSO-*d*_6_ as the most appropriate analytical method for a rapid, reliable, and quantitative assessment of the reaction outcome. In fact, since the charge of the products can range from 0 to +2, we found that only DMSO is capable of dissolving the mixture completely, regardless of the nature of L1 and O–O. Other solvents, such as CDCl_3_ or D_2_O, led to the underestimation (or overestimation) of one of the components. The reaction schemes are reported in Scheme 3 for compounds **2**–**5**, and in Scheme 4 for **6** and **7**.

The following reactivity order, based on the amount of residual bpy (after 1h at 120 °C) was found, **4** < **5** < **2** < **3**, thus confirming that chloride-free precursors are more reactive. However, the ox compound **5** yielded mainly [Ru(bpy)_3_]^2+^. The acac complex **6** was less selective, affording—besides [Ru(bpy)_2_(η^2^-acac)]^+^, [Ru(bpy)_3_]^3+^ and unreacted bpy—at least two other minor unidentified Ru-bpy species. In the case of **7**, a dark precipitate was obtained at the end of the reaction that was identified, according to the NMR spectrum, as a mixture of [Ru(bpy)_2_(η^2^-acac)][PF_6_] (**8**) and [Ru(bpy)_3_][PF_6_]_2_.^68^ Compound **8** was isolated in pure form by extracting the mixture with chloroform.^69,70^

The selectivity toward the desired bis-bpy product was found to increase upon increasing the temperature and decreasing the concentration (Supporting Information), even though low concentrations are unpractical for preparative purposes and afford lower conversions. In the case of **2**, an increase of the bpy/Ru ratio from 2 to 4 led to no appreciable improvement in selectivity and conversion. A good compromise between conversion and selectivity was obtained with the malonate precursors **2** and **3** that in 10 min at 150 °C afforded ca. 90% bpy conversion with up to 90% selectivity in [Ru(bpy)_2_(η^2^-mal)] (according to NMR integration). Under similar conditions, the “unlocked” precursor **1** was much less selective, affording a mixture of at least five species, bearing from one to three bpy ligands, identified as *cis*,*cis*-[RuCl_2_(bpy)(DMSO–S)_2_], *cis*,*trans*-[RuCl_2_(bpy)(DMSO–S)_2_], *cis*-[RuCl_2_(bpy)_2_], *cis*-[RuCl(bpy)_2_(DMSO–S)]^+^, and [Ru(bpy)_3_]^2+^, whose relative amounts were found to depend on temperature, concentration, and reaction time (Supporting Information).

**Figure 5:**
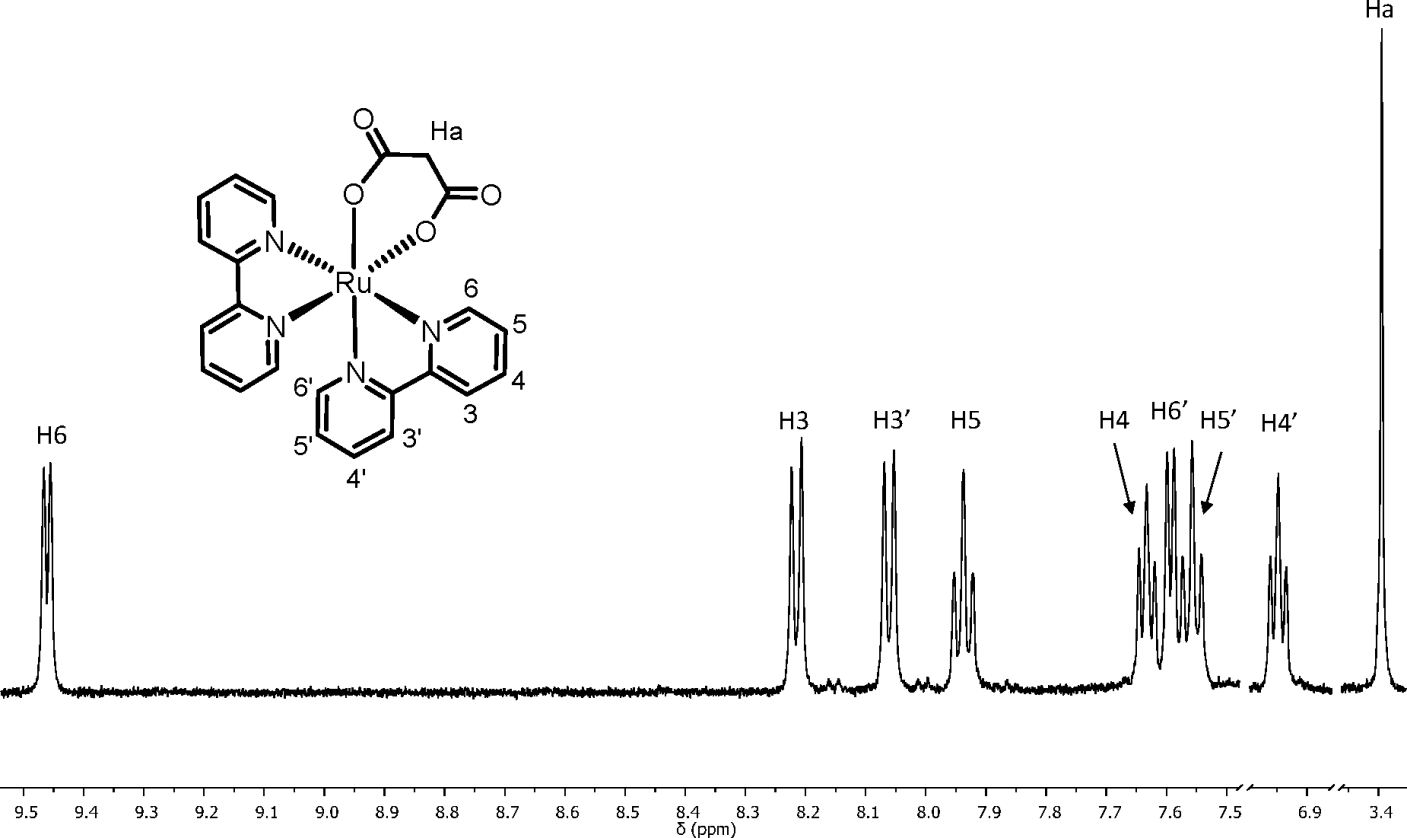
^1^H NMR spectrum (CDCl_3_) of [Ru(bpy)_2_(η^2^-mal)] (**9**). See the inset for labeling scheme.

In general, since the main detected byproduct has a +2 charge, separation of the neutral complexes [Ru(bpy)_2_(O–O)] by chromatography or by extraction/washing with an appropriate solvent is easily feasible. As an example, [Ru(bpy)_2_(η^2^-mal)] (**9**) was obtained in pure form from the reaction mixture by column chromatography on silica gel (see also below). The pure compounds **8** and **9** were fully characterized by ^1^H NMR spectroscopy (Figure 5 and Supporting Information). For both complexes, the two equivalent bpy ligands give eight equally intense resonances, which were assigned through COSY and HSQC spectra and by considering the mutual-shielding effects.^48,71^ The UV–vis spectra of **8** and **9** are very similar (and similar to that of [RuCl_2_(bpy)_2_]) and characterized by two absorption bands in the visible region (at ca. 370 and 530 nm, Supporting Information).

### Reactions of Selected *cis*-Locked Ru(II) Precursors with phen and dpphen on a Larger Scale

**Figure 6:**
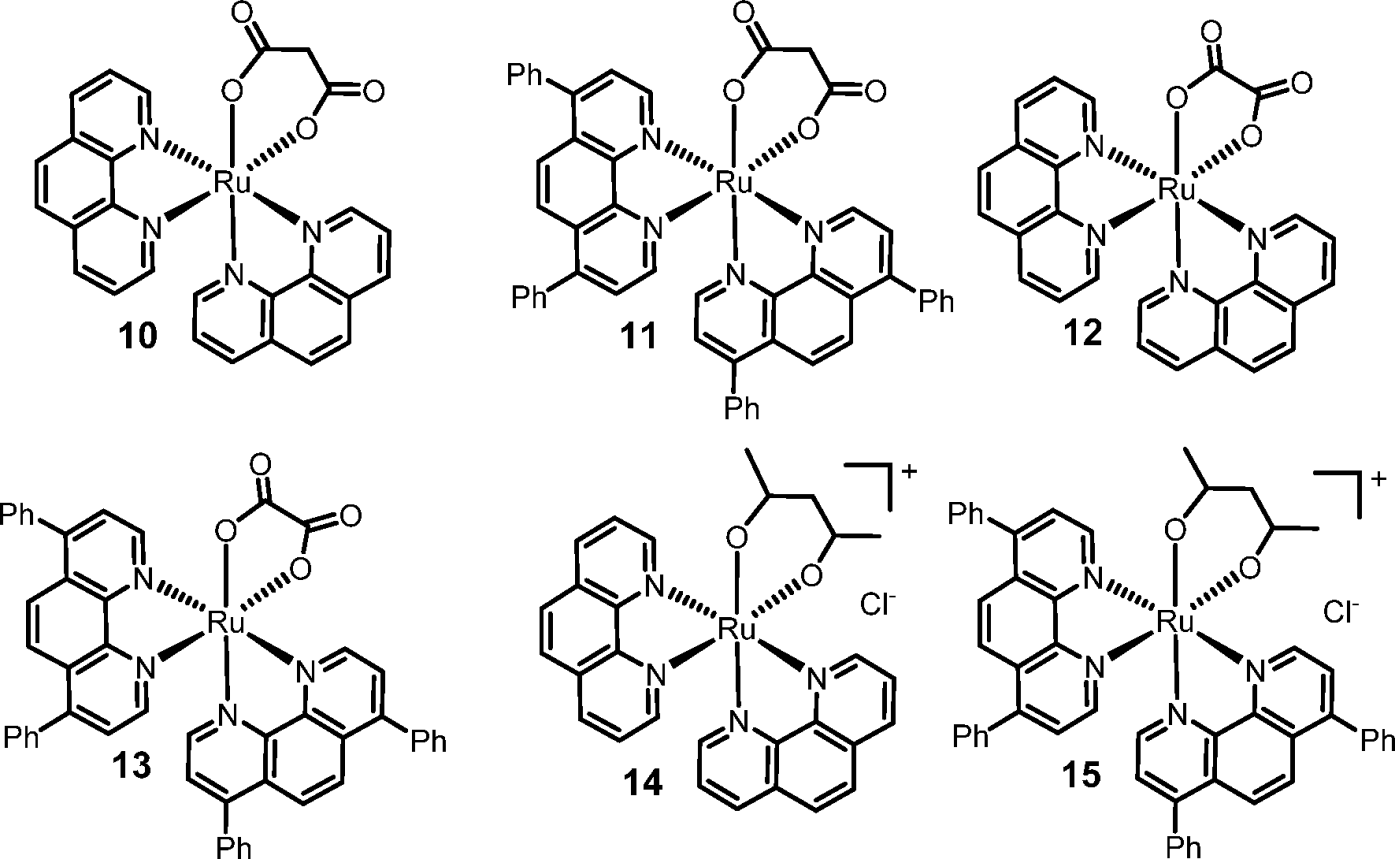
Complexes [Ru(phen)_2_(η^2^-mal)] (**10**), [Ru(dpphen)_2_(η^2^-mal)] (**11**), [Ru(phen)_2_(η^2^-ox)] (**12**), [Ru(dpphen)_2_(η^2^-ox)] (**13**), [Ru(phen)_2_(η^2^-acac)]Cl (**14**), and [Ru(dpphen)_2_(η^2^-acac)]Cl (**15**).

We tested our synthetic approach on a slightly larger preparative scale, with the aim of obtaining 100–200 mg for each of the complexes [Ru(L1)_2_(O–O)] and [Ru(L1)_2_(η^2^-acac)]Cl (L1 = phen and dpphen, O–O = mal and ox), using the chloride compounds **2**, **4**, and **6** as precursors. The reactions were performed at 150 °C in 10 or 30 mL MW vials. The concentration of each Ru precursor was in the 120–200 mM range. We found that, using an appropriately high concentration of the precursor, the neutral products [Ru(L1)_2_(O–O)] precipitated spontaneously from the mother liquor at the end of the reaction; conversely, no precipitation was observed with bpy. Thus, the complexes [Ru(phen)_2_(η^2^-mal)] (**10**), [Ru(dpphen)_2_(η^2^-mal)] (**11**), [Ru(phen)_2_(η^2^-ox)] (**12**), and [Ru(dpphen)_2_(η^2^-ox)] (**13**; Figure 6) were easily recovered by filtration.

According to ^1^H NMR spectroscopy, they were obtained in pure form; coprecipitated KCl was easily removed by recrystallization from chloroform (except for **12**, see the Experimental Section). The less soluble oxalate complexes were obtained in 70–80% yields, whereas those with malonate were in quite lower yields. In the case of **10**, however, we found that an increase of the phen/**2** ratio from 2 to 3 improved the yield significantly (from 36 to 51%). According to TLC and NMR analysis, the mother liquor contained mainly a mixture of [Ru(L1)_2_(O–O)] and [Ru(L1)_3_]^2+^. Column chromatography performed on the mother liquor of the reaction between **2** and phen afforded, as first fraction, a small amount of *cis*-[RuCl_2_(phen)_2_], indicating that at least part of the Cl^–^ released from **2** upon formation of **10** is capable of replacing the malonate. This chloride-rebound mechanism, already encountered by some of us on similar complexes,^72^ confirms the high affinity of Cl^–^ for Ru(II) and—consistent with the ubiquitous formation of [Ru(L1)_3_]^2+^ species—the insufficient strength of the O–O chelate (see below).

In contrast, the acac complexes [Ru(phen)_2_(η^2^-acac)]Cl (**14**) and [Ru(dpphen)_2_(η^2^-acac)]Cl (**15**; Figure 5) did not precipitate spontaneously and were obtained in pure form by column chromatography in moderate yields.^73^

**Figure 7:**
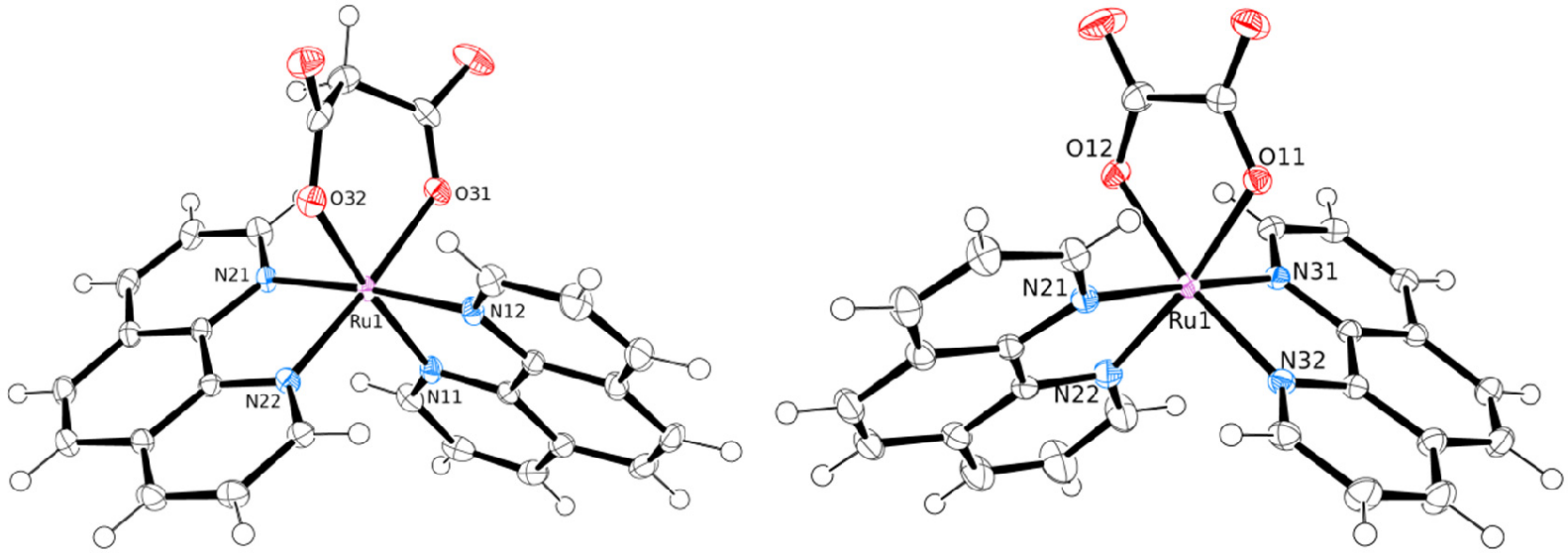
ORTEP representation (50% probability ellipsoids) of complexes [Ru(phen)_2_(η^2^-mal)]·5H_2_O (**10**·5H_2_O, left) and [Ru(phen)_2_(η^2^-ox)]·H_2_O (**12**·H_2_O, right) in their crystal structures. Disordered cocrystallized water molecules have been omitted for clarity.

**Figure 8:**
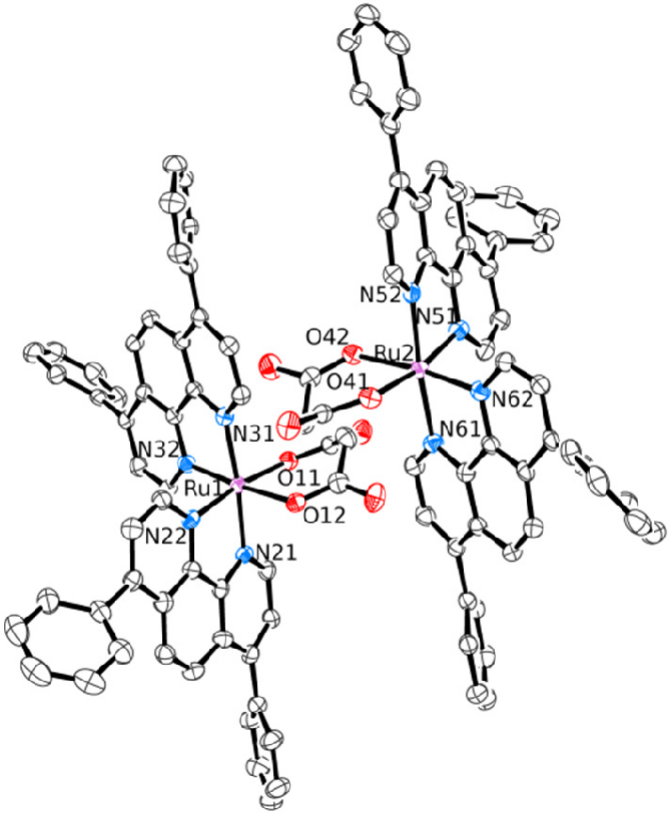
ORTEP representation (50% probability ellipsoids) of the two independent molecules of compound [Ru(dpphen)_2_(η^2^-mal)]·3.75H_2_O (**11**·3.75H_2_O) in the crystal structure. Cocrystallized water molecules and H atoms have been omitted for clarity.

The X-ray structures of the [Ru(phen)_2_(η^2^-mal)] (**10**), [Ru(dpphen)_2_(η^2^-mal)] (**11**), and [Ru(phen)_2_(η^2^-ox)] (**12**) are shown in Figures 7 and 8.

**Figure 9:**
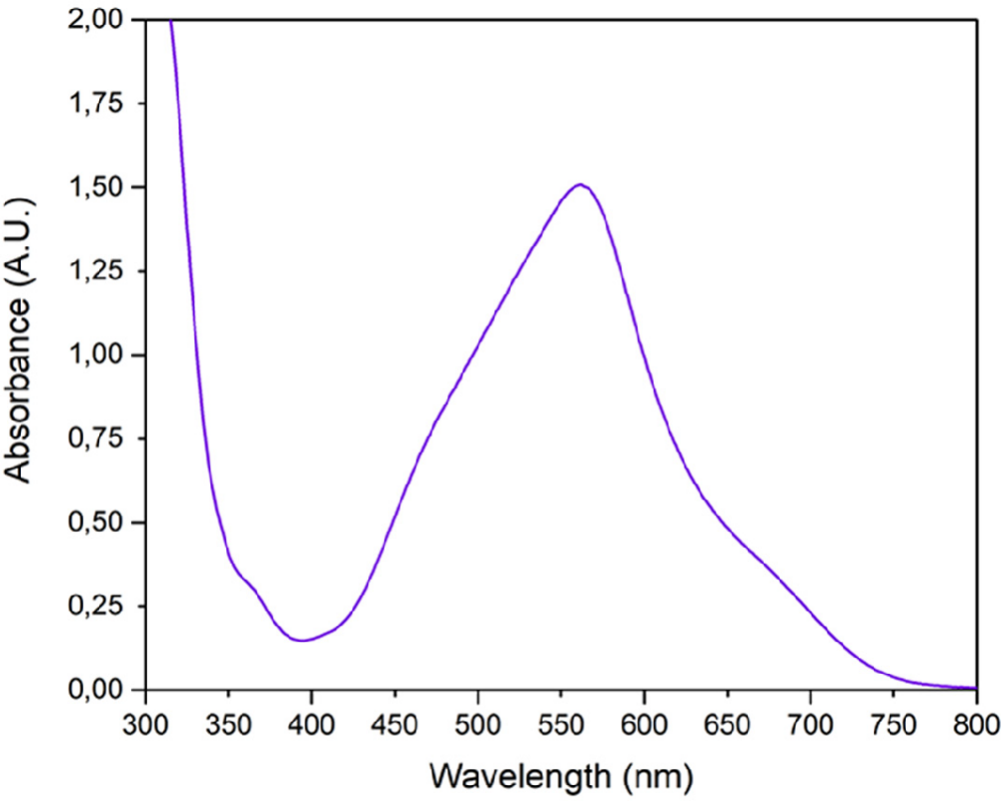
UV–vis spectrum in chloroform of [Ru(dpphen)_2_(η^2^-mal)] (**11**).

Compounds **10**–**15** are all soluble in DMSO and chloroform and have similar UV–vis spectra, characterized by a broad and intense MLCT absorption band in the range 510–560 nm, with shoulders at both lower (453–492 nm) and higher (566–660 nm) wavelengths (Figure 9 and Supporting Information). The absorption maxima of the neutral complexes **10**–**13** are red-shifted by ca. 35 nm compared to the corresponding cationic species **14** and **15**, and those of the dpphen compounds **11**, **13**, and **15** are red-shifted by ca. 15 nm compared to the corresponding phen compounds **10**, **12**, and **14** (Table 1).

**Table 1:** Absorption Maxima and Extinction Coefficients in the UV–Vis Spectra (CHCl_3_) of Complexes **10**–**15**

compound	λ_max_ (nm)	ε (L mol^–1^ cm^–1^)
[Ru(phen)_2_(η^2^-mal)] (**10**)	544	3.4 × 10^3^
[Ru(dpphen)_2_(η^2^-mal)] (**11**)	561	1.9 × 10^4^
[Ru(phen)_2_(η^2^-ox)] (**12**)	544	2.4 × 10^3^
[Ru(dpphen)_2_(η^2^-ox)] (**13**)	560	2.0 × 10^4^
[Ru(phen)_2_(η^2^-acac)]Cl (**14**)	509	1.1 × 10^4^
[Ru(dpphen)_2_(η^2^-acac)]Cl (**15**)	524	1.8 × 10^4^

The ^1^H NMR spectra of compounds **10**–**15** are reported in the Supporting Information with peak assignments. Of note, in the HSQC spectrum the resonances of C2 and C9, i.e., the carbons adjacent to the N atoms, are well distinguished from the others, thus affording unambiguous assignments of all resonances.

### Acid-Assisted Preparation of Bis-Heteroleptic Complexes

**Scheme 5:**
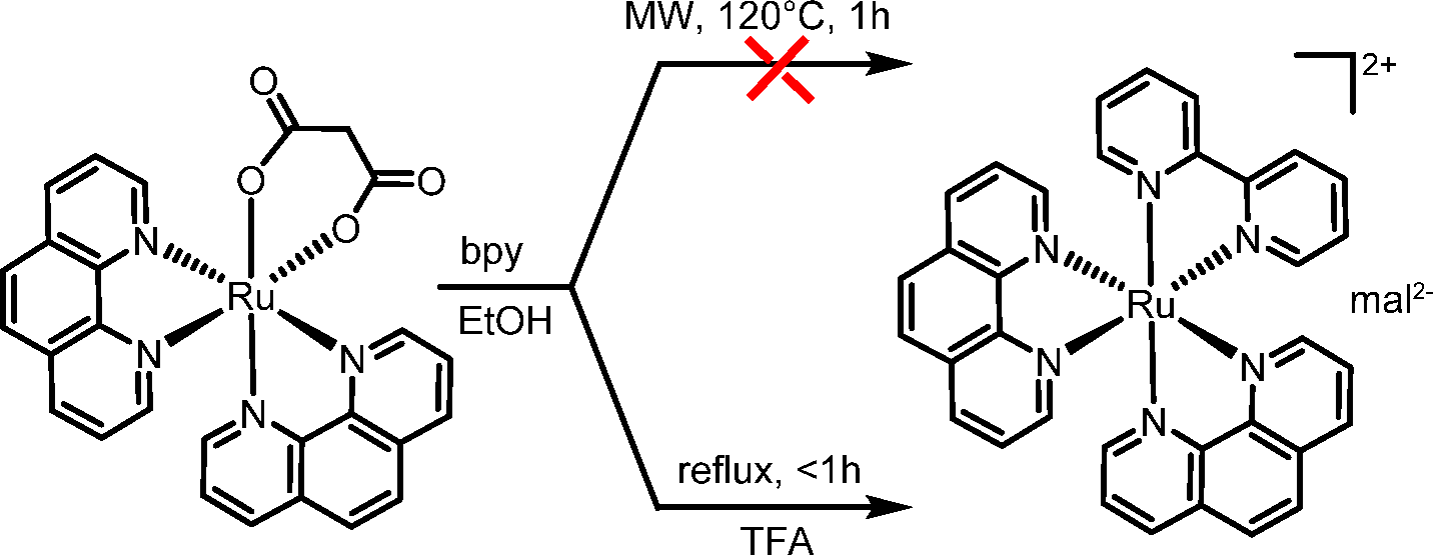
Acid-Assisted Facile and Selective Replacement of Malonate by bpy (TFA = trifluoracetic acid)

The acid-assisted replacement of the O–O chelate with L2 was investigated on selected [Ru(L1)_2_(O–O)]^0/+^ compounds (charge depending on O–O). First, we established that the substitution does not occur readily in the absence of added acid. For example, treatment of [Ru(phen)_2_(η^2^-mal)] (**10**) with 1 equiv of bpy in refluxing ethanol for 6 h showed no significant color change (from deep purple to bright orange-red) typical of the formation of [Ru(phen)_2_(bpy)]^2+^. No reaction was observed either when the mixture was heated for 1 h at 120 °C in the microwave reactor (Scheme 5). Conversely, upon the addition of 10 equiv of trifluoroacetic acid (TFA), 100% substitution was accomplished within 1 h under reflux conditions according to UV–vis spectroscopy (Supporting Information; Scheme 5). The addition of an excess of NH_4_PF_6_ to the final solution afforded [Ru(phen)_2_(bpy)][PF_6_]_2_ (**16**) as an orange precipitate that was recovered in 93% yield. The reaction occurs also at room temperature in ca. 3 days. Similarly, using [Ru(bpy)_2_(η^2^-mal)] (**9**) as a precursor, the complex [Ru(bpy)_2_(phen)][PF_6_]_2_ (**17**) was obtained under the same reaction conditions.

With the precursor [Ru(bpy)_2_(η^2^-acac)][PF_6_] (**8**), we found that the replacement of acac by phen was best accomplished in the presence of HPF_6_ rather than TFA. The addition of 4 equiv of HPF_6_ afforded quantitative formation of [Ru(bpy)_2_(phen)][PF_6_]_2_ (**17**) from **8** in 2.5 h in refluxing ethanol. The bis-heteroleptic compounds **16** and **17** were characterized by UV–vis and ^1^H NMR spectroscopy (Supporting Information). Their NMR resonances are in agreement with those reported in the literature.^74,75^

### Mechanistic Aspects

The ubiquitous formation of [Ru(L1)_3_]^2+^ (as well as that of small amounts of *cis*-[RuCl_2_(L1)_2_] when the monochloride precursors **2**, **4**, and **6** are treated with L1) indicates that the O–O chelates are not sufficiently strong.

The counterintuitive finding that the [Ru(L1)_3_]^2+^/[Ru(L1)_2_(O–O)]^0/+^ ratio increases upon lowering the temperature (i.e., the reaction is less selective)^76^ suggests that the formation of [Ru(L1)_3_]^2+^ from the *cis*-locked precursors occurs in a parallel reaction, rather than in a consecutive step from [Ru(L1)_2_(O–O)]^0/+^. Consistent with this hypothesis, we found that bpy is unable to replace malonate readily from [Ru(phen)_2_(η^2^-mal)] (**10**) even at 120 °C in a MW reactor. In addition, the finding that the selectivity increases upon lowering the concentration suggests that the formation of [Ru(L1)_3_]^2+^ does not depend (dramatically) on the presence of adventitious water in the EtOH solvent (that might favor the dissociation of O–O): in this hypothesis, since at lower concentrations the H_2_O/Ru ratio becomes larger, the opposite trend would have been expected. Consistently, we found that running the reaction in absolute EtOH treated with activated molecular sieves led to no significant improvement in the selectivity.

**Scheme 6:**
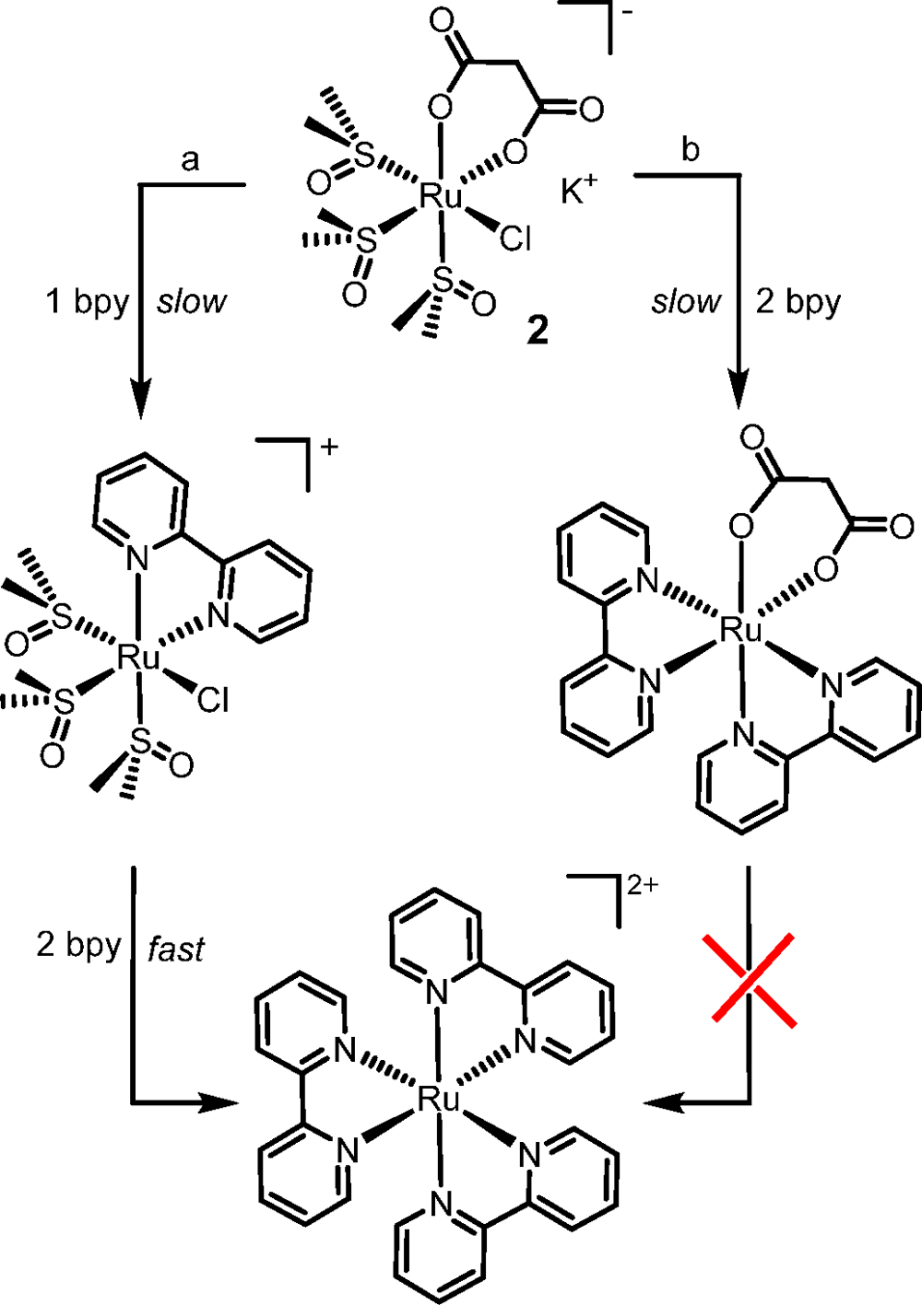
Hypothesis of the Two Parallel Routes Leading to the Mixture of [Ru(L1)_2_(O–O)]^*n*+^ (Branch b) and [Ru(L1)_3_]^2+^ (Branch a) Exemplified for X = Cl^–^, L1 = bpy and O–O = mal

In conclusion, we hypothesize that the formation of [Ru(L1)_3_]^2+^ involves, as a first, relatively slow step, the replacement of O–O in *fac*-[RuCl(DMSO–S)_3_(O–O)]^0/–^ by L1, with formation of an intermediate such as *fac*-[RuCl(DMSO–S)_3_(L1)]^+^ (Scheme 6; similar considerations apply to *fac*-[Ru(DMSO–O)(DMSO–S)_3_(O–O)]^0/+^). The subsequent replacement of the remaining monodentate ligands by two additional L1 molecules must be rather fast. The experimental findings show that at relatively low temperatures (i.e., 80 °C) this parasite reaction (branch a in Scheme 6) is faster than the parallel reaction leading to [Ru(chel)_2_(O–O)]^0/+^ (branch b); however, the rate of this latter, and thus the selectivity of the reaction, increases more rapidly with the temperature.

## Conclusions

In the Introduction, we reviewed the main synthetic routes to heteroleptic polypyridyl Ru(II) complexes, evidencing advantages and limits. Furthermore, we tested experimentally the route that uses *cis*-[RuCl_2_(DMSO)_4_] (**1**) as a precursor, finding that—in general—this complex is not particularly reactive and well-behaved for this type of reaction and that what is reported in the literature for a particular diimine ligand is not always reproducible or automatically extensible even to similar ligands.

Thus, with the purpose of finding better Ru(II) precursors, we investigated a series of six neutral, anionic and cationic *cis*-locked Ru(II)-DMSO complexes (**2**–**7**) of the general formula [Y] *fac*-[RuX(DMSO–S)_3_(O–O)]^*n*^ (where O–O = mal, ox, acac; X = DMSO–O or Cl^–^; *n* = −1/0/+1 depending on the nature and charge of X and O–O; when present, Y = K^+^ or PF_6_^–^) for the two-step synthesis of bis-heteroleptic polypyridyl complexes [Ru(L1)_2_(L2)]^2+^. The chlorido complexes **2**, **4**, and **6** are efficiently obtained in one step from **1** (in 70–80% yields); the synthetic effort for obtaining the more reactive DMSO–O complexes **3**, **5**, and **7** from **1** is higher, requiring silver-assisted chloride abstraction, but yields remain good (60–70%).

We clearly established that in the first step the *cis*-locked precursors **2**–**7** are definitely superior to the unlocked complex **1**, in terms of both reactivity and selectivity. The best results were obtained with the dicarboxylate compounds **2**–**5** that, when treated with model diimine chelating ligands (L1 = bpy, phen, dpphen), afforded in good yield the corresponding [Ru(L1)_2_(O–O)] intermediate, even though contaminated by variable amounts of the [Ru(L1)_3_]^2+^ species. No other byproducts, such as partially substituted derivatives and stereoisomers, were detected. Whereas the unexpected formation of the trischelate dead-end complex is disturbing, we found that its amount can be minimized by carefully adjusting the reaction conditions: in particular, high selectivity toward [Ru(L1)_2_(O–O)] and almost complete conversion of the precursor, also on a 100–200 mg scale, was obtained within minutes when the reactions were performed in absolute ethanol at 150 °C in a microwave reactor. In addition, we found that, depending on the nature of L1, O–O, and concentration, at the end of the reaction the neutral product [Ru(L1)_2_(O–O)] can precipitate spontaneously from the mother liquor, in pure form and acceptable-to-good yields. For example, the less soluble oxalate compounds [Ru(phen)_2_(η^2^-ox)] (**12**) and [Ru(dpphen)_2_(η^2^-ox)] (**13**) were obtained in 70–80% isolated yields (i.e., 50–55% with respect to **1**). When spontaneous precipitation of the disubstituted product does not occur, purification from [Ru(L1)_3_]^2+^—given the charge difference—can be easily accomplished by column chromatography or solvent extraction. By comparison, under the same conditions, compound **1** is much less selective, thus demonstrating that the choice of locking the geometry of the precursor through the introduction of O–O in the coordination sphere of Ru is a valid strategic approach (even though the strength of the “locking ligand” should be improved for further reducing the formation of [Ru(L1)_3_]^2+^). In addition, the use of microwave-assisted conditions was clearly demonstrated as superior to reflux for these reactions.^75^

The second step in the preparation of [Ru(L1)_2_(L2)]^2+^ turned out to be rather straightforward: in the presence of a slight excess of trifluoroacetic acid or HPF_6_, the facile and quantitative replacement of O–O by L2 in [Ru(L1)_2_(O–O)]^0/+^ occurs in refluxing ethanol (or even at lower T).

As a general comment, we believe that, whereas the classical Meyer’s route (i.e., preparation of [Ru(L1)_2_(L2)]^2+^ via [Ru(L1)_2_Cl_2_] in refluxing DMF) is well-suited—despite its limits—for large scale preparations with simple, readily available L1 and L2 ligands (e.g., L1 = bpy), our new approach compares well with other existing methods for small-scale preparations with less-common diimines. In fact, (*i*) *cis*-locked precursors can be prepared in two high-yield steps from commercial hydrated RuCl_3_ (i.e., one step from **1**). (*ii*) By adjusting the reaction conditions, the final [Ru(L1)_2_(L2)][PF_6_]_2_ products can be obtained in pure form in two additional steps with good isolated yields. (*iii*) The acid-assisted nature of the second step makes the insertion of L2 possible at relatively low temperatures, which is advantageous for thermally unstable diimines (unless acid-sensitive) and, in the case of chiral O–O* ligands, for the asymmetric synthesis approach.^36,63^ It has to be stressed that the results described here concern a limited pool of model ligands. Even though the synthetic approach has a general value, the variety of diimine ligands is such that the reaction parameters might need to be adjusted on each specific ligand set (L1 and L2) for achieving the best selectivity and yield.

We are currently investigating the *cis*-locked Ru(II) complexes **2**–**7** as precursors for the preparation of tris-heteroleptic polypyridyl products [Ru(L1)(L2)(L3)]^2+^.

## Experimental Section

### Materials

All chemicals were purchased from Sigma-Aldrich and used as received. Solvents were of reagent grade. The Ru(II) precursor *cis*-[RuCl_2_(DMSO)_4_] (**1**) was prepared as described in ref (51).

### Instrumental Methods

Mono- and bidimensional (^1^H–^1^H COSY, ^1^H–^13^C HSQC) NMR spectra were recorded at room temperature on a Varian 400 or 500 spectrometer (^1^H, 400 or 500 MHz; ^31^P{^1^H}, 202 MHz; ^19^F, 376 MHz). ^1^H chemical shifts were referenced to the peak of residual nondeuterated solvent (δ 7.26 for CDCl_3_ and 2.50 for DMSO-*d*_6_) or were measured relative to the internal standard DSS (δ 0.00) for D_2_O. Carbon resonances were assigned through the HSQC spectra. ESI mass spectra were collected in the positive and negative mode on a PerkinElmer APII spectrometer at 5600 eV. The UV–vis spectra were obtained on an Agilent Cary 60 spectrophotometer, using 1.0-cm-path-length quartz cuvettes (3.0 mL). An Anton Paar 400 microwave reactor (with video-camera) was used for the microwave-assisted reactions performed in 10 or 30 mL vessels. Elemental analyses were performed in the Department of Chemistry of the University of Bologna (Italy).

### X-ray Diffraction

Data collections were performed at the X-ray diffraction beamline (XRD1) of the Elettra Synchrotron of Trieste (Italy) equipped with a Pilatus 2 M image plate detector. The collection temperature was 100 K (nitrogen stream supplied through an Oxford Cryostream 700). The wavelength of the monochromatic X-ray beam was 0.700 Å, and the diffractograms were obtained with the rotating crystal method. The crystals were dipped in N-paratone and mounted on the goniometer head with a nylon loop. The diffraction data were indexed, integrated, and scaled using the XDS code.^77^ The structures were solved by the dual space algorithm implemented in the SHELXT code.^78^ Fourier analysis and refinement were performed by the full-matrix least-squares methods based on F^2^ implemented in SHELXL.^79^ The Coot and SHELXLE programs were used for modeling.^80,81^ Anisotropic thermal motion was allowed for all non-hydrogen atoms. Hydrogen atoms were placed at calculated positions with isotropic factors *U* = 1.2 × Ueq, Ueq being the equivalent isotropic thermal factor of the bonded non-hydrogen atom. Crystal data and details of refinements are in the Supporting Information.

### Preparation of the Complexes

#### [K] *fac*-[RuCl(DMSO–S)_3_(η^2^-mal)] (**2**)

A procedure similar to that described in ref (64) was followed: a 1.0 g amount of *cis*-[RuCl_2_(DMSO)_4_] (**1**; 2.06 mmol) was partially dissolved in 100 mL of methanol. After the addition of 1 equiv of K_2_mal (373.1 mg), the mixture was heated to reflux, affording a pale yellow solution. After 90 min, it was evaporated to an oil and washed with acetone to remove DMSO, affording a pale-yellow solid. The product (a mixture of compound **2** and KCl) was recrystallized from warm ethanol (100 mL); the poorly soluble KCl was removed by filtration of the warm solution over fine paper. The yellow filtrate was evaporated to dryness and the solid washed with acetone and diethyl ether and dried under a vacuum. Yield: 857.3 mg (82%). Anal. Calcd For C_9_H_20_ClO_7_RuS_3_K, MW = 512.06: C, 21.11; H, 3.94. Found C, 20.92; H, 3.85. ^1^H NMR (D_2_O) δ, ppm: 4.01 (d, 1H, mal), 3.42 (s, 6H, DMSO–S), 3.39 (s, 6H, DMSO–S), 3.21 (s, 6H, DMSO–S), 3.15 (d, 1H, mal). The resonances are coincident with those previously reported by us for this complex.^64^ Selected IR absorptions (nujol, cm^–1^): 1589 ν_asymm(COO)_, 1410 ν_symm(COO)_, 1111 ν_S=O(DMSO–S)_. ESI mass spectrum (*m*/*z*): 472.8 [M–K]^−^ (calcd. for C_9_H_20_ClO_7_RuS_3_, 472.91).

#### *fac*-[Ru(DMSO–O)(DMSO–S)_3_(η^2^-mal)] (**3**)

A 500.7 mg amount of *cis*-[RuCl_2_(DMSO)_4_] (**1**; 1.03 mmol) was dissolved in 4.0 mL of DMSO at ca. 100 °C. The addition of 1.1 equiv of Ag_2_mal (360.2 mg) afforded immediately a greyish precipitate of AgCl. After 30 min, the mixture was cooled and the precipitate removed by filtration over a Celite pad and extensively washed with MeOH (where **3** is soluble). The product precipitated spontaneously as a pale-yellow solid from DMSO upon evaporation of the methanol, and its amount was increased by the addition of acetone (10 mL). The product was recrystallized from methanol (50 mL): residual AgCl was removed by filtration and washed with additional MeOH. The filtrate was evaporated to dryness and the solid washed with acetone and diethyl ether and dried under a vacuum. Yield: 375.1 mg (70.6%). Anal. Calcd for C_11_H_26_O_8_RuS_4_, MW = 515.63: C, 25.62; H, 5.08. Found C, 25.91; H, 4.87. ^1^H NMR (D_2_O) δ, ppm: 3.85 (d, 1H, mal), 3.42 (d, 1H, mal), 3.35 (s, 12H, DMSO–S), 3.34 (s, 6H, DMSO–S), 2.84 (s, 6H, DMSO–O). Consistent with the literature, in aqueous solution, the complex is in equilibrium with the aquated species *fac*-[Ru(DMSO–S)_3_(OH_2_)(η^2^-mal)] (**3aq**). ^1^H NMR (D_2_O) δ, ppm: 3.59 (d, 1H, mal), 3.42 (s, 6H, DMSO–S), 3.37 (s, 6H, DMSO–S), 3.23 (s, 6H, DMSO–S), 3.29 (d, 1H, mal). The resonances of **3** and **3aq** are coincident with those previously reported by us for these species.^64^ Selected IR absorptions (nujol, cm^–1^): 1604 ν_asymm(COO)_, 1376 ν_symm(COO)_, 1117 ν_S=O(DMSO–S)_, 933 ν_S=O(DMSO–O)_. ESI mass spectrum (*m*/*z*): 516.9 [M + H]^+^ (calcd. for C_12_H_26_O_8_RuS_4_ 516.96).

#### [K] *fac*-[RuCl(DMSO–S)_3_(η^2^-ox)] (**4**)

The procedure was improved compared to that reported in ref (61). A 502.6 mg amount of *cis*-[RuCl_2_(DMSO)_4_] (**1**; 1.03 mmol) was partially dissolved in 3.0 mL of DMSO in a 10 mL MW vial. After the addition of 2 equiv of K_2_(ox) (344.2 mg, 2.03 mmol), the sealed system was heated at 125 °C for 1 h in a MW reactor.^82^ The final suspension is filtered warm to remove an abundant white precipitate (KCl + residual K_2_(ox)), which is washed with MeOH (where **4** is soluble). Removal of methanol from the filtrate by rotary evaporation, followed by the addition of EtOH (ca. 10 mL) induced the precipitation of the product as a pale-yellow solid, which was removed by filtration and washed with EtOH and diethyl ether and dried under a vacuum. The product was pure **4** according to the ^1^H NMR spectrum. Yield: 352 mg (70%). Anal. Calcd for C_8_H_18_ClKO_7_RuS_3_, MW = 498.02: C, 19.29; H, 3.64. Found C, 19.48; H, 3.77. ^1^H NMR (D_2_O) δ, ppm: 3.45 (s, 6H, DMSO–S), 3.43 (s, 6H, DMSO–S), 3.22 (s, 6H, DMSO–S). The resonances of **4** are coincident with those previously reported by us for this species.^64^ Selected IR absorptions (nujol, cm^–1^): 1667 ν_asymm(COO)_, 1388 ν_symm(COO)_, 1109 ν_S=O(DMSO–S)_. ESI mass spectrum (*m*/*z*): 458.8 [M–K]^−^ (calcd. for C_8_H_18_ClO_7_RuS_3_, 458.89).

#### *fac*-[Ru(DMSO–O)(DMSO–S)_3_(η^2^-ox)] (**5**)

The procedure was improved compared to that reported in ref (64). A 500.1 mg amount of *cis*-[RuCl_2_(DMSO)_4_] (**1**; 1.03 mmol) was partially dissolved in 3.0 mL of DMSO. After the addition of 2.1 equiv of AgNO_3_ (372.1 mg, 2.19 mmol), the system was heated to 60 °C for 30 min. The whitish precipitate of AgCl was removed by filtration and thoroughly washed with MeOH. A 417.3 mg amount of Na_2_(ox) (3 equiv, 3.11 mmol) was added to the filtrate after removal of the MeOH by rotary evaporation, and the mixture was heated to 60 °C for 24 h. The progressive formation of a white precipitate was observed. After cooling the mixture, the precipitate was removed by filtration, washed with acetone and diethyl ether, and dried under a vacuum. It was recrystallized from ethanol (50 mL) at room temperature: residual AgCl was removed by filtration and washed with additional ethanol. The filtrate was evaporated to dryness and the solid washed with acetone and diethyl ether and dried under a vacuum. Yield: 358.2 mg (69.3%). Anal. Calcd for C_10_H_24_O_8_RuS_4_, MW = 501.60: C, 23.95; H, 4.82. Found C, 23.61; H, 4.67. ^1^H NMR (D_2_O) δ, ppm: 3.41 (s, 6H, DMSO–S), 3.37 (s, 6H, DMSO–S), 3.21 (s, 6H, DMSO–S), 2.80 (s, 6H, DMSO–O). The resonances of **5** are coincident with those previously reported by us for this species.^64^ Selected IR absorptions (nujol, cm^–1^): 1661 ν_asymm(COO)_, 1377 ν_symm(COO)_, 1106 ν_S=O(DMSO–S)_, 933 ν_S=O(DMSO–O)_. ESI mass spectrum (*m*/*z*): 306.8 [M + K – 3DMSO]^+^ (calcd for C_4_H_6_O_5_RuSK 306.86).

#### *fac*-[RuCl(DMSO–S)_3_(η^2^-acac)] (**6**)

A 500.0 mg amount of *cis*-[RuCl_2_(DMSO)_4_] (**1**; 1.03 mmol) was partially dissolved in 25 mL of methanol. After the addition of 1 equiv of Na(acac) (122.5 mg), the mixture was heated to reflux, affording a pale yellow solution. After 2 h, the obtained yellow solution was filtered over fine paper for removing a white suspension of NaCl formed during the reaction. The yellow solution was evaporated to dryness, and the initially sticking solid was repeatedly crushed in a sonicator with portions of diethyl ether until a yellow powder was obtained. It was collected by filtration, washed with diethyl ether, and dried under a vacuum. The product was recrystallized from chloroform: residual NaCl was removed by filtration, the filtrate evaporated to dryness, and the solid washed with diethyl ether and dried under a vacuum. Yield: 358.2 mg (69.3%). Yield: 400.7 mg (82%). Crystals of **6** suitable for X-ray analysis were obtained upon layering diethyl ether on top of a chloroform solution of the complex. Anal. Calcd for C_11_H_25_ClO_5_RuS_3_, MW = 470.02: C, 28.11; H, 5.36. Found: C, 27.86; H, 5.21. ^1^H NMR (CDCl_3_) δ, ppm: 5.53 (s, 1H, acac), 3.40 (s, 6H, DMSO–S), 3.36, (s, 6H, DMSO–S), 3.18 (s, 6H, DMSO–S), 2.09 (s, 6H, CH_3_ acac). Selected IR absorptions (nujol, cm^–1^): 1578 ν_asymm(CO)_, 1521 ν_symm(CO)_, 1122, 1099 ν_S=O(DMSO–S)_. ESI-MS (*m*/*z*): 491.9 [M + Na]^+^ (calcd. for C_11_H_25_ClO_5_RuS_3_Na, 492.0).

#### *fac*-[Ru(DMSO–O)(DMSO–S)_3_(η^2^-acac)][PF_6_] (**7**)

A 700.4 mg amount of *fac*-[RuCl(DMSO–S)_3_(η^2^-acac)] (**6**; 1.43 mmol) was partially dissolved in a mixture of DMSO (0.3 mL) and acetone (20 mL). After the addition of 1.1 equiv of AgPF_6_ (381 0.0 mg, 1.57 mmol), the system was stirred at 30 °C for 2.5 h. The whitish precipitate of AgCl was removed by filtration over a Celite pad and thoroughly washed with acetone. Removal of acetone from the filtrate at reduced pressure afforded a dark-yellow oil; dropwise addition of chloroform induced the formation of a white precipitate that was filtered and abundantly washed with chloroform. The product was recrystallized from ethanol (50 mL) at room temperature: residual AgCl was removed by filtration and washed with additional ethanol. The filtrate was evaporated to dryness and the solid washed with chloroform and diethyl ether and dried under a vacuum. Yield: 598.8 mg (63.6%). Crystals of **7** suitable for X-ray analysis were obtained upon layering *n*-hexane on top of an acetone solution of the complex. Anal. Calcd for C_13_H_31_O_6_RuPS_4_F_6_, MW = 657.66: C, 23.74; H, 4.75. Found: C, 23.89; H, 4.51. ^1^H NMR (CD_3_NO_2_) δ, ppm: 5.92 (s,1H, acac), 3.37 (s, 6H, DMSO–S), 3.33 (s, 6H, DMSO–S), 3.06 (s, 6H,DMSO–S), 2.73 (s, 6H, DMSO–O), 2.23 (s, 6H, CH_3_ acac). ^31^P NMR(acetone-*d*_6_) δ, ppm: −144.5 (septet). Selected IR absorptions (nujol, cm^–1^): 1566 ν_asym(COO)_; 1522ν_sym(COO)_; 1123, 1093 ν_S=O (DMSO–S)_; 933 ν_S=O (DMSO–O)_. ESI-MS (*m*/*z*): 356.9 [M – 2DMSO]^+^ (calcd. for C_9_H_19_O_4_RuPS_2_F_6_, 356.97).

### Synthesis of [Ru(L1)_2_(O–O)]^0/+^ Compounds

Some representative examples of the many MW-assisted reactions performed between precursors **2**–**7** and a diimine ligand (L1 = bpy, phen, dpphen) are described below. The reactions with bpy were performed on a relatively small scale for optimizing the conversion and selectivity. The reaction products were analyzed as follows: when no precipitate was found at the end of the run, after TLC analysis (typical eluent CHCl_3_/MeOH 7:3), the solvent was removed by rotary evaporation and the residual oil dissolved in DMSO-*d*_6_ for NMR investigation. For the more concentrated solutions ([Ru] > 6.1 mM), the final ink-dark solution was first diluted with 50 mL of EtOH to make sure that no fine precipitate was present,^67^ and the above-described analysis was performed on a sample taken from the diluted solution. In the case of precursor **7**, the NMR analysis was performed on the mother liquor after removal of the precipitate of [Ru(bpy)_2_(η^2^-acac)][PF_6_] (**8**) and [Ru(bpy)_3_][PF_6_]_2_ (see text), and thus the conversion percentage was not established.

The larger scale MW-assisted reactions with phen and dpphen were performed on precursors **2**, **4**, and **6** at 150 °C for 1 h; the concentration of the Ru precursor was in the 12–20 mM range. The workup depended on the presence (or absence) of a precipitate at the end of the run. Precursors **2** and **4** afforded a dark purple precipitate, which was removed by filtration, washed with ethanol and diethyl ether, and dried under a vacuum. For increasing the amount of precipitate in some cases (e.g., with the more soluble dpphen), the mother liquor was concentrated to circa half-volume and stored at 4 °C for 24 h prior to filtration. According to ^1^H NMR analysis, the raw precipitate was, in each case, the pure [Ru(L1)_2_(O–O)] compound (**10**–**14**, L1 = phen or dpphen, O–O = ox or mal). Recrystallization from chloroform at room temperature was sufficient to remove the coprecipitated KCl (except in the case of **12**, see below). The presence of the [Ru(L1)_3_]^2+^ species in the filtrate was ascertained by TLC analysis. Using **6** as a precursor, no product precipitated spontaneously from the solution, and both TLC and NMR spectroscopy indicated that it contained a mixture of di- and trisubstituted Ru compounds. In this case, the [Ru(L1)_2_(η^2^-acac)][Cl] species, (**14**, L1 = phen; **15** L1 = dpphen) were obtained in pure form by column chromatography on silica gel. Essential details of each preparation are given below (see Supporting Information for proton labeling schemes).

#### [Ru(bpy)_2_(η^2^-acac)][PF_6_] (**8**)

Starting materials: 60.0 mg of *fac*-[RuCl(DMSO–S)_3_(η^2^-acac)] (**6**; 0.128 mmol) and 41.9 mg (2.1 equiv) of bpy in 5.0 mL of EtOH. At the end of the run, the solvent was removed by evaporation affording an ink-dark purple mixture, which was dissolved in ca. 4 mL of water. The addition of an excess of NH_4_PF_6_ (400.0 mg, ca. 20 equiv) afforded the immediate precipitation of a dark-brown precipitate. The mixture was centrifuged, and the precipitate, after removal of the supernatant, was washed with water (×3). The solid mixture was extracted with 50 mL of chloroform and the residual dark-red solid removed by filtration: it is pure [Ru(bpy)_3_][PF_6_]_2_ according to the ^1^H NMR spectrum in DMSO-*d*_6_ (Supporting Information). Removal of the solvent from the deep-purple filtrate afforded **8** as a dark solid that was suspended in diethyl ether and recovered by filtration and dried under a vacuum. Yield: 55.3 mg (65.0%). ^1^H NMR (DMSO-*d*_6_) δ, ppm: 8.75 (d, 2H,H6′), 8.63 (m, 4H, H3+ H3′), 8.17 (m, 2H, H5′), 7.85 (t, 2H, H4), 7.74 (t, 2H, H4′) 7.69 (d, 2H, H6), 7.22 (t, 2H, H5), 5.35 (s, 1H, acac), 1.78 (s, 6H, acac). UV–vis (CHCl_3_), λ_max_ (ε, L mol^–1^ cm^–1^): 372 (3.1 × 10^3^), 530 (3.0 × 10^3^) nm. ESI-MS (*m*/*z*): 513.1 [M – PF_6_] (calcd. for C_25_H_23_N_4_O_2_Ru 513.6).

#### [Ru(bpy)_2_(η^2^-mal)] (**9**)

Starting materials: 50.0 mg of [K]*fac-*[RuCl(DMSO–S)_3_(η^2^-mal)] (**2**; 0.098 mmol) and 30.6 mg (2 equiv) of bpy in 5.0 mL of EtOH. At the end of the run, the solvent was removed by evaporation, affording a dark purple mixture. Column chromatography on silica gel (eluent CHCl_3_/MeOH 7:3) afforded pure **9** in the first band. ^1^H NMR (CDCl_3_) δ, ppm: 9.46 (d, 2H, H6), 8.22 (d, 2H, H3), 8.06 (d, 2H, H3′), 7.94 (t, 2H, H5), 7.63 (t, 2H, H4), 7.59 (d, 2H, H6′), 7.56 (t, 2H, H4′), 6.95 (t, 2H, H5′), 3.39 (s, 2H, mal). UV–vis (CHCl_3_), λ_max_ (ε, L mol^–1^ cm^–1^) = 370 (3.0 × 10^3^), 530 (3.1 × 10^3^) nm. ESI-MS (*m*/*z*): 517.0 [M + H]^+^ (calcd. for C_23_H_19_N_4_O_4_Ru 517.04).

#### [Ru(phen)_2_(η^2^-mal)] (**10**)

Starting materials: 50.0 mg of [K]*fac-*[RuCl(DMSO–S)_3_(η^2^-mal)] (**2**; 0.098 mmol) and 35.1 mg (2 equiv) in 5.0 mL of EtOH. Yield: 18.6 mg (34%). ^1^H NMR (DMSO-*d*_6_) δ, ppm: 9.57 (d, 2H, H9), 8.78 (d, 2H, H7), 8.32–8.37 (m, 4H, H6 + H4), 8.27 (dd, 2H, H8), 8.21 (d, 2H, H5), 7.89 (d, 2H, H2), 7.44 (dd, 2H, H3), 2.94 (s, 2H, mal). UV–vis (CHCl_3_), λ_max_ (ε, L mol^–1^ cm^–1^): 544 (3.4 × 10^3^) nm. ESI-MS (*m*/*z*): 565.0 [M + H]^+^ (calcd. for C_27_H_19_N_4_O_4_Ru 564.54).

#### [Ru(dpphen)_2_(η^2^-mal)] (**11**)

Starting materials: 200.0 mg of [K]*fac-*[RuCl(DMSO–S)_3_(η^2^-mal)] (**2**; 0.390 mmol) and 260.0 mg (2 equiv) of dpphen in 22.0 mL of EtOH. Yield of **11**: 122.7 mg (36%). ^1^H NMR (DMSO-*d*_6_) δ, ppm: 9.70 (d, 2H, H9), 8.30 (d, 2H, H8), 8.25 (d, 2H, H6), 8.17 (d, 2H, H2), 8.11 (d, 2H, H5), 7.87 (d, 4H, H*o*′), 7.73 (t, 4H, H*m*′), 7.66 (t, 2H, H*p*′), 7.52–7.62 (m, 10H, H*o*,*m*,*p*), 7.50 (d, 2H, H3), 3.02 (s, 2H, CH_2_–mal). UV–vis (CHCl_3_), λ_max_ (ε, L mol^–1^ cm^–1^): 561 (1.9 × 10^4^) nm. ESI-MS (*m*/*z*): 869.1 [M + H]^+^ (calcd. for C_51_H_35_N_4_O_4_Ru 868.9), 891.1 [M + Na]^+^ (calcd for C_51_H_34_N_4_O_4_RuNa 890.9).

#### [Ru(phen)_2_(η^2^-ox)] (**12**)

Starting materials: 62.24 mg (0.13 mmol) of [K] *fac*-[RuCl(DMSO–S)_3_(η^2^-ox)] (**4**) and 45.05 mg (2 equiv) of phen in 8.0 mL of EtOH. Since 12 is sparingly soluble in chloroform, the raw product was washed with water to remove KCl and then thoroughly dried under a vacuum. Yield: 47.6 mg (69%).^1^H NMR (DMSO-*d*_6_) δ, ppm: 9.32 (d, 2H, H9), 8.79 (d, 2H, H7), 8.32–8.40 (m, 4H, H6 + H4), 8.21–8.28 (m, 4H, H8 + H5), 7.91 (d, 2H, H2), 7.44 (dd, 2H, H3). UV–vis (CHCl_3_), λ_max_ (ε, L mol^–1^ cm^–1^): 544 (2.4 × 10^3^) nm. ESI-MS (*m*/*z*): 551.0 [M + H]^+^ (calcd for C_26_H_17_N_4_O_4_Ru 550.51), 589.0 [M + K]^+^ (calcd. for C_26_H_17_N_4_O_4_RuK, 588.61).

#### [Ru(dpphen)_2_(η^2^-ox)] (**13**)

Starting materials: 200.0 mg (0.40 mmol) of [K] *fac*-[RuCl(DMSO–S)_3_(η^2^-ox)] (**4**) and 267.0 mg (2 equiv) of dpphen in 22.0 mL of EtOH. Yield: 274.4 mg (80%). ^1^H NMR (DMSO-*d*_6_) δ, ppm: 9.44 (d, 2H, H9), 8.29 (d, 2H, H8), 8.26 (d, 2H, H6), 8.20 (d, 2H, H2), 8.14 (d, 2H, H5), 7.82 (d, 4H, H*o*′), 7.73 (t, 4H, H*m*′), 7.65 (t, 2H, H*p*′), 7.52–7.62 (m, 10H, H*o*,*m*,*p*), 7.49 (d, 2H, H3). UV–vis (CHCl_3_), λ_max_ (ε, L mol^–1^ cm^–1^): 560 (2.0 × 10^4^) nm. ESI-MS (*m*/*z*): 855.1 [M + H]^+^ (calcd for C_50_H_33_N_4_O_4_Ru 854.9), 877.1 [M + Na]^+^ (calcd. for C_50_H_32_N_4_O_4_RuNa 876.9).

#### [Ru(phen)_2_(η^2^-acac)][Cl] (**14**)

Starting materials: 188.8 mg (0.40 mmol) of *fac*-[RuCl(DMSO–S)_3_(η^2^-acac)] (**6**) and 144.2 mg (2 equiv) of phen in 22 mL of EtOH. At the end of the run, the solvent was removed by evaporation, affording a dark purple mixture. Column chromatography on silica gel (eluent CHCl_3_/MeOH 9:1) afforded pure **14** in the first band. Yield: 110.5 mg (46%). For comparative purposes, the [Ru(phen)_3_][Cl]_2_ compound was then eluted by increasing the eluent polarity to 8.5:1.5. ^1^H NMR (DMSO–d6) δ, ppm: 9.13 (d, 2H, H9), 8.83 (d, 2H, H7), 8.42 (d, 2H, H4), 8.36 (d, 2H, H6), 8.24 (d, 2H, H5), 8.18 (dd, 2H, H8), 7.96 (d, 2H, H2), 7.47 (dd, 2H, H3), 5.42 (s, 1H, acac), 1.78 (s, 6H, acac). UV–vis (CHCl_3_), λ_max_ (ε, L mol^–1^ cm^–1^): 509 (1.1 × 10^4^) nm. ESI-MS (*m*/*z*): 561.1 [M–Cl]^+^ (calcd for C_29_H_23_N_4_O_2_Ru 560.6), 381.0 [M–Cl–phen]^+^ (calcd. for C_17_H_15_N_2_O_2_Ru 380.39).

#### [Ru(dpphen)_2_(η^2^-acac)][Cl] (**15**)

Starting materials: 200.0 mg (0.42 mmol) of *fac*-[RuCl(DMSO–S)_3_(η^2^-acac)] (**6**) and 281.7 mg (2 equiv) of dpphen in 22.0 mL of EtOH. At the end of the run, the solvent was removed by evaporation, affording a dark purple mixture. Column chromatography on silica gel (eluent acetone/EtOH 91:9) afforded pure **15** in the first band. Yield: 160.7 mg (42%). ^1^H NMR (DMSO-*d*_6_) δ, ppm: 9.25 (d, 2H, H9), 8.26 (d, 2H, H6), 8.20 (2d, 4H, H2, H8), 8.14 (d, 2H, H5), 7.85 (d, 4H, H*o*′), 7.74 (t, 4H, H*m*′), 7.67 (t, 2H, H*p*′), 7.54–7.63 (m, 10H, H*o*,*m*,*p*), 7.51 (d, 2H, H3), 5.51 (s, 1H, acac), 1.88 (s, 6H, acac). UV–vis (CHCl_3_), λ_max_ (ε, L mol^–1^ cm^–1^): 524 (1.8 × 10^4^) nm. ESI-MS (*m*/*z*): 865.2 [M – Cl]^+^ (calcd. for C_53_H_39_N_4_O_2_Ru 864.99).

### Synthesis of [Ru(L1)_2_(L2)][PF_6_]_2_ Compounds^75^

#### [Ru(phen)_2_(bpy)][PF_6_]_2_ (**16**)

A 10 mg amount of [Ru(phen)_2_(η^2^-mal)] (**10**; 0.018 mmol) and 2.8 mg of bpy (1 equiv) were dissolved in 5 mL of EtOH at reflux, and 10 equiv of TFA (15 μL) was added. The solution gradually turned from purple to bright red-orange. After 1 h, the solvent was removed by evaporation, and ca. 10 equiv of NH_4_PF_6_ dissolved in 2 mL of water was added, affording an orange-red precipitate that was filtered, washed with water and diethyl ether, and dried under a vacuum. Yield: 10.2 mg (93%). ^1^H NMR (CD_3_CN) δ, ppm: (p = phen, b = bpy): 8.65 (d, 2H, H4_p_), 8.55 (d, 2H, H7_p_), 8.52 (d, 2H, H3,3_b_′), 8.24 (m, 4H, H5,6_p_), 8.20 (dd, 2H, H2_p_), 8.03 (t, 2H, H4,4_b_′), 7.88 (dd, 2H, H9_p_), 7.79 (dd, 2H, H3_p_), 7.67 (d, 2H, H6,6_b_′), 7.56 (dd, 2H, H8_p_), 7.27 (t, 2H, H5,5_b_′). ^13^C NMR from the HSQC spectrum (CD_3_CN) δ, ppm: 153.7 (C2_p_), 153.5 (C9_p_), 153.1 (C2_b_), 138.5 (C4_b_), 138.4 (C4_b_), 137.7 (C4_p_), 137.5 (C7_p_), 128.9 (C6_p_), 128.9 (C6_p_), 128.1(C5_b_), 126.8 (C3_p_), 126.8 (C8_p_), 125.1 (C3_b_). UV–vis (CH_3_CN), λ_max_ (ε, L mol^–1^ cm^–1^): 265 (4.5 × 10^4^), 287 (4.3 × 10^4^), 450 (1.0 × 10^4^) nm. ESI-MS (*m*/*z*): 763.2 [M −PF_6_]^+^ (calcd for C_34_H_24_F_6_N_6_PRu 763.1).

#### [Ru(bpy)_2_(phen)][PF_6_]_2_ (**17**)

The same reaction conditions as reported above for complex **16** were used. Starting materials: A 10 mg amount of [Ru(bpy)_2_(η^2^-mal)] (**9**; 0.02 mmol) and 3.5 mg of phen (1 equiv). Yield: 15.6 mg (94%). ^1^H NMR (CD_3_CN) δ, ppm: (p = phen, b = bpy): 8.61 (dd, 2H, H4,7_p_), 8.52 (dd, 2H, H3_b_′), 8.48 (dd, 2H, H3_b_), 8.24 (s, 2H, H5,6_p_), 8.09 (m, 4H, H4_b_′ + H2,9_p_), 7.98 (t, 2H, H4_b_), 7.84 (dd, 2H, H6_b_′), 7.73 (dd, 2H, H3,8_p_), 7.52 (dd, 2H, H6_b_), 7.45 (t, 2H, H5_b_′), 7.21 (t, 2H, H5_b_). ^13^C NMR from the HSQC spectrum (CD_3_CN) δ, ppm: 153.3 (C2,9_p_), 152.9 (C6_b_′), 152.7 (C6_b_), 138.6 (C4_b_′), 138.4 (C4_b_), 137.6 (C4′_p_), 128.9 (C5,6_p_), 128.3 (C5_b_′), 128.3 (C5_b_), 126.9 (C3,8_p_), 125.1 (C3_b_), 125.1 (C3_b_′). ^31^P{^1^H} NMR (CD_3_CN) δ, ppm: −144.63 (septet). ^19^F NMR (CD_3_CN) δ, ppm: −72.91 (d). UV–vis (CHCl_3_), λ_max_ (ε, L mol^–1^ cm^–1^): 449 (1.2 × 10^4^) nm. ESI-MS (*m*/*z*): 739.0 [M – PF_6_]^+^ (calcd for C_32_H_24_F_6_N_6_PRu 739.6).

Alternatively, compound **17** was also prepared on a smaller scale from [Ru(bpy)_2_(η^2^-acac)][PF_6_] (**8**): a 20.0 mg amount of **8** (0.030 mmol) and 1.1 equiv of phen (5.4 mg) were refluxed in 5 mL of EtOH after the addition of 22 μL of a 55% aqueous solution of HPF_6_ (4 equiv). The color change from purple to red-orange occurred within 2.5 h. Dropwise addition of diethyl ether to the final solution (concentrated to ca. 2 mL) to near cloudiness afforded the slow precipitation of red-orange microcrystals that, according to NMR and UV–vis spectroscopy, were pure [Ru(bpy)_2_(phen)][PF_6_]_2_ (**17**). In this case, the yield was not measured.

## Supporting Information Available

Additional comments on the reactivity of **1** with phen and dppz, X-ray structure of compound **c**, mono- and bidimensional NMR spectra and UV–visible spectra for the reported complexes, NMR data on the reactions of the *cis*-locked precursors with bpy showing the effects of different parameters on the yield and selectivity (PDF)

## Accession Codes

CCDC 2058363–2058368 contain the supplementary crystallographic data for this paper. These data can be obtained free of charge via www.ccdc.cam.ac.uk/data_request/cif, or by emailing data_request@ccdc.cam.ac.uk, or by contacting The Cambridge Crystallographic Data Centre, 12 Union Road, Cambridge CB2 1EZ, UK; fax: +44 1223 336033.
