# Supplementary material for: cis-Locked Ru(II)-DMSO Precursors for the Microwave-Assisted Synthesis of Bis-Heteroleptic Polypyridyl Compounds
Source: Inorg Chem. 2021 Apr 28;60(10):7180–95. doi: 10.1021/acs.inorgchem.1c00240 (PMC8154425; doi:10.1021/acs.inorgchem.1c00240)
Supplement: Supplementary file 1 — ic1c00240_si_001.pdf [file ic1c00240_si_001.pdf]

Supporting Information

Cis-locked Ru(II)-dmso precursors for the microwave-assisted synthesis of bis-heteroleptic polypyridyl compounds.

Alessio Vidal,[a] Rudy Calligaro,[a,†] Gilles Gasser,[b] Roger Alberto,[c] Gabriele Balducci,[a] Enzo Alessio[a]*

[a] Department of Chemical and Pharmaceutical Sciences, University of Trieste, Via L. Giorgieri 1, 34127 Trieste, Italy.

[b] Chimie ParisTech, PSL University, CNRS, Institute of Chemistry for Life and Health Sciences, Laboratory for Inorganic Chemistry, 75005 Paris, France.

[c] Department of Chemistry, University of Zurich, Winterthurerstrasse 190, CH-8057 Zurich, Switzerland.

[†]Current address: Department of Chemical Sciences and Technologies, University of Udine, Via Cotonificio 108, 33100 Udine, Italy.

The reactions of *cis*-[RuCl$_2$(dmso)$_4$] (1) with the model diimine chelating ligands phen and dppz

a) Reactions performed in refluxing chloroform.

Complex **1** was treated with one equiv. of phen in refluxing chloroform for 1h, i.e. in the conditions that, according to Grätzel and co-workers,[S1] and Spiccia and co-workers,[S2] are expected to afford selectively the mono-substituted complex *cis,cis*-[RuCl$_2$(dmso-S)$_2$(phen)] in good yield. Conversely, according to ^{1}H NMR analysis (Figure S1), we obtained a ca. 1:1 mixture of the stereoisomers *cis,cis*-[RuCl$_2$(dmso-S)$_2$(phen)] (**a**) and *trans,cis*[-RuCl$_2$(dmso-S)$_2$(phen)] (**b**), together with a good amount of unreacted precursor **1** (Scheme S1).

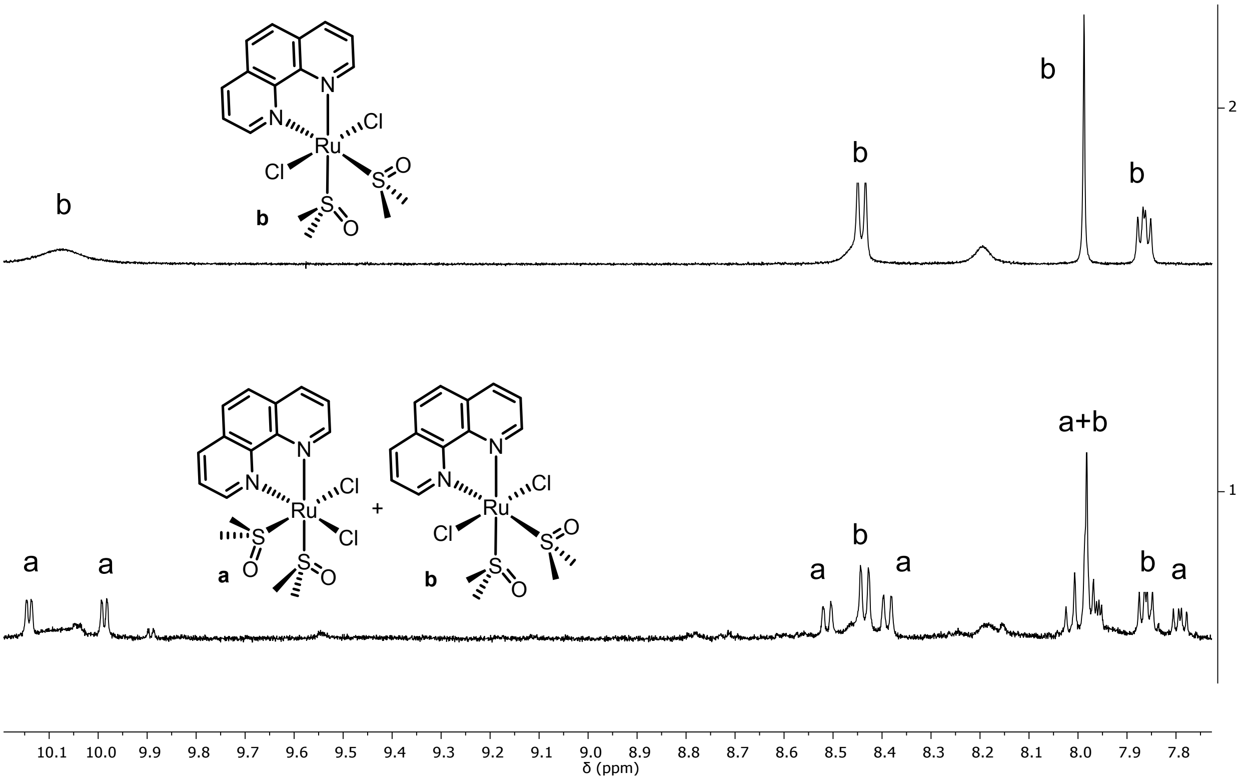

Figure S1. Aromatic region of the ^{1}H NMR spectrum (CDCl$_3$) of: (top) complex *trans,cis*-[RuCl$_2$(dmso-S)$_2$(phen)] (**b**); (bottom) the reaction mixture obtained by treatment of **1** with one equiv. of phen in refluxing CHCl$_3$ for 1h.

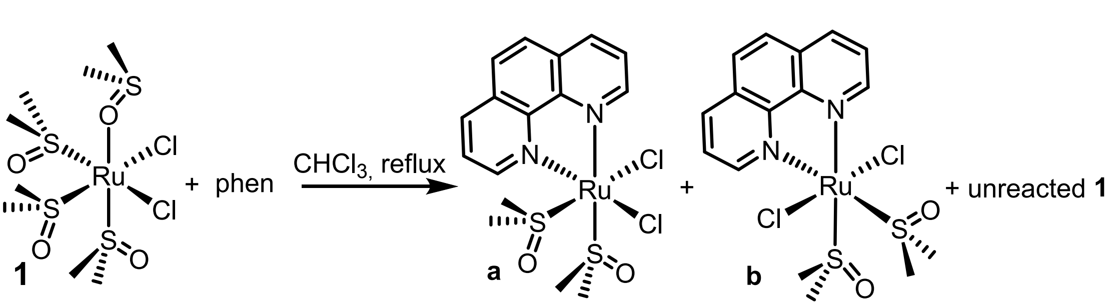

Scheme S1. The reactivity of *cis*-[RuCl$_2$(dmso)$_4$] (**1**) with 1 equiv. of phen in refluxing chloroform.

The NMR spectrum of the more symmetrical isomer **b** (four aromatic resonances, 2H each, and a singlet for two dmso-S ligands) is compatible also with the third stereoisomer *cis,trans*-[$RuCl_2$(dmso-S)$_2$(phen)] (**c**) (Figure S2).

For comparative purposes, the stereoisomer **b** was selectively prepared by treatment of *trans*-[$RuCl_2$(dmso-S)$_4$] (**1t**), the stereo and linkage isomer of **1**, with phen at room temperature (its NMR spectrum is shown in Figure S1). Complex **1t**, in fact, is known to replace selectively two adjacent dmso-S ligands leaving unchanged the geometry of the Ru(II) fragment (Scheme S2). We also found that, consistent with what previously observed for the corresponding bpy complex,[S3] compound **b** is not stable in chloroform solution at room temperature and within 24 hours isomerizes to a mixture of **a** and of the other symmetrical isomer **c**. From this solution we managed to get crystals of **c** suitable for X-ray analysis that afforded the molecular structure of the complex (Figure S3).

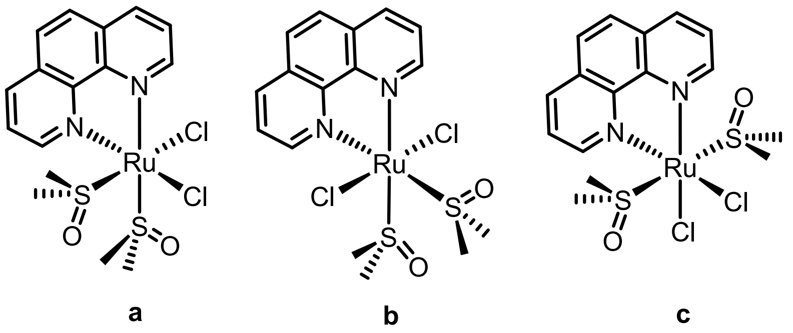

Figure S2. The three stereoisomers *cis,cis*-[$RuCl_2$(dmso-S)$_2$(phen)] (**a**), *trans,cis*-[$RuCl_2$(dmso-S)$_2$(phen)] (**b**), and *cis,trans*-[$RuCl_2$(dmso-S)$_2$(phen)] (**c**).

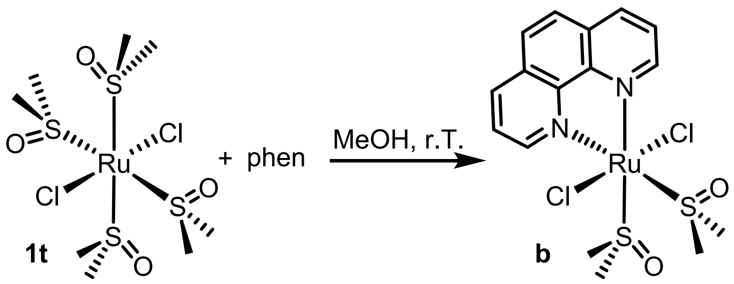

Scheme S2. Preparation of *trans,cis*-[$RuCl_2$(dmso-S)$_2$(phen)] (**b**) from *trans*[-$RuCl_2$(dmso-S)$_4$] (**1t**) in methanol at room temperature.

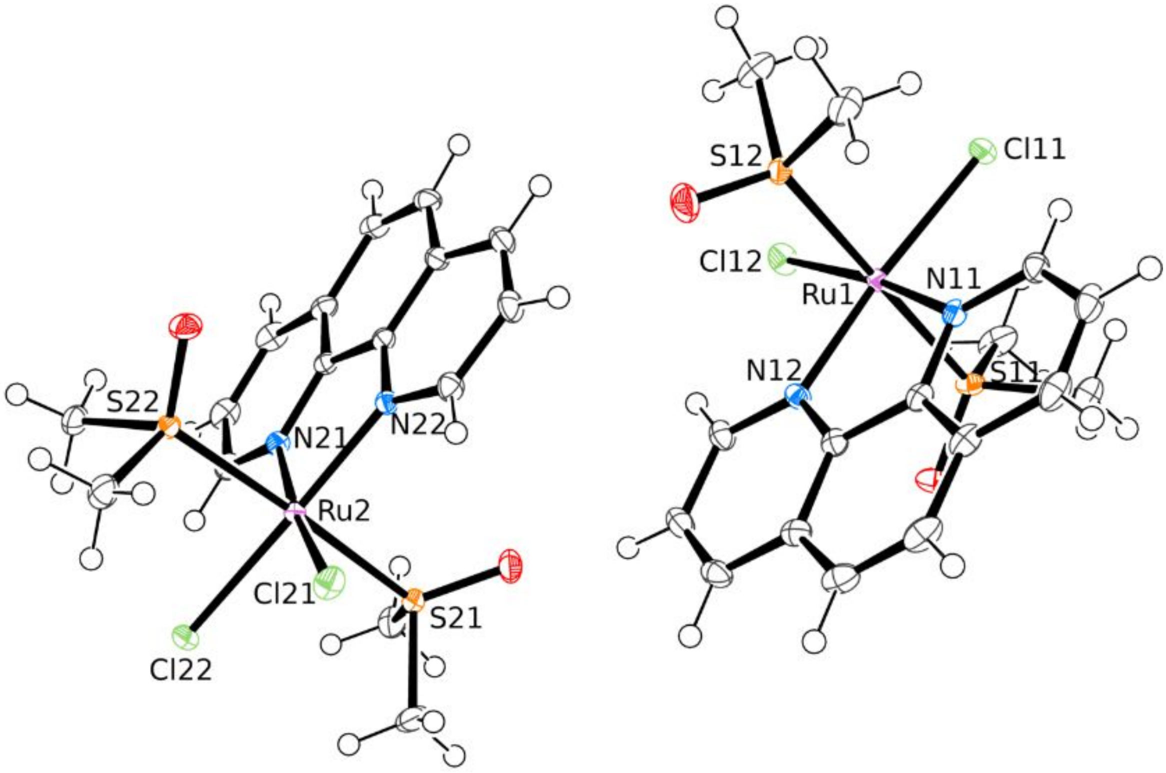

Figure S3. ORTEP representation (50% probability ellipsoids) of stereoisomer *cis,trans*-[$RuCl_2(dmso\text{-}S)_2(phen)$] (**c**).

b) Reactions performed in refluxing ethanol.

Complex **1** was treated with one equiv. of phen in refluxing ethanol for 2h, i.e. in the conditions that – with the similar ligand dppz – according to Burke and Keyes afforded selectively the mono-substituted complex *cis*,*cis*-[$RuCl_2(dmso\text{-}S)_2(dppz)$] in good yield.[S4] The orange product that precipitated spontaneously from the cooled solution turned out to be, according to NMR analysis, a ca. 5:1 mixture of *cis,cis*-[$RuCl_2(dmso\text{-}S)_2(phen)$] (**a**) and *cis,trans*-[$RuCl_2(dmso\text{-}S)_2(phen)$] (**c**) (see below Figure S4). The mother liquor, beside **a** and **c**, contained the disubstituted charged complex *cis*-[$RuCl(dmso\text{-}S)(phen)_2$]Cl (**d**). The proton NMR spectrum of this species is characterized by two singlets (3H each) for the residual dmso-S and sixteen resolved aromatic resonances for the two inequivalent phen ligands (see below Figure S4). No resonances attributable to unreacted **1** were detected.

Thus, the reactivity of **1** with phen in refluxing ethanol can be summarized as reported in Scheme S3:

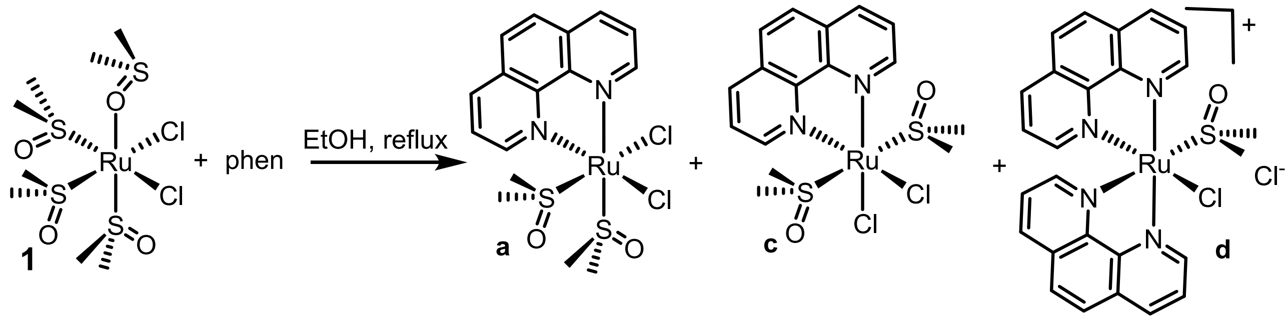

Scheme S3. The reactivity of *cis*-[$RuCl_2(dmso)_4$] (**1**) with 1 equiv. of phen in refluxing ethanol.

Very similar results were obtained, under the same conditions, using one equiv. of dppz in the place of phen.

The reaction of **1** with two equiv. of phen in refluxing ethanol (8h) afforded the same three main products **a**, **c**, and **d** as above, even though in different relative ratios. The first fraction was formed by a mixture of **a** and **c** (Figure S4), whereas the second fraction, obtained from the concentrated mother liquor, was almost pure **d** (Figure S4). To be noted that one methyl resonance of the dmso-S in **d** is shifted at very low frequencies. This singlet is attributed to the methyl that falls in the shielding cone of the adjacent phenanthrolines.

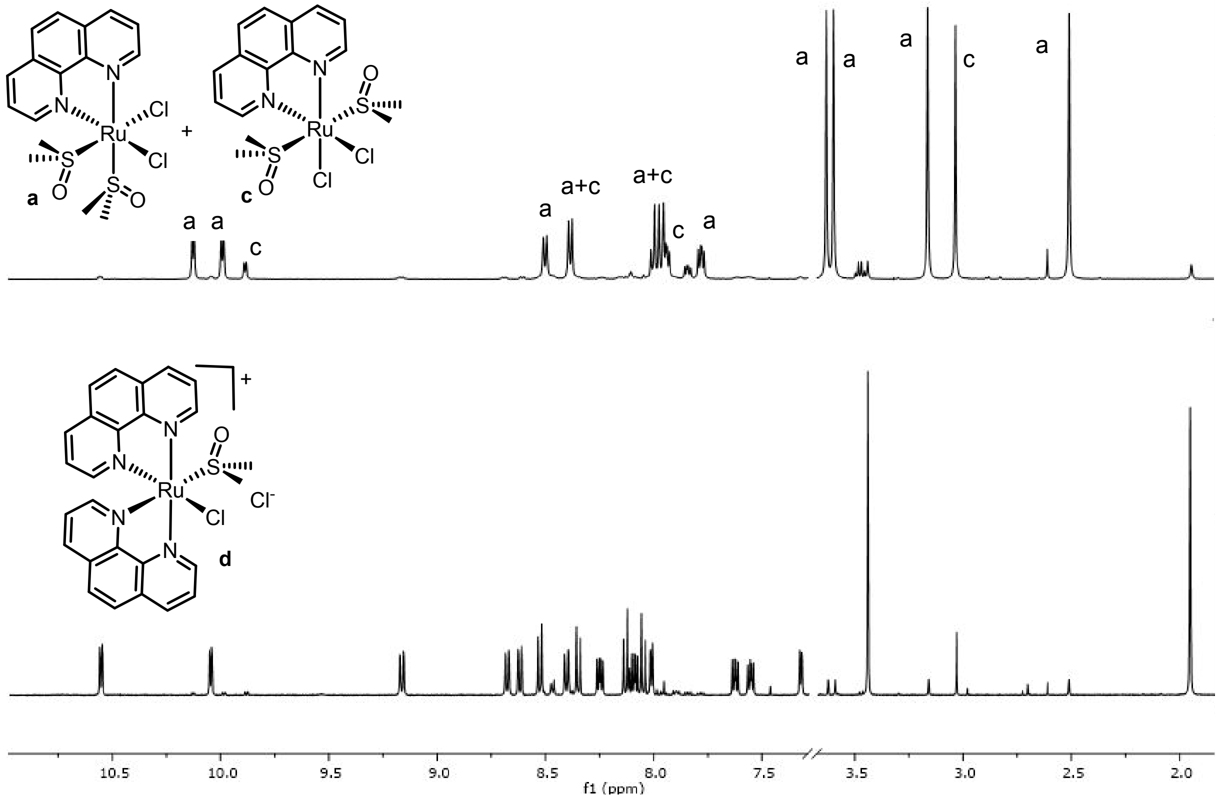

Figure S4. ^{1}H NMR spectra ($CDCl_3$) of the two fractions obtained from the reaction of **1** with two equiv. of phen in refluxing ethanol for 8h. Top: 1st fraction, which is a mixture of **a** and **c**; bottom: 2nd fraction, that consists of almost pure *cis*-[RuCl(dmso-S)$(phen)_2$]Cl (**d**).

Experimental section

***cis*-[RuCl$_2$(dmso)$_4$] (1) + 1 equiv of phen in refluxing chloroform.**

A 50.0 mg amount of *cis*-[RuCl$_2$(dmso)$_4$] (**1**) (0.10 mmol) was dissolved in 10 mL of chloroform together with one equiv. of phen (18.7 mg). The pale yellow solution was heated to reflux for 1h. The final deep-orange solution was evaporated to an oil that, upon addition of acetone (5 mL), afforded a yellow solid that was removed by filtration, washed with acetone and diethyl ether, and dried under vacuum. According to its ^{1}H NMR spectrum this precipitate is a ca. 1:1 mixture of the stereoisomers **a** and **b** plus unreacted **1**. Upon dropwise addition of diethyl ether to the concentrated mother liquor (ca. 2 mL) a pale yellow – creamy precipitate was obtained that, according to the ^{1}H NMR spectrum in $CDCl_3$, was mainly composed by unreacted **1**. The estimated yield in the **a** + **b** mixture was lower than 50%.

***trans*-[RuCl$_2$(dmso-S)$_4$] (1t) + phen in methanol.**

A 50.0 mg amount of *trans*-[RuCl$_2$(dmso-S)$_4$] (**1t**) (0.10 mmol) was dissolved in 5 mL of methanol together with one equiv. of phen (18.7 mg). The orange solution became progressively red and afforded a deep-orange precipitate formed. This was collected by filtration, washed with diethyl ether and dried under vacuum. According to its ^{1}H NMR spectrum this precipitate was pure *cis,trans*-[RuCl$_2$(dmso-S)$_2$(phen)] (**b**). Upon dropwise addition of diethyl ether to the concentrated mother liquor (ca. 2 mL) a second fraction of the same product was obtained. Total yield of **b**: 41.9 mg (80%).

***cis*-[RuCl$_2$(dmso)$_4$] (1) + 1 equiv of phen in refluxing ethanol.**

A 50.0 mg amount of *cis*-[RuCl$_2$(dmso)$_4$] (**1**) (0.10 mmol) was dissolved in 10 mL of ethanol together with one equiv. of phen (18.7 mg) and heated to reflux for 2h. The final red-orange solution slowly afforded (72 h) at room temperature an orange precipitate, that was collected by filtration, washed with cold ethanol and diethyl ether, and dried under vacuum. According to its ^{1}H NMR spectrum this precipitate is a ca. 6:1 mixture of the stereoisomers **a** and **c**. Yield 25.3 mg (48%). Upon dropwise addition of diethyl ether to the concentrated mother liquor (ca. 4 mL) a second orange precipitate was obtained that, according to the ^{1}H NMR spectrum in $CDCl_3$, contained comparable amounts of **a**, **c**, and *cis*-[RuCl(dmso-S)(phen)$_2$]Cl (**d**).

***cis*-[RuCl$_2$(dmso)$_4$] (1) + 2 equiv of phen in refluxing ethanol.**

A 101.6 mg amount of *cis*-[RuCl$_2$(dmso)$_4$] (**1**) (0.21 mmol) was dissolved in 15 mL of ethanol together with two equiv. of phen (74.8 mg) and heated to reflux for 8h. The final deep-red solution was concentrated to ca. half volume. Dropwise addition of diethyl ether until cloudiness afforded an orange precipitate, that was collected by filtration, washed with cold ethanol and diethyl ether,

and dried under vacuum. According to its ^{1}H NMR spectrum this precipitate was a ca. 5:1 mixture of the stereoisomers **a** and **c**. Yield 62.45 mg (59%). A small amount of a second fraction precipitated spontaneously from the mother liquor (containing also the diethyl ether from the washing). According to its ^{1}H NMR spectrum it was almost pure *cis*-[RuCl(dmso-S)(phen)$_2$]Cl (**d**).

NMR characterization of compounds a - d

cis,cis-[RuCl$_2$(dmso-S)$_2$(phen)] (**a**) ($C_{16}H_{20}Cl_2N_2O_2RuS_2$, MW = 507.94). ^{1}H NMR (CDCl$_3$) δ, ppm: 2.52, 3.17, 3.60, 3.63 (s, 3H, dmso-S), 7.79 (dd, 1H, H8), 7.98 (m, 3H, H3+H5+H6), 8.39 (dd, 1H, H4), 8.51 (dd, 1H, H7), 9.99 (dd, 1H, H2), 10.14 (dd, 1H, H9).

trans,cis-[RuCl$_2$(dmso-S)$_2$(phen)] (**b**) ($C_{16}H_{20}Cl_2N_2O_2RuS_2$, MW = 507.94). ^{1}H NMR (CDCl$_3$) δ, ppm: 3.66 (s, 12H, dmso-S), 7.86 (m, 2H, H3,8), 7.99 (s, 2H, H5,6), 8.44 (dd, 2H, H4,7), 10.07 (dd, 2H, H2,9).

cis,trans-[RuCl$_2$(dmso-S)$_2$(phen)] (**c**) ($C_{16}H_{20}Cl_2N_2O_2RuS_2$, MW = 507.94). ^{1}H NMR (CDCl$_3$) δ, ppm: 3.04 (s, 12H, dmso-S), 7.84 (dd, 2H, H3,8), 8.01(s, 2H, H5,6), 8.37 (dd, 2H, H4,7), 9.89 (dd, 2H, H2,9).

cis-[RuCl(dmso-S)(phen)$_2$][Cl] (**d**) ($C_{26}H_{22}Cl_2N_4ORuS$, MW = 610.47). ^{1}H NMR (CDCl$_3$) δ, ppm: 1.96 (s, 3H), 3.45 (s, 3H), 7.33 (dd, 1H), 7.56 (dd, 1H), 7.63 (dd, 1H), 8.02 (dd, 1H), 8.05 (dd, 1H), 8.09 (dd, 1H), 8.14 (dd, 1H), 8.25 (dd, 1H), 8.35 (dd, 1H), 8.41 (dd, 1H), 8.53 (dd, 1H), 8.62 (dd, 1H), 8.68 (dd, 1H), 9.17 (dd, 1H), 10.05 (dd, 1H), 10.56 (dd, 1H).

Spectral characterization of the new *cis*-locked precursors 6 and 7

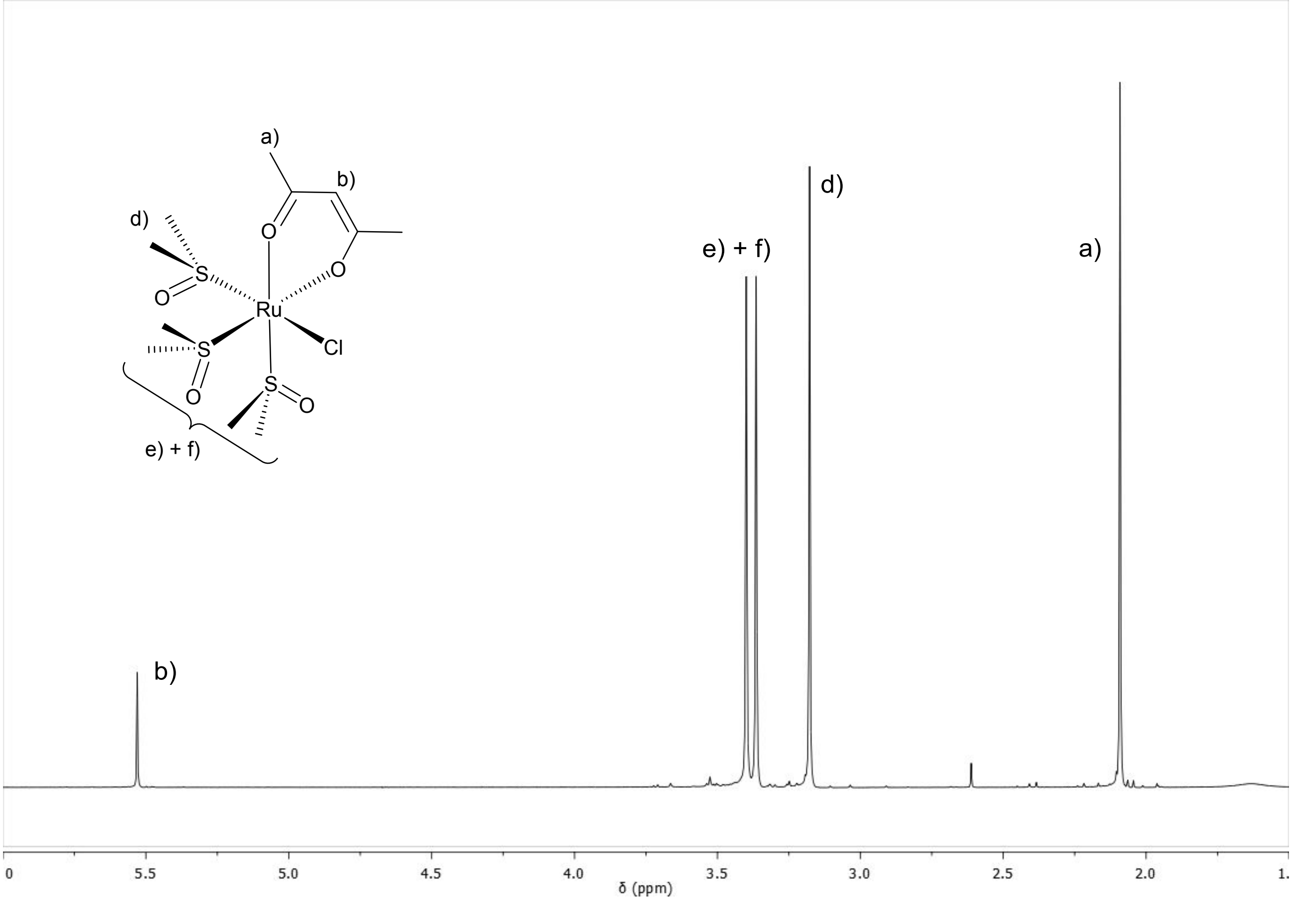

Figure S5. ^{1}H NMR spectrum ($CDCl_3$) of *fac*-[RuCl(dmso-S)$_3$(η^2-acac)] (**6**).

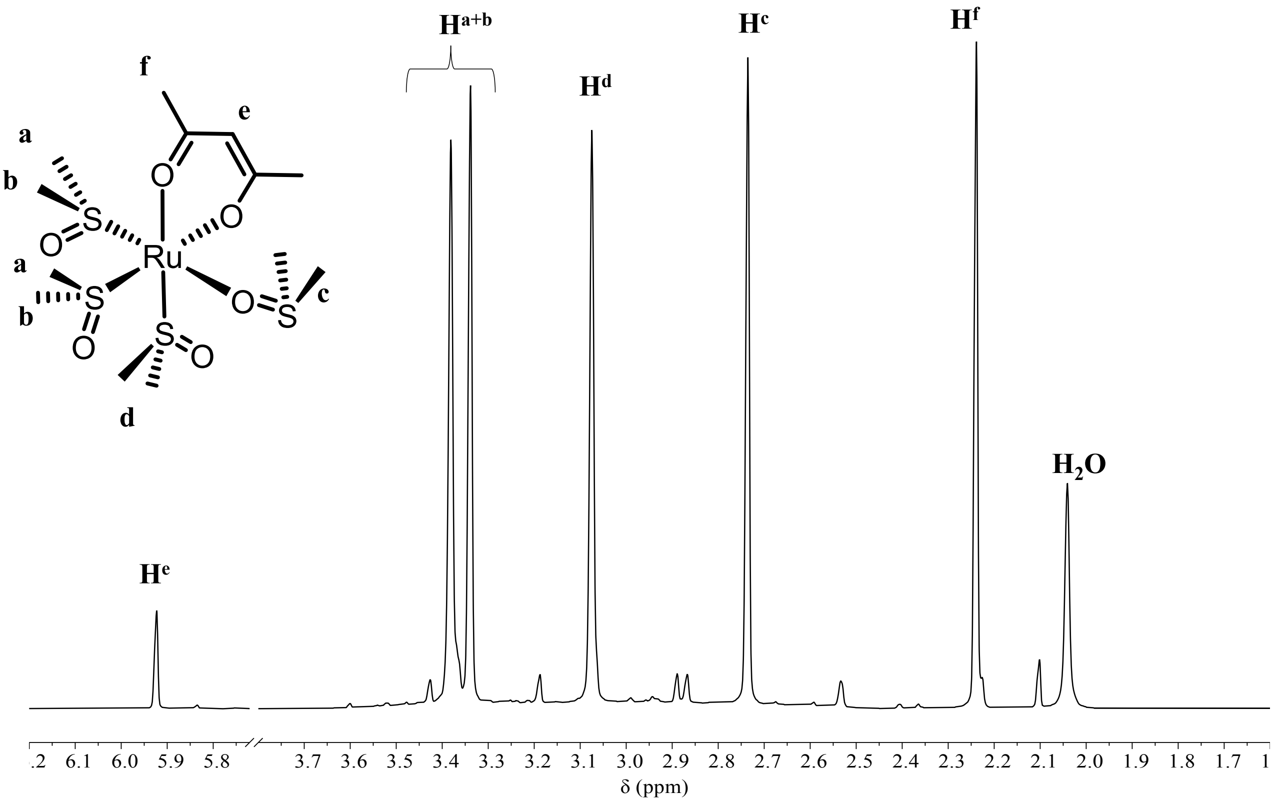

Figure S6. ^{1}H NMR spectrum (CD_3NO_2) of *fac*-[Ru(dmso-O)(dmso-S)$_3$(η^2-acac)] (**7**).

Reactions of the *cis*-locked precursors with bpy

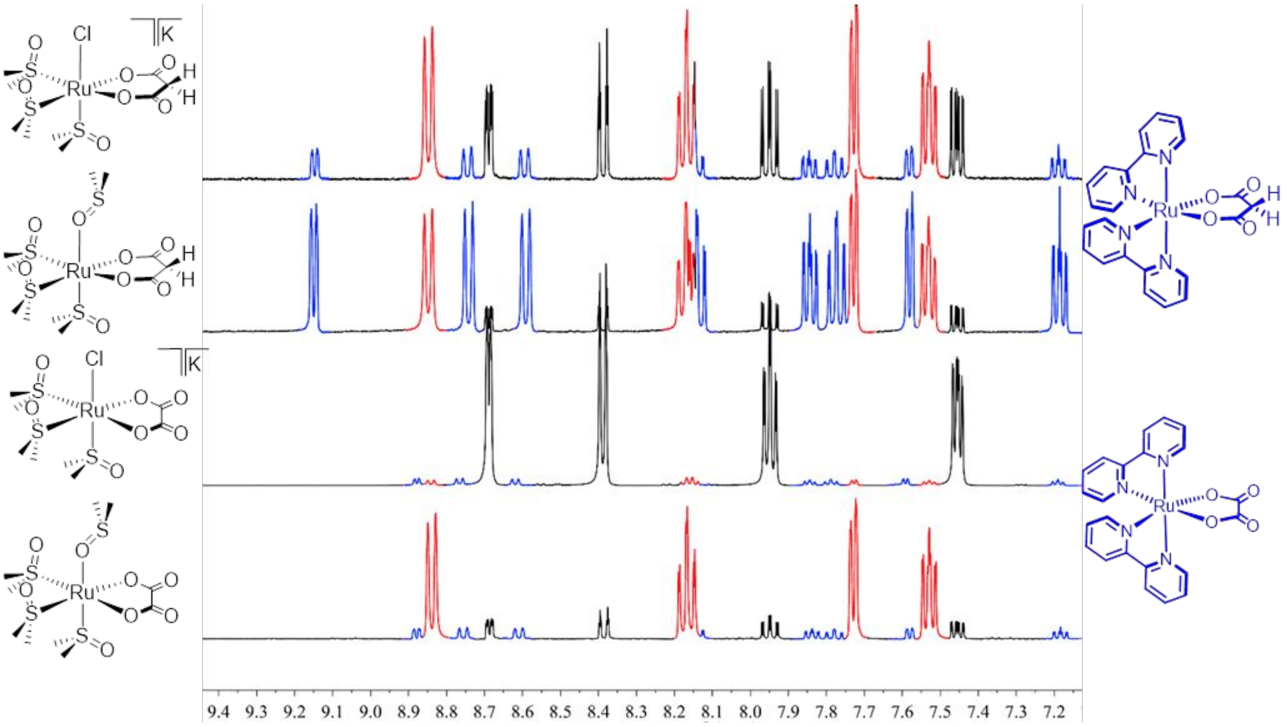

Figure S7. ^{1}H NMR spectra (aromatic region) in DMSO-d_6 of the raw product mixtures from the reactions between the *cis*-locked precursors **2** – **5** and bpy performed in a MW reactor. Color code: black = unreacted bpy, blue = the bis-bpy product, red = $[Ru(bpy)_3]^{2+}$. Reaction conditions: [Ru] = 390 mM, absolute ethanol, bpy/Ru = 2, MW, 120°C, 1h.

Table S1. Molar percentages determined by NMR integration in the raw product mixtures from the reactions between the *cis*-locked precursors **2** – **7** and bpy performed in a MW reactor. Reaction conditions: [Ru] = 390 mM, absolute ethanol, bpy/Ru = 2, 120°C, 1h.

**Complex**	**Free bpy%**	$[Ru(bpy)_2(O–O)]^{n+}$	$[Ru(bpy)_3]^{2+}$
K[*fac*-RuCl(dmso-S)$_3$(η^2-mal)] (**2**)	34	18	48
[*fac*-Ru(dmso-O)(dmso-S)$_3$(η^2-mal)] (**3**)	10	64	26
K[*fac*-RuCl(dmso-S)$_3$(η^2-ox)] (**4**)	97	2	1
[*fac*-Ru(dmso-O)(dmso-S)$_3$(η^2-ox)] (**5**)	24	14	62
[*fac*-RuCl(dmso-S)$_3$(η^2-acac)] (**6**)	46	38	15
[*fac*-Ru(dmso-O)(dmso-S)$_3$(η^2-acac)][PF_6] (**7**)	n.d.*	73	27

* In this case a precipitate is found in the vial at the end of the run, a mixture of [Ru(bpy)$_2$(η^2-acac)][PF_6] (**8**) and [Ru(bpy)$_3$][PF_6]$_2$.

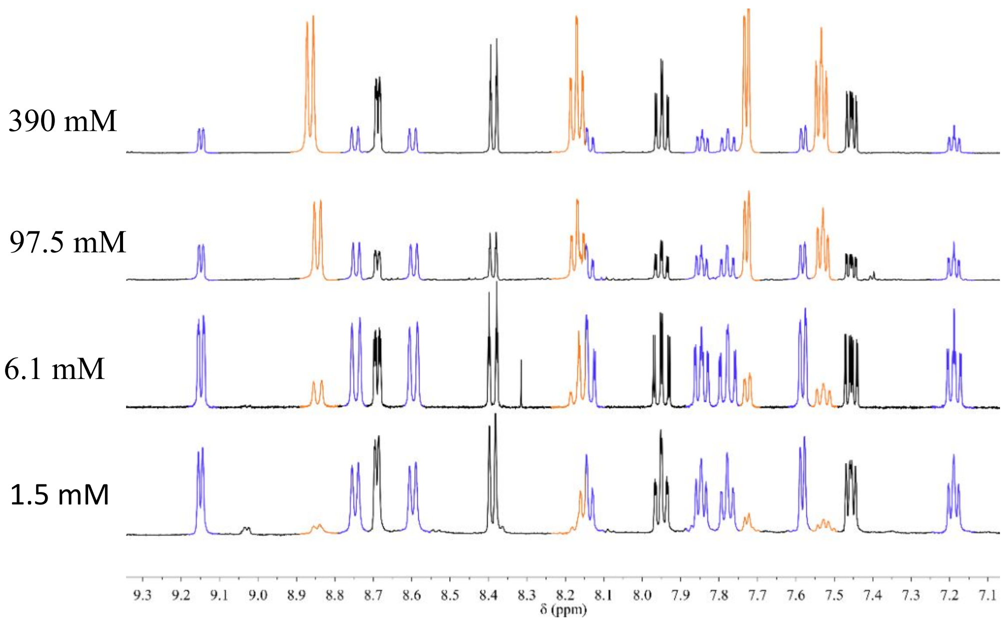

Figure S8. The ^{1}H NMR spectra (aromatic region) in DMSO-d_6 show the effect of the concentration on the selectivity towards [Ru(bpy)$_2$(η^2-mal)] in the reaction between K[*fac*-RuCl(dmso-S)$_3$(η^2-mal)] (**2**) and bpy performed in a MW reactor. Color code: black = unreacted bpy, blue = [Ru(bpy)$_2$(η^2-mal)], red-orange = [Ru(bpy)$_3$]$^{2+}$. Reaction conditions: absolute ethanol, bpy/Ru = 2, 120°C, 1h.

Table S2. The effect of the concentration on the selectivity towards [Ru(bpy)$_2$(η^2-mal)] in the reaction between K[*fac*-RuCl(dmso-S)$_3$(η^2-mal)] (**2**) and bpy performed in a MW reactor. The % values are obtained by integrating the NMR spectra of Figure S8. Reaction conditions: absolute ethanol, bpy/Ru = 2, 120°C, 1h.

[Ru] mM	Free bpy	[Ru(bpy)$_2$(η^2-mal)]	[Ru(bpy)$_3$]$^{2+}$
390	34	18	48
97.5	32	38	30
6.1	43	51	6
1.5	59	51	3

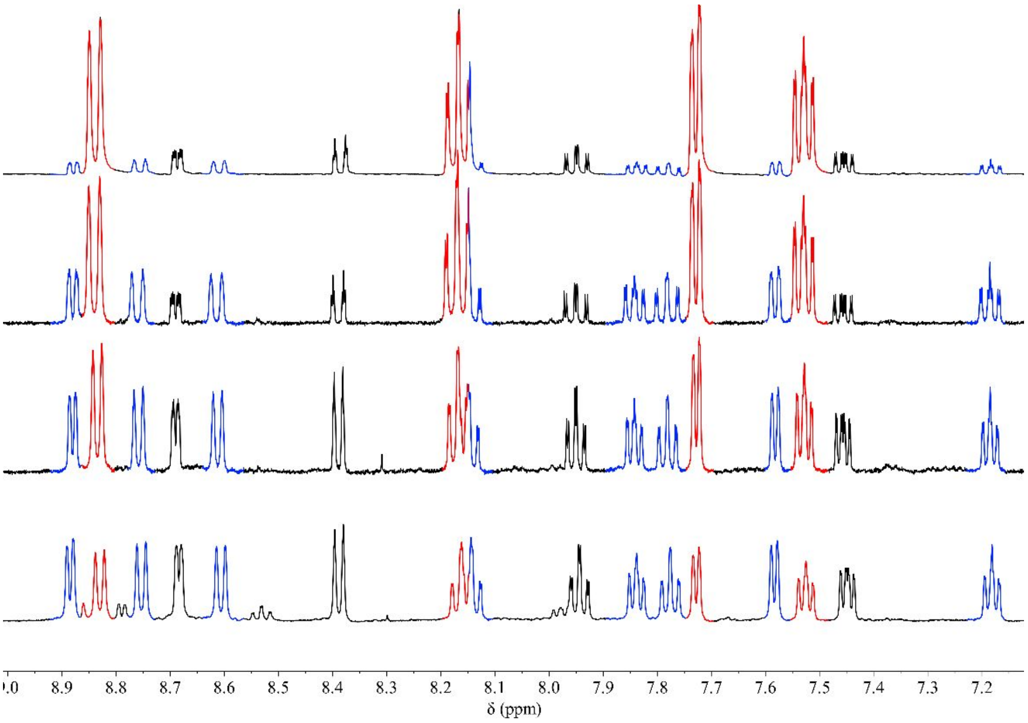

Figure S9. The ^{1}H NMR spectra (aromatic region) in DMSO-d_6 show the effect of the concentration on the selectivity towards [Ru(bpy)$_2$(η^2-ox)] in the reaction between [*fac*-Ru(dmso-O)(dmso-S)$_3$(η^2-ox)] (**5**) and bpy performed in a MW reactor. Color code: black = unreacted bpy, blue = [Ru(bpy)$_2$(η^2-ox)], red-orange = [Ru(bpy)$_3$]$^{2+}$. Reaction conditions: absolute ethanol, bpy/Ru = 2, 120°C, 1h.

Table S3. The effect of the concentration on the selectivity towards [Ru(bpy)$_2$(η^2-ox)] in the reaction between [*fac*-Ru(dmso-O)(dmso-S)$_3$(η^2-ox)] (**5**) and bpy performed in a MW reactor. The % values are obtained by integrating the NMR spectra of Figure S9. Reaction conditions: absolute ethanol, bpy/Ru = 2, 120°C, 1h.

[Ru] mM	Free bpy	[Ru(bpy)$_2$(η^2-ox)]	[Ru(bpy)$_3$]$^{2+}$
390	26	13	62
97.5	22	41	37
24.3	36	42	22
6.1	43	44	13

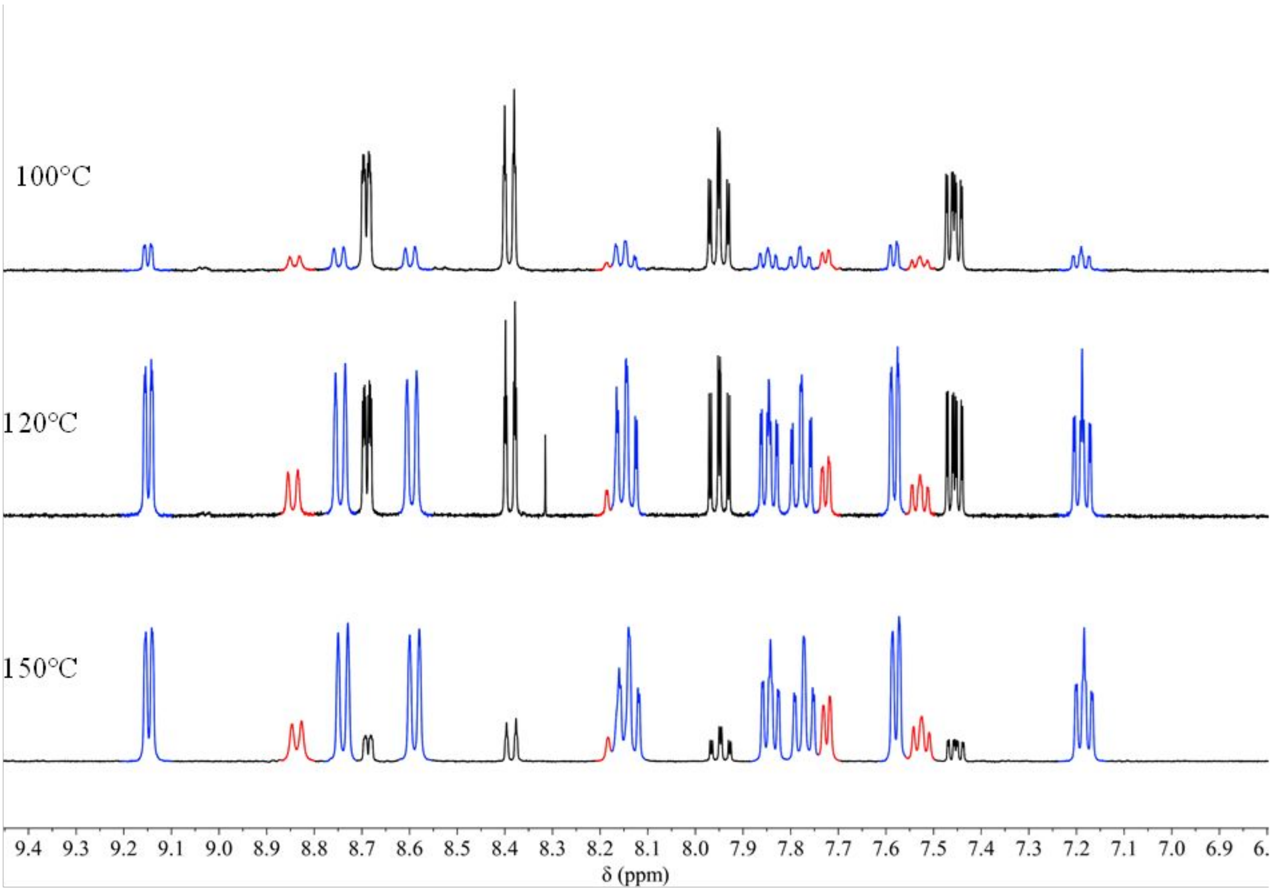

Figure S10. The ^{1}H NMR spectra (aromatic region) in DMSO-d_6 show the effect of the temperature on the selectivity towards [Ru(bpy)$_2$(η^2-mal)] in the reaction between K[*fac*-RuCl(dmso-S)$_3$(η^2-mal)] (**2**) and bpy performed in a MW reactor. Color code: black = unreacted bpy, blue = [Ru(bpy)$_2$(η^2-mal)], red-orange = [Ru(bpy)$_3$]$^{2+}$. Reaction conditions: absolute ethanol, [Ru] = 6.1 mM, bpy/Ru = 2, 1h.

Table S4. The effect of the temperature on the selectivity towards [Ru(bpy)$_2$(η^2-mal)] in the reaction between K[*fac*-RuCl(dmso-S)$_3$(η^2-mal)] (**2**) and bpy performed in a MW reactor. The % values are obtained by integrating the NMR spectra of Figure S10. Reaction conditions: absolute ethanol, [Ru] = 6.1 mM, bpy/Ru = 2, 1h.

T (°C)	Free bpy	[Ru(bpy)$_2$(η^2-mal)]	[Ru(bpy)$_3$]$^{2+}$
100	26	13	62
120	43	51	6
150	16	77	7

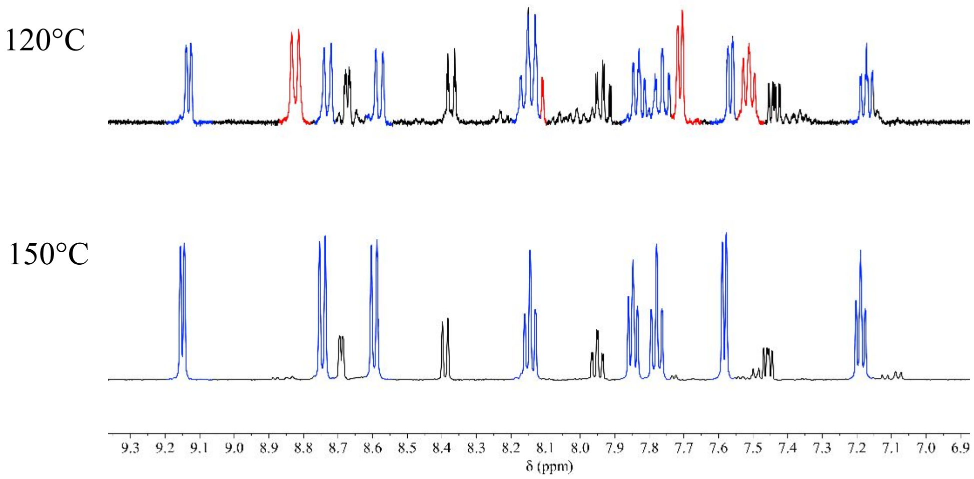

Figure S11. The ^{1}H NMR spectra (aromatic region) in DMSO-d_6 show the effect of the temperature on the selectivity towards [Ru(bpy)$_2$(η^2-mal)] in the reaction between [*fac*-Ru(dmso-O)(dmso-S)$_3$(η^2-mal)] (**3**) and bpy performed in a MW reactor. Color code: black = unreacted bpy, blue = [Ru(bpy)$_2$(η^2-mal)], red-orange = [Ru(bpy)$_3$]$^{2+}$. Reaction conditions: absolute ethanol, [Ru] = 6.1 mM, bpy/Ru = 2, , 1h.

Table S5. The effect of the temperature on the selectivity towards [Ru(bpy)$_2$(η^2-mal)] in the reaction between [*fac*-Ru(dmso-O)(dmso-S)$_3$(η^2-mal)] (**3**) and bpy performed in a MW reactor. The % values are obtained by integrating the NMR spectra of Figure S11. Reaction conditions: absolute ethanol, [Ru] = 6.1 mM, bpy/Ru = 2, 1h.

T (°C)	Free bpy	[Ru(bpy)$_2$(η^2-mal)]	[Ru(bpy)$_3$]$^{2+}$
120	27	46	26
150	11	89	<1%

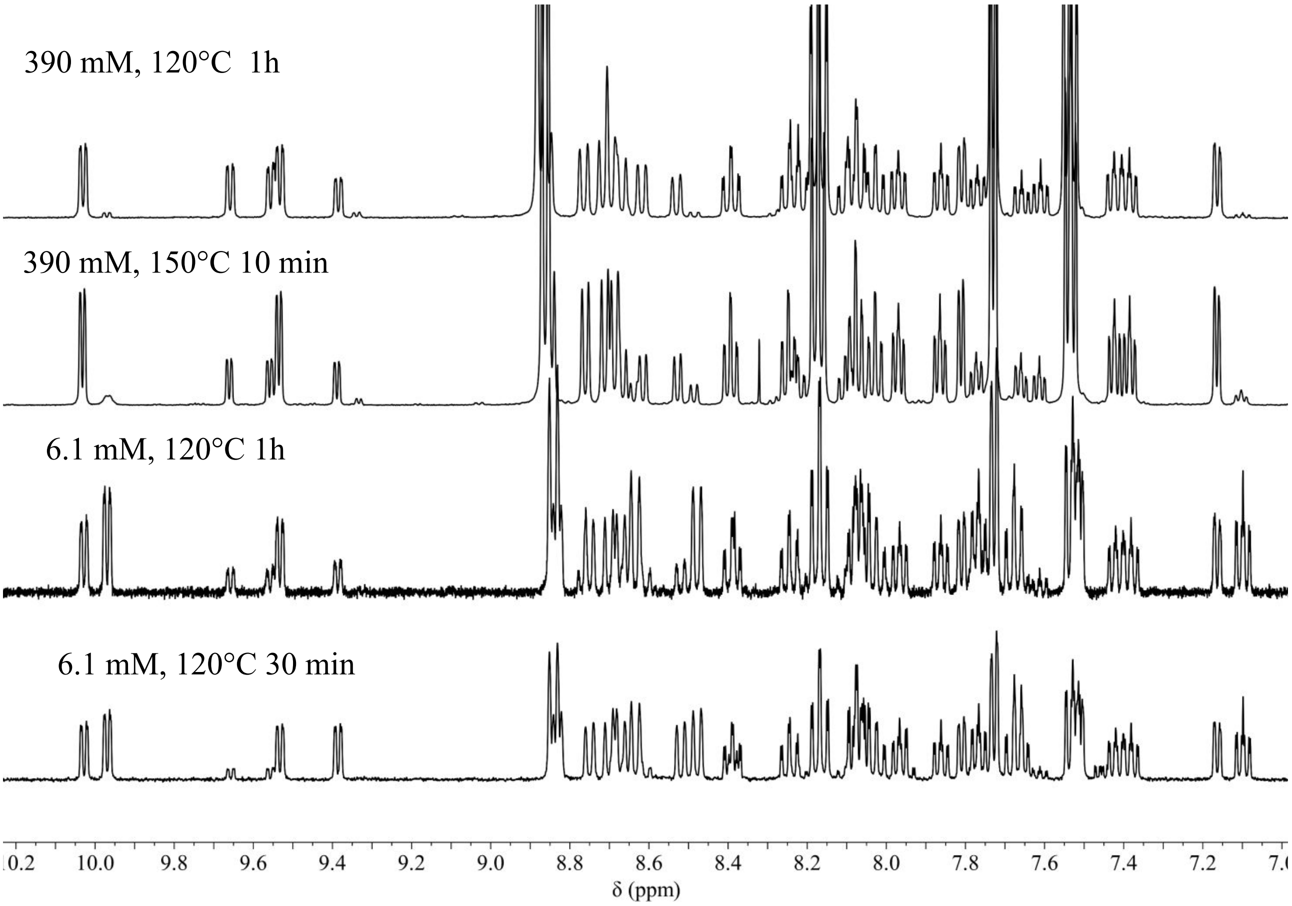

Figure S12. The ^{1}H NMR spectra (aromatic region) in DMSO-d_6 show the effect of the concentration, temperature, and reaction time on the outcome of the reaction between [*cis*-$RuCl_2(dmso)_4$] (**1**) and bpy performed in a MW reactor. See Figure S13 for peak assignments. Reaction conditions: absolute ethanol, bpy/Ru = 2, 1h.

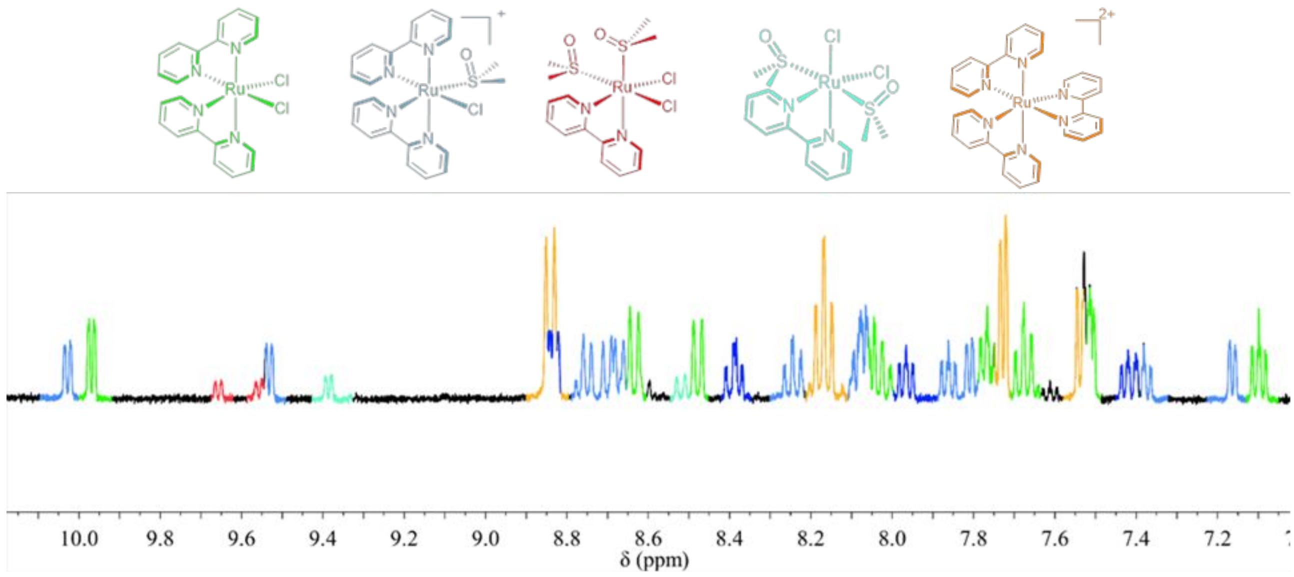

Figure S13. Peak assignments in the ^{1}H NMR spectrum (aromatic region) in DMSO-d_6 of the raw reaction mixture from the reaction between [*cis*-$RuCl_2(dmso)_4$] (**1**) and bpy performed in a MW reactor. Reaction conditions: absolute ethanol, [Ru] = 6.1 mM, T = 120°C, bpy/Ru = 2, 1h.

Spectral characterization of compounds 8 – 15

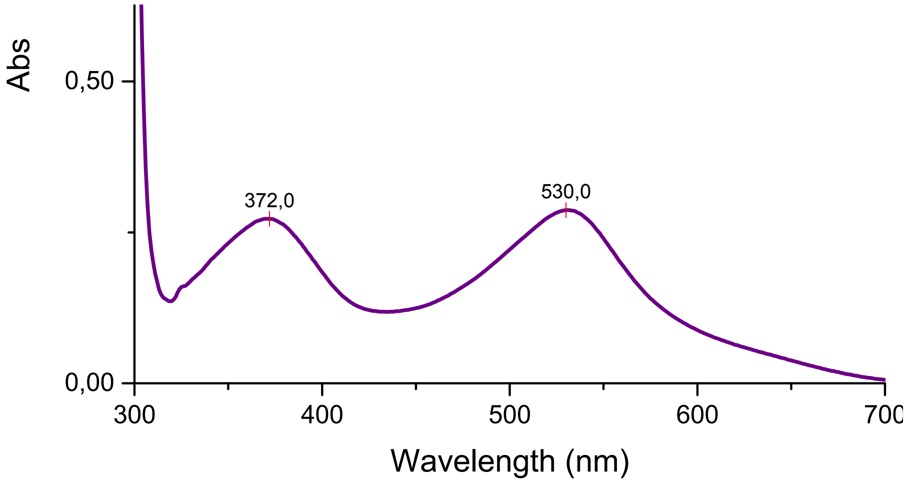

Figure S14. UV-vis spectrum in chloroform of $[Ru(bpy)_2(\eta^2\text{-acac})][PF_6]$ (**8**).

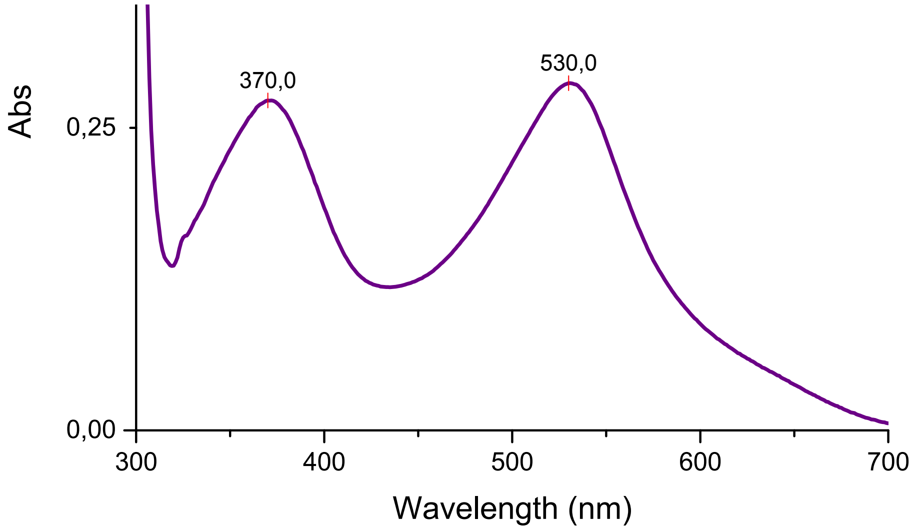

Figure S15. UV-vis spectrum in chloroform of $[Ru(bpy)_2(\eta^2\text{-mal})]$ (**9**).

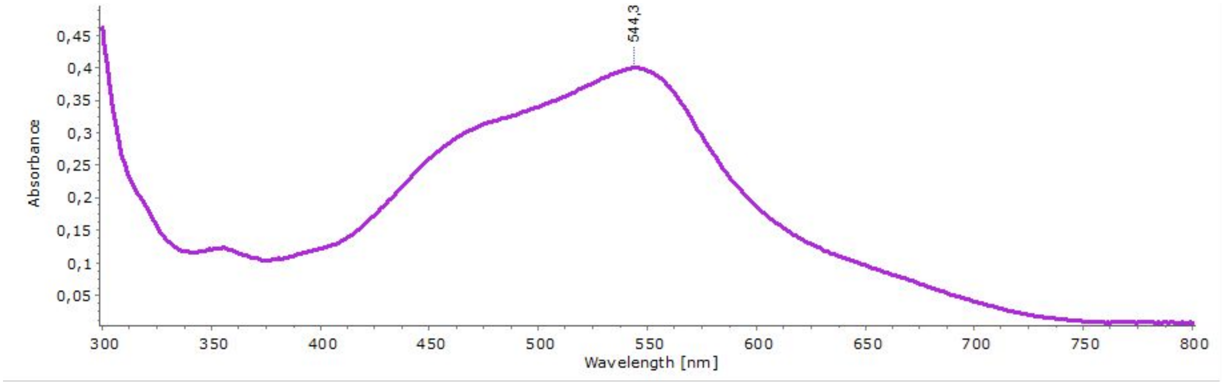

Figure S16. UV-vis spectrum in $CHCl_3$ of $[Ru(phen)_2(\eta^2\text{-mal})]$ (**10**).

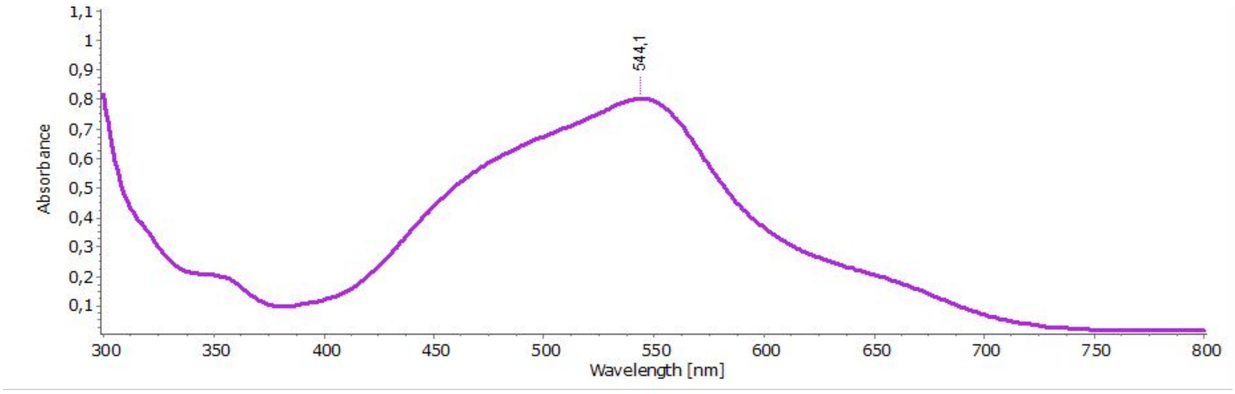

Figure S17. UV-vis spectrum in $CHCl_3$ of [Ru(phen)$_2$(η^2-ox)] (**12**).

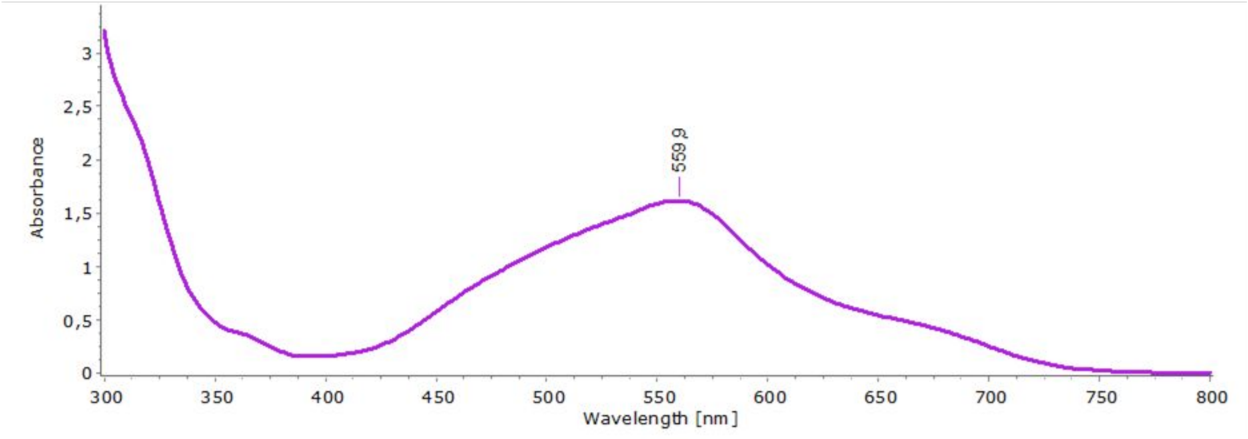

Figure S18. UV-vis spectrum in MeOH of [Ru(dpphen)$_2$(η^2-ox)] (**13**).

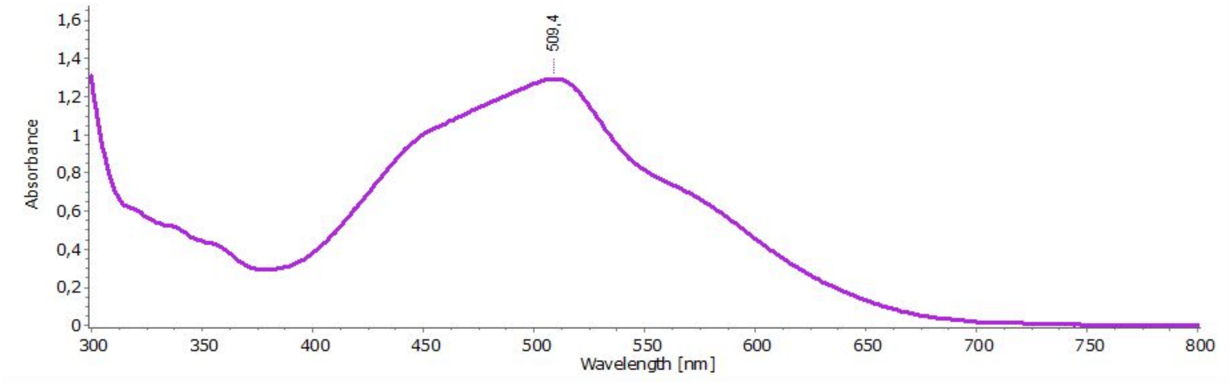

Figure S19. UV-vis spectrum in $CHCl_3$ of [Ru(phen)$_2$(η^2-acac)]Cl (**14**).

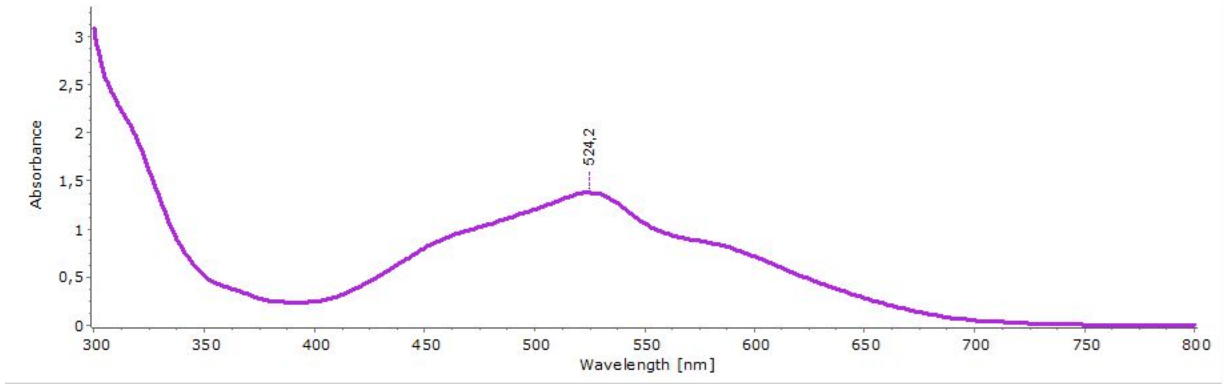

Figure S20. UV-vis spectrum in $CHCl_3$ of [Ru(dpphen)$_2$(η^2-acac)]Cl (**15**).

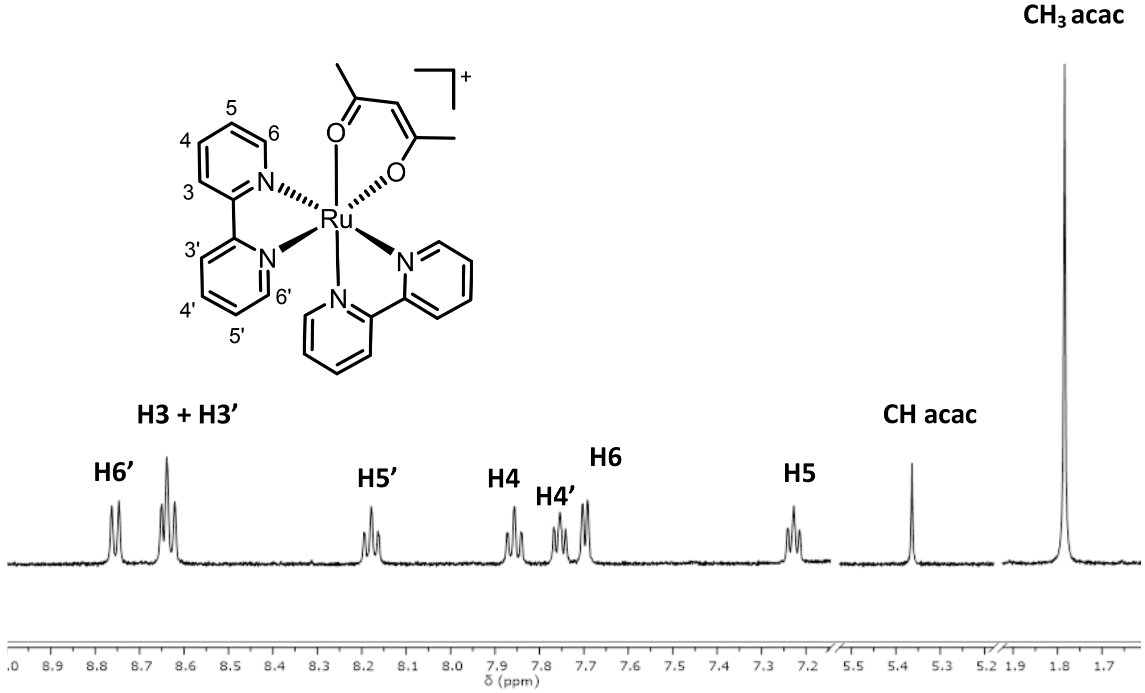

Figure S21. ^{1}H NMR spectrum (DMSO-d_6) of [Ru(bpy)$_2$(η^2-acac)][PF$_6$] (**8**) with labeling scheme.

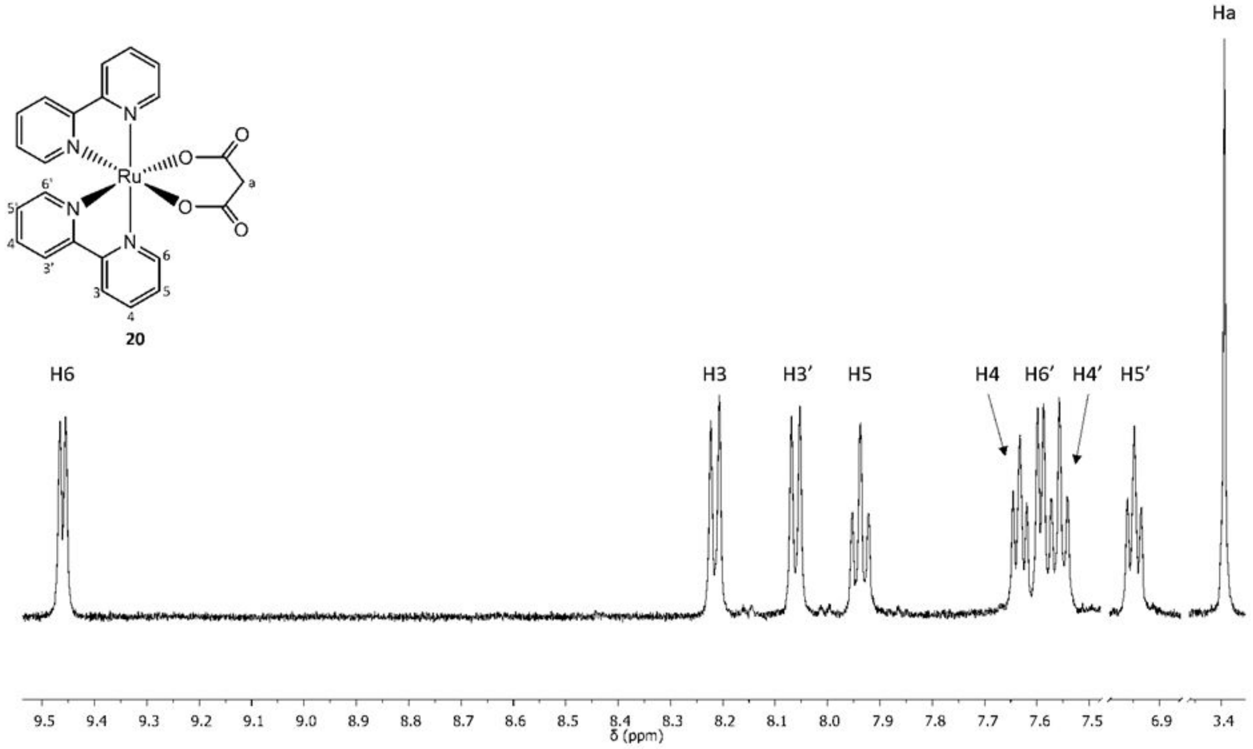

Figure S22. ^{1}H NMR spectrum (DMSO-d_6) of [Ru(bpy)$_2$(η^2-mal)] (**9**) with labeling scheme.

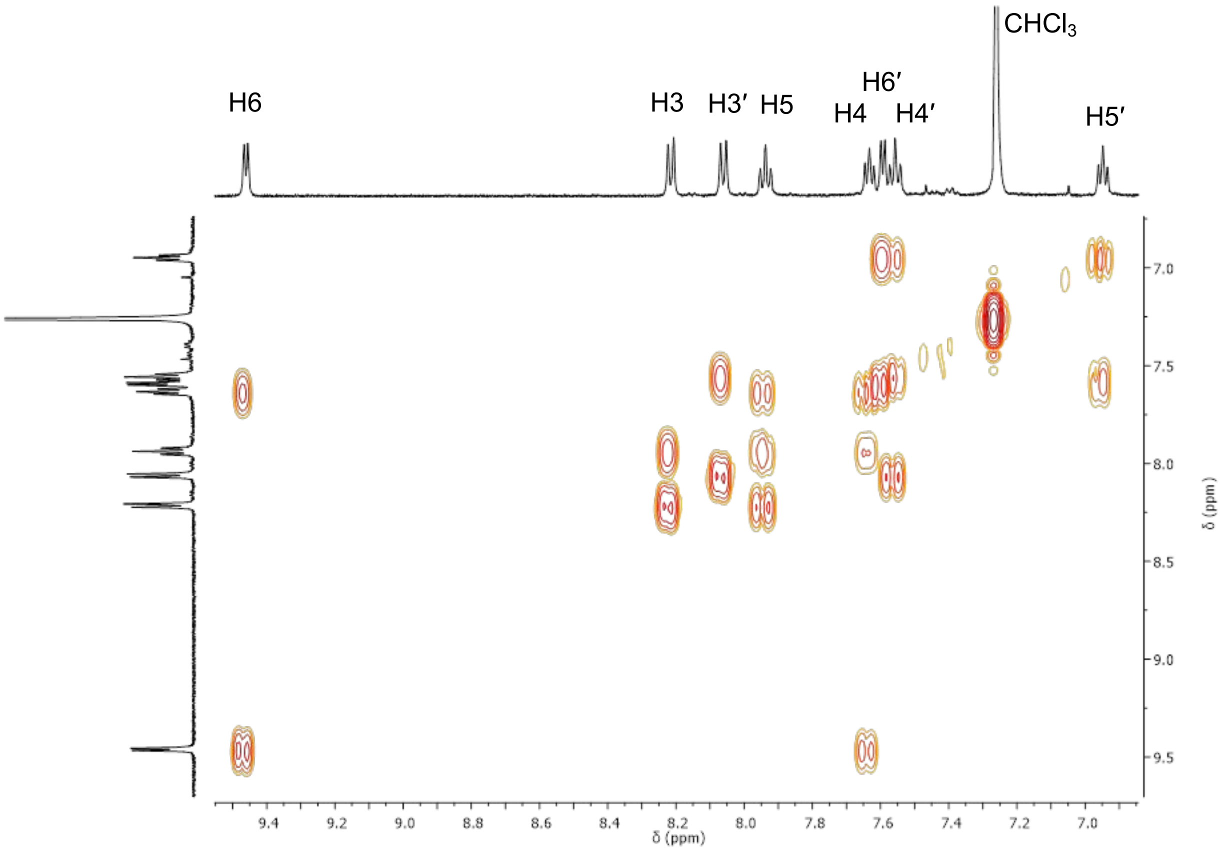

Figure S23. Downfield region of the H-H COSY spectrum ($CDCl_3$) of [Ru(bpy)$_2$(η^2-mal)] (**9**). See Figure S22 for the labeling scheme.

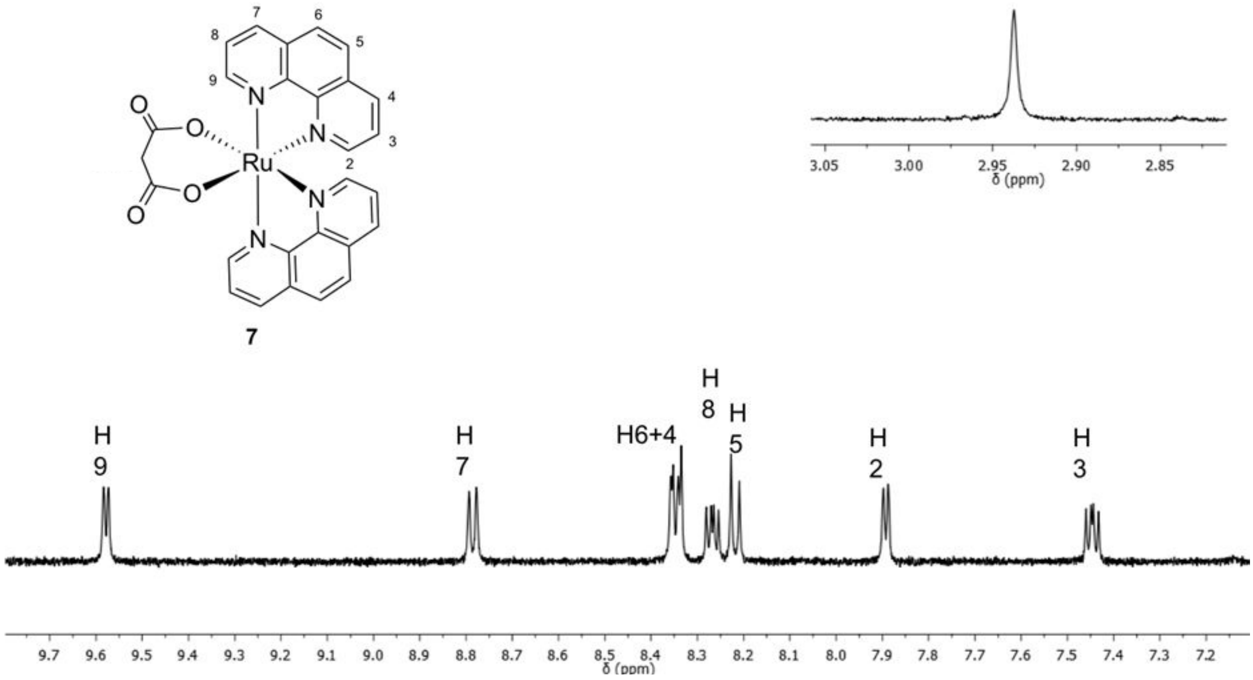

Figure S24. ^{1}H NMR spectrum (DMSO-d_6) of [Ru(phen)$_2$(η^2-mal)] (**10**) with labeling scheme. In the insert the resonance of malonate.

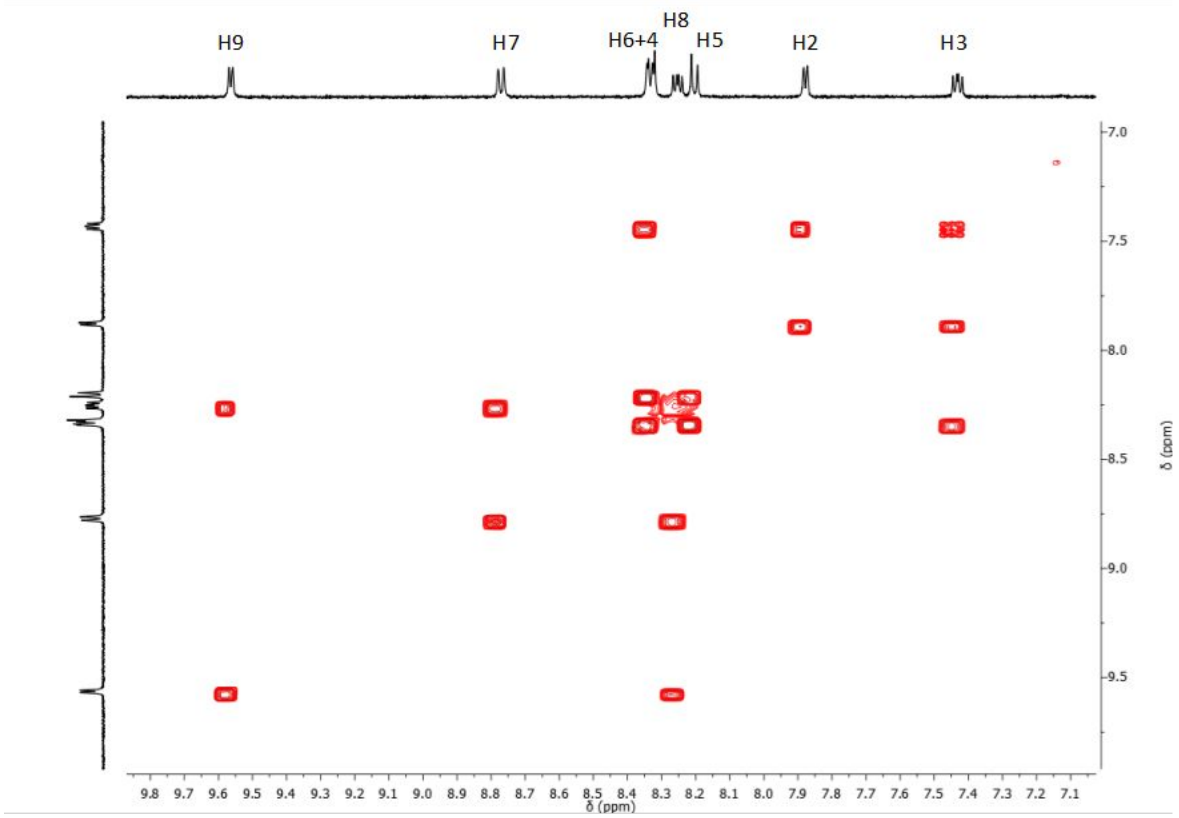

Figure S25. Downfield region of the H-H COSY spectrum (DMSO-d_6) of [Ru(phen)$_2$(η^2-mal)] (**10**); see Figure S24 for the labeling scheme.

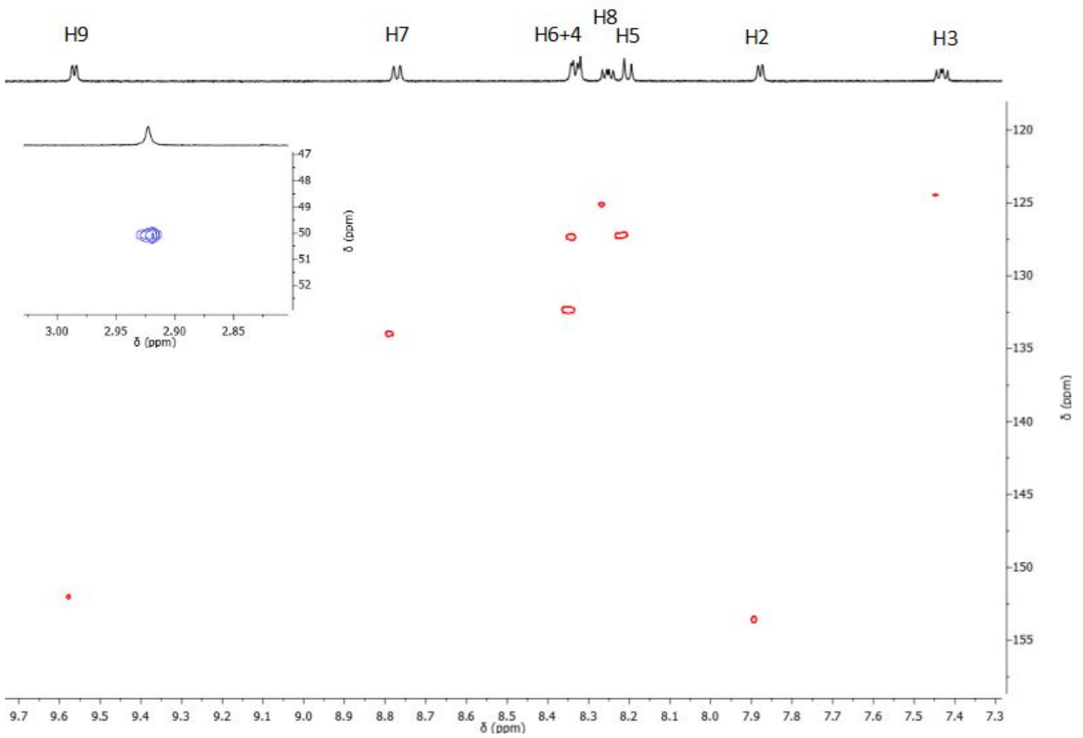

Figure S26. Downfield region of the HSQC spectrum (DMSO-d_6) of [Ru(phen)$_2$(η^2-mal)] (**10**); see Figure S24 for the labeling scheme.

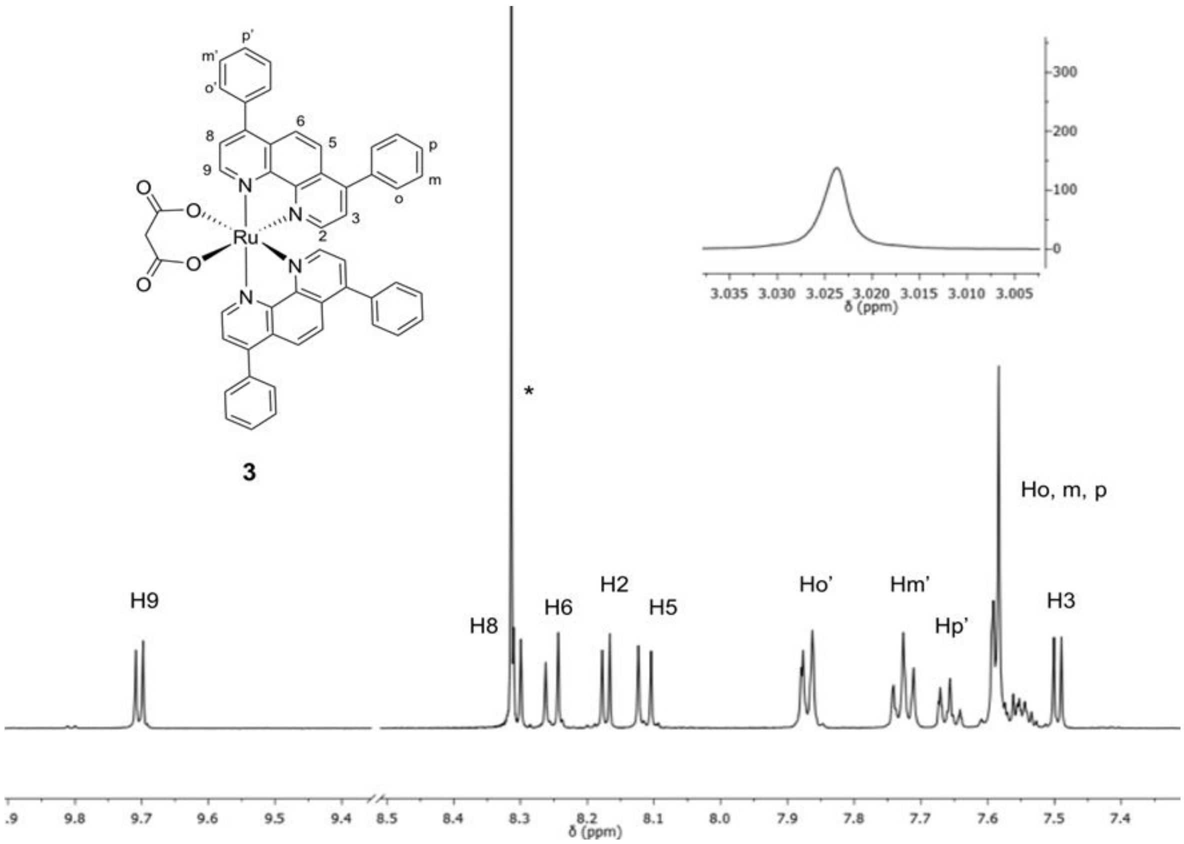

Figure S27. ^{1}H NMR spectrum (DMSO-d_6) of [Ru(dpphen)$_2$(η^2-mal)] (**11**) with labeling scheme. In the insert the resonance of malonate. The peak of residual chloroform is marked with an *.

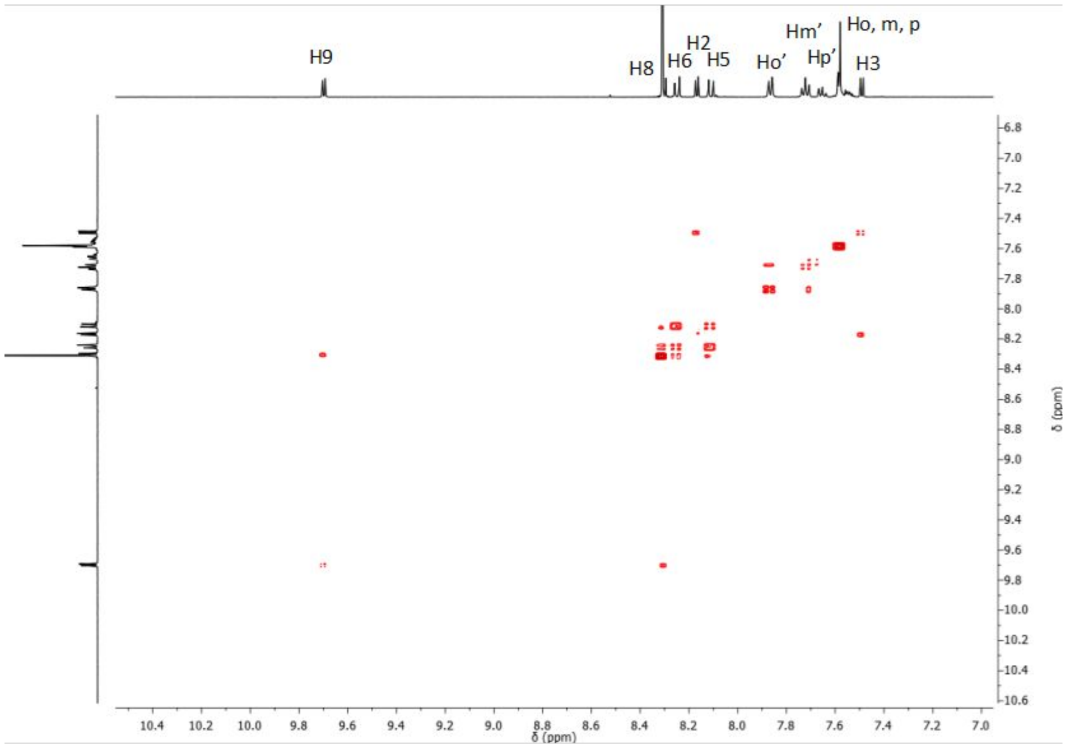

Figure S28. Downfield region of the H-H COSY spectrum (DMSO-d_6) of [Ru(dpphen)$_2$(η^2-mal)] (**11**) see Figure S27 for the labeling scheme.

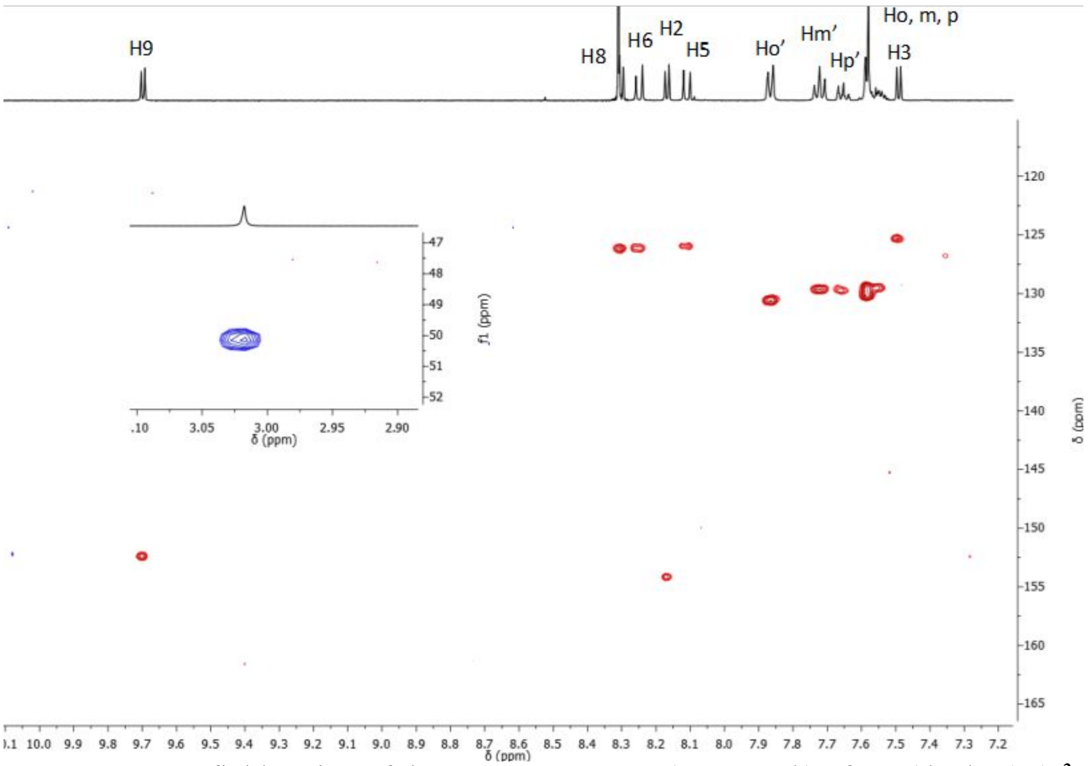

Figure S29. Downfield region of the HSQC spectrum (DMSO-d_6) of [Ru(dpphen)$_2$(η^2-mal)] (**11**) see Figure S27 for the labeling scheme.

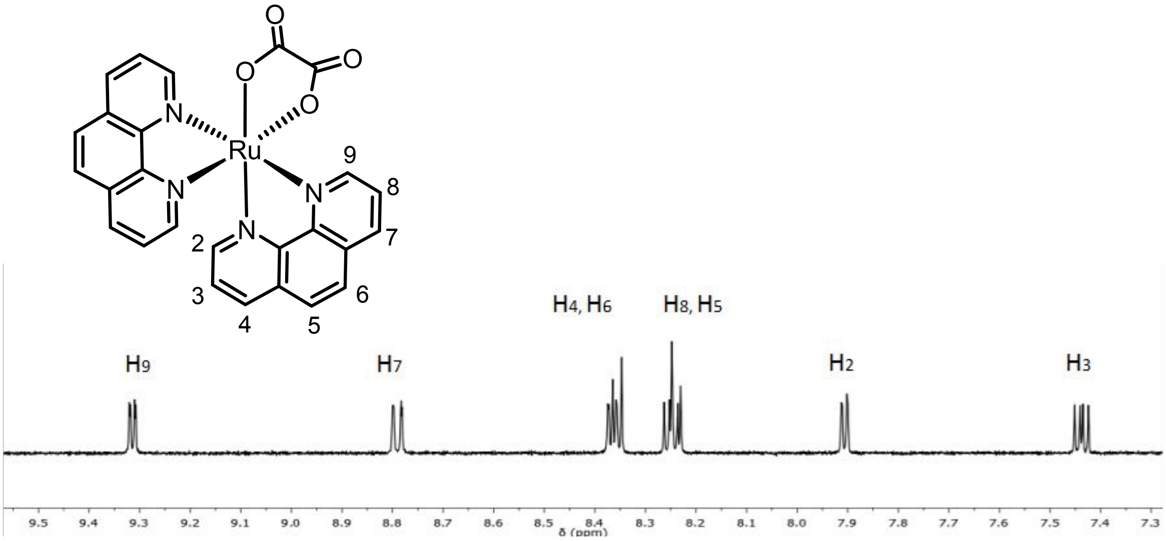

Figure S30. ^{1}H NMR spectrum (DMSO-d_6) of [Ru(phen)$_2$(η^2-ox)] (**12**) with labeling scheme.

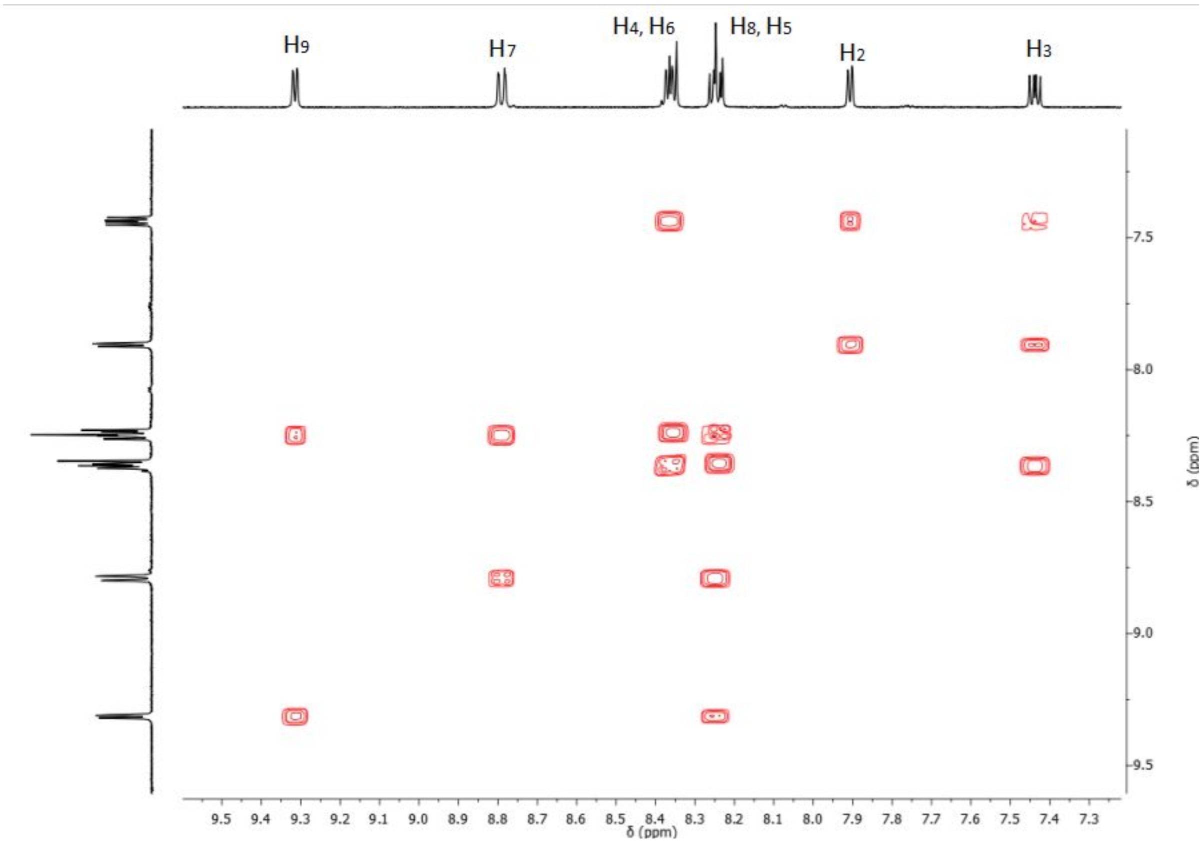

Figure S31. H-H COSY spectrum (DMSO-d_6) of [Ru(phen)$_2$(η^2-ox)] (**12**); see Figure S30 for the labeling scheme.

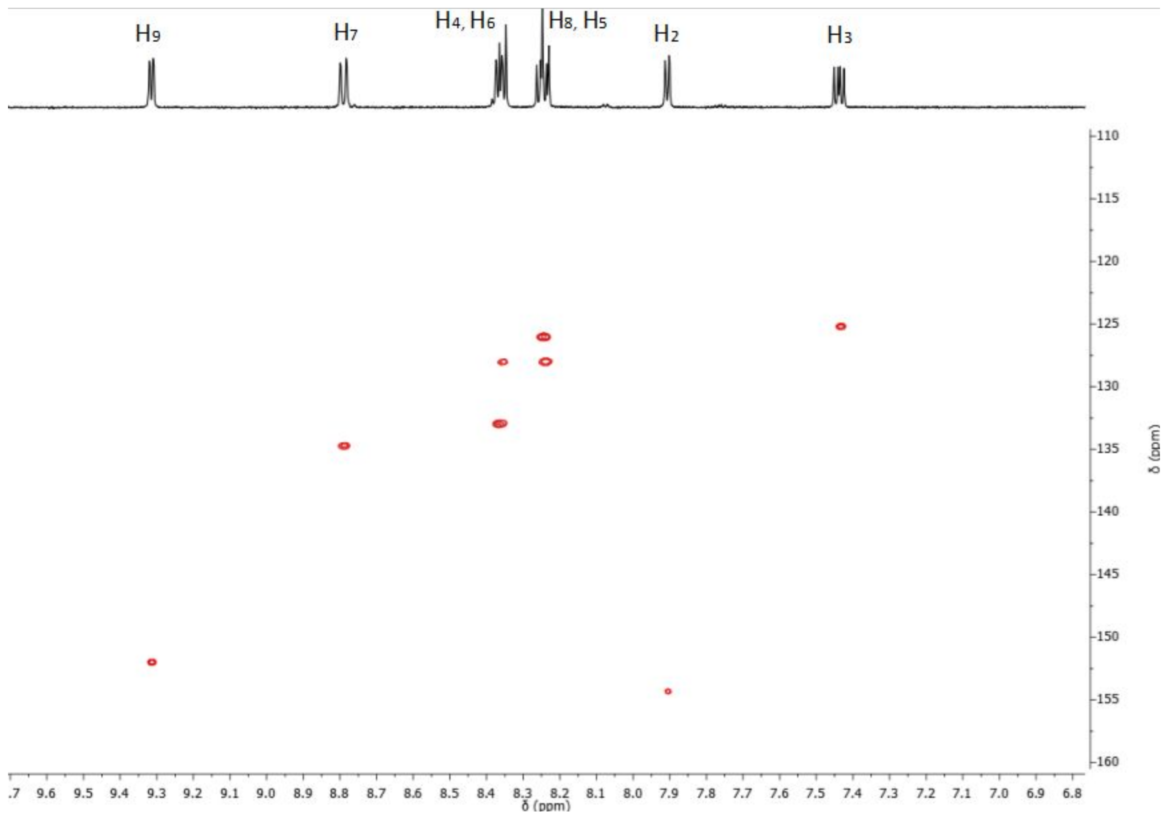

Figure S32. HSQC spectrum (DMSO-d_6) of [Ru(phen)$_2$(η^2-ox)] (**12**); see Figure S30 for the labeling scheme.

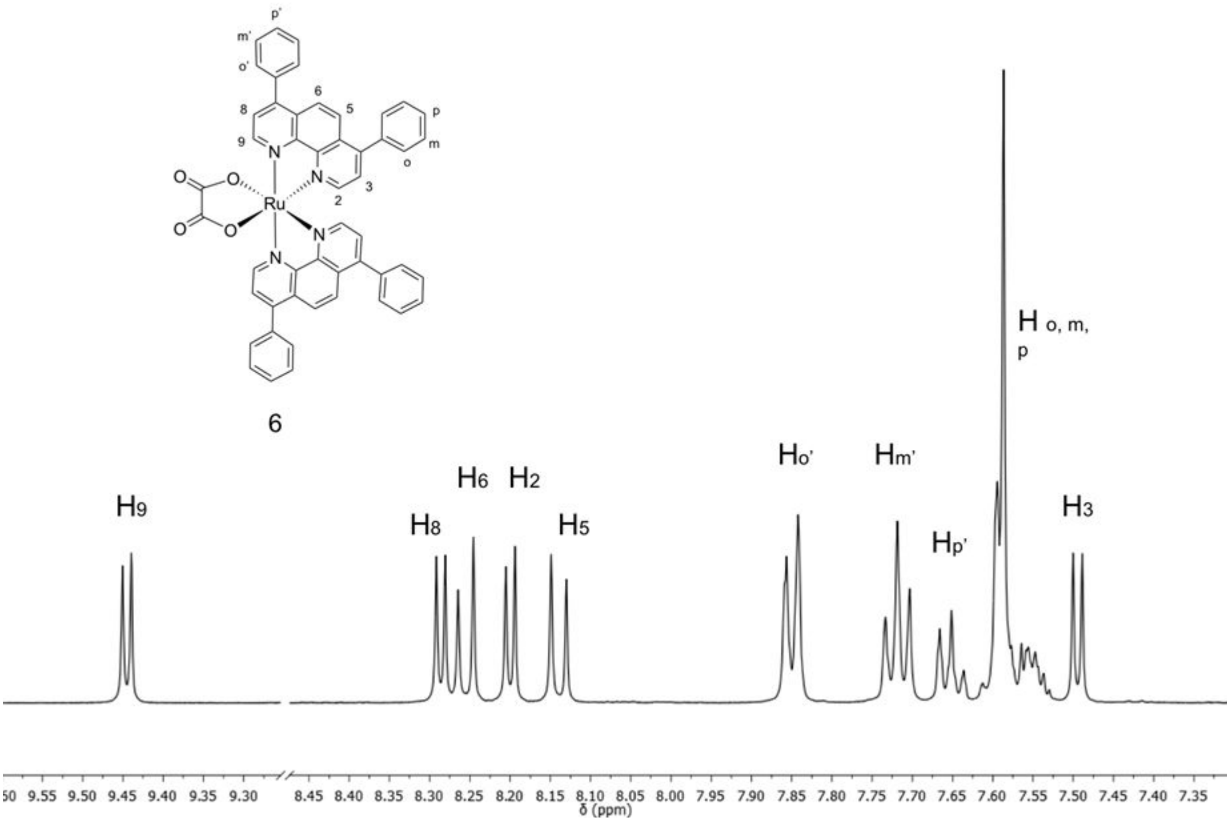

Figure S33. ^{1}H NMR spectrum (DMSO-d_6) of [Ru(dpphen)$_2$(η^2-ox)] (**13**) with labeling scheme.

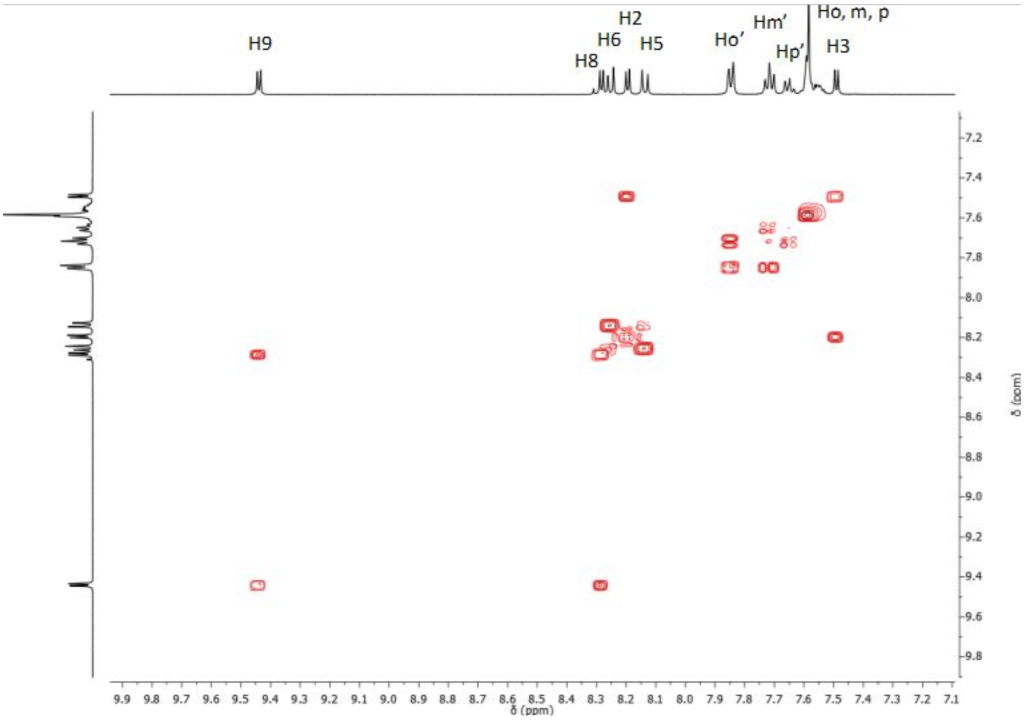

Figure S34. H-H COSY spectrum (DMSO-d_6) of [Ru(dpphen)$_2$(η^2-ox)] (**13**) see Figure S33 for labeling scheme.

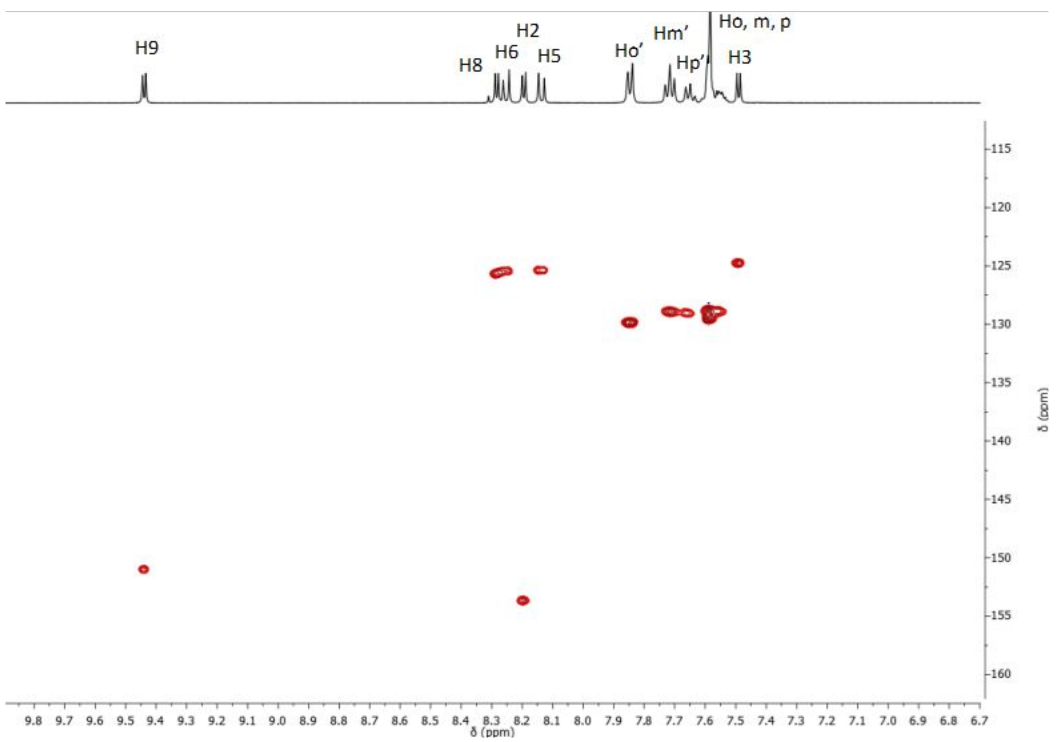

Figure S35. HSQC spectrum (DMSO-d_6) of [Ru(dpphen)$_2$(η^2-ox)] (**13**) see Figure S33 for the labeling scheme.

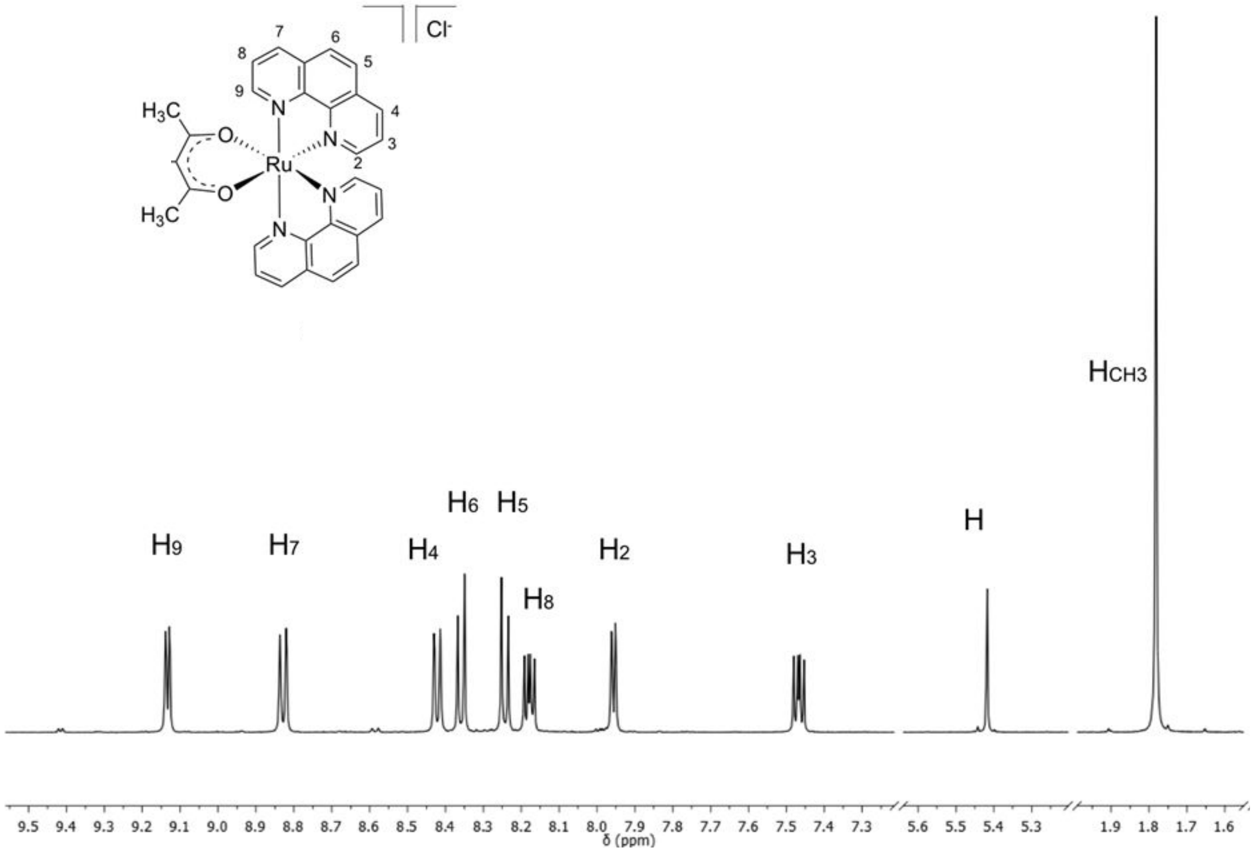

Figure S36. ^{1}H NMR spectrum (DMSO-d_6) of [Ru(phen)$_2$(η^2-acac)]Cl (**14**) with labeling scheme.

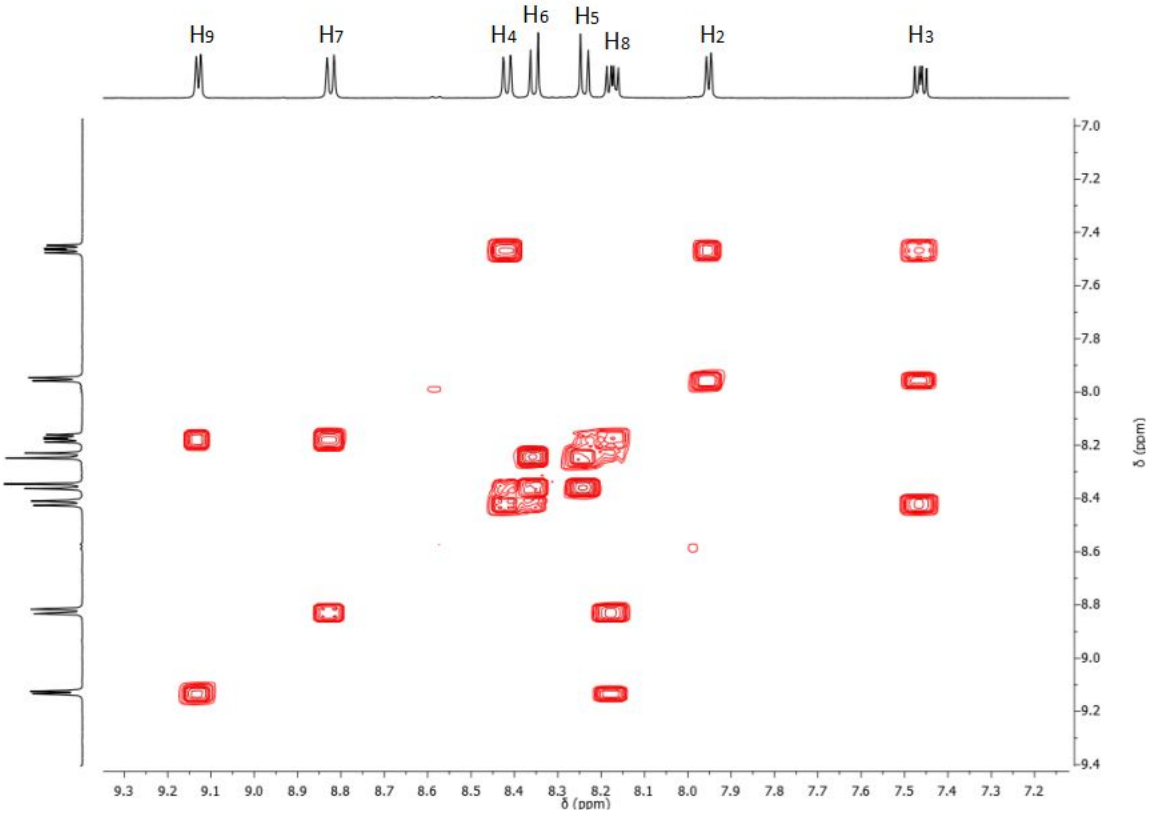

Figure S37. H-H COSY spectrum (DMSO-d_6) of [Ru(phen)$_2$(η^2-acac)]Cl (**14**); see Figure S36 for the labeling scheme.

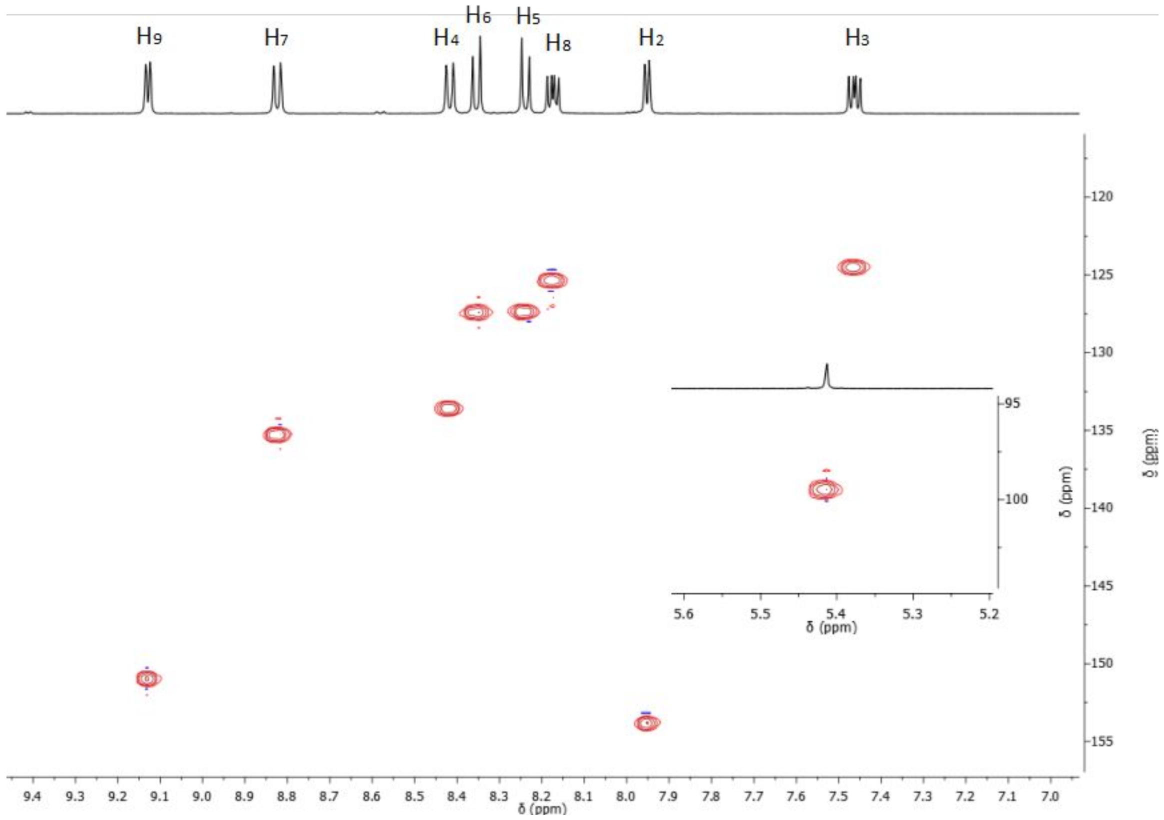

Figure S38. HSQC spectrum (DMSO-d_6) of [Ru(phen)$_2$(η^2-acac)]Cl (**14**); see Figure S36 for labeling scheme.

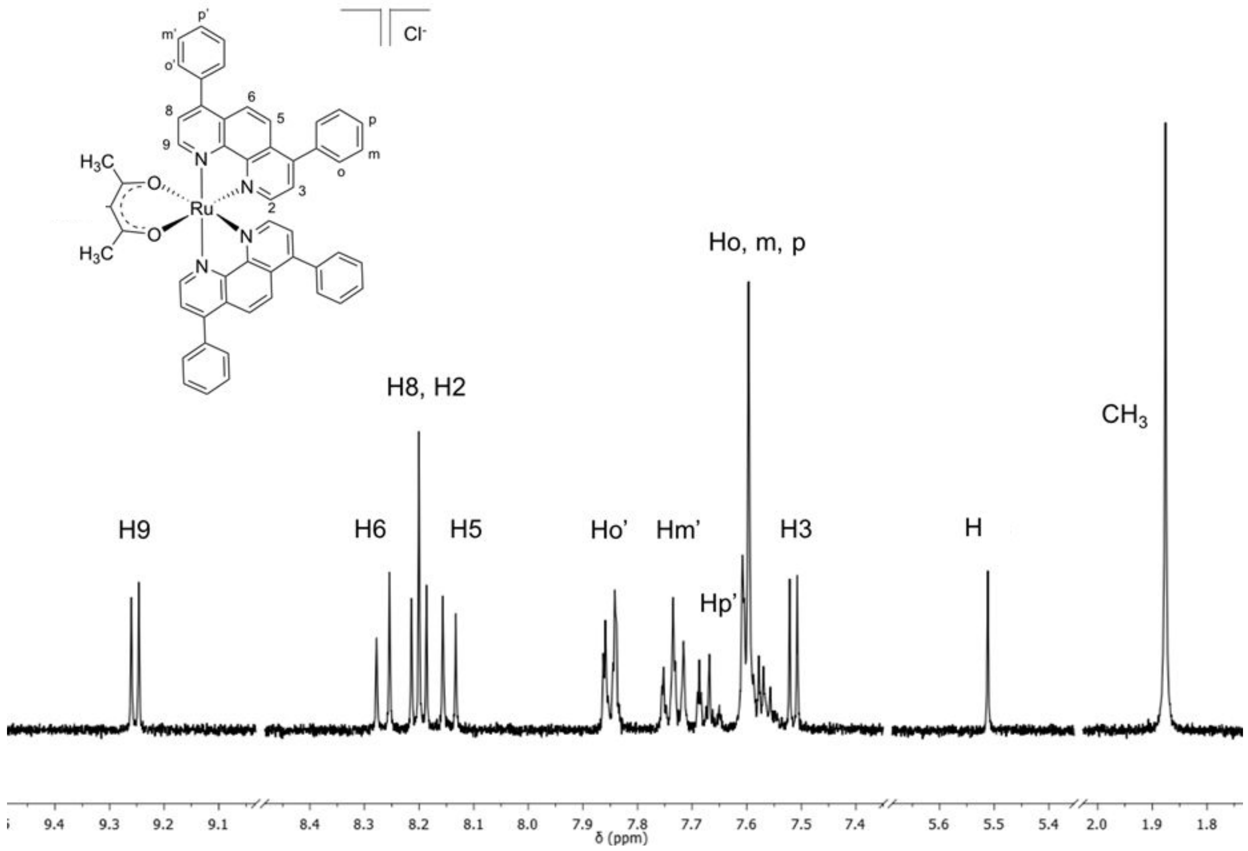

Figure S39. ^{1}H NMR spectrum (DMSO-d_6) of [Ru(dpphen)$_2$(η^2-acac)]Cl (**15**) with labeling scheme.

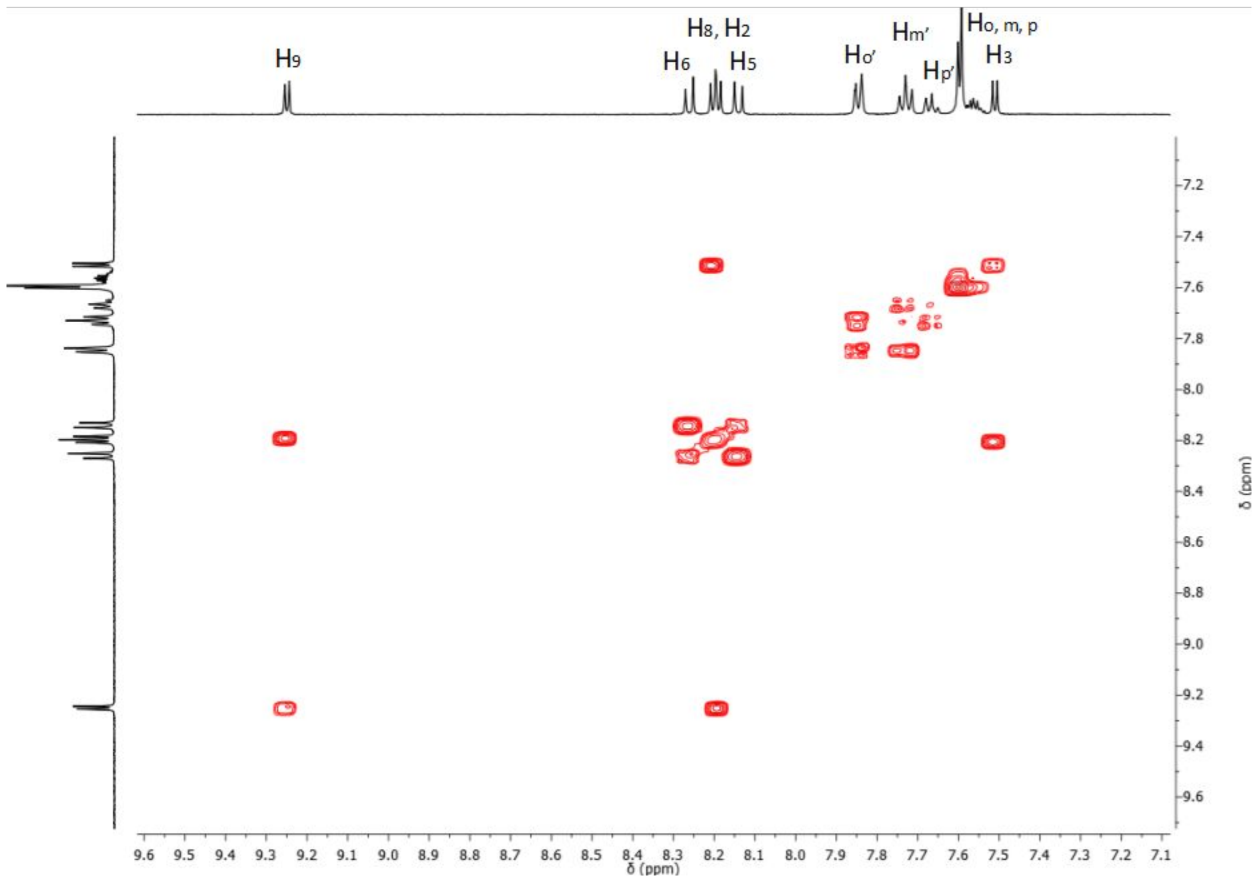

Figure S40. H-H COSY spectrum (DMSO-d_6) of [Ru(dpphen)$_2$(η^2-acac)]Cl (**15**); see Figure S39 for the labeling scheme.

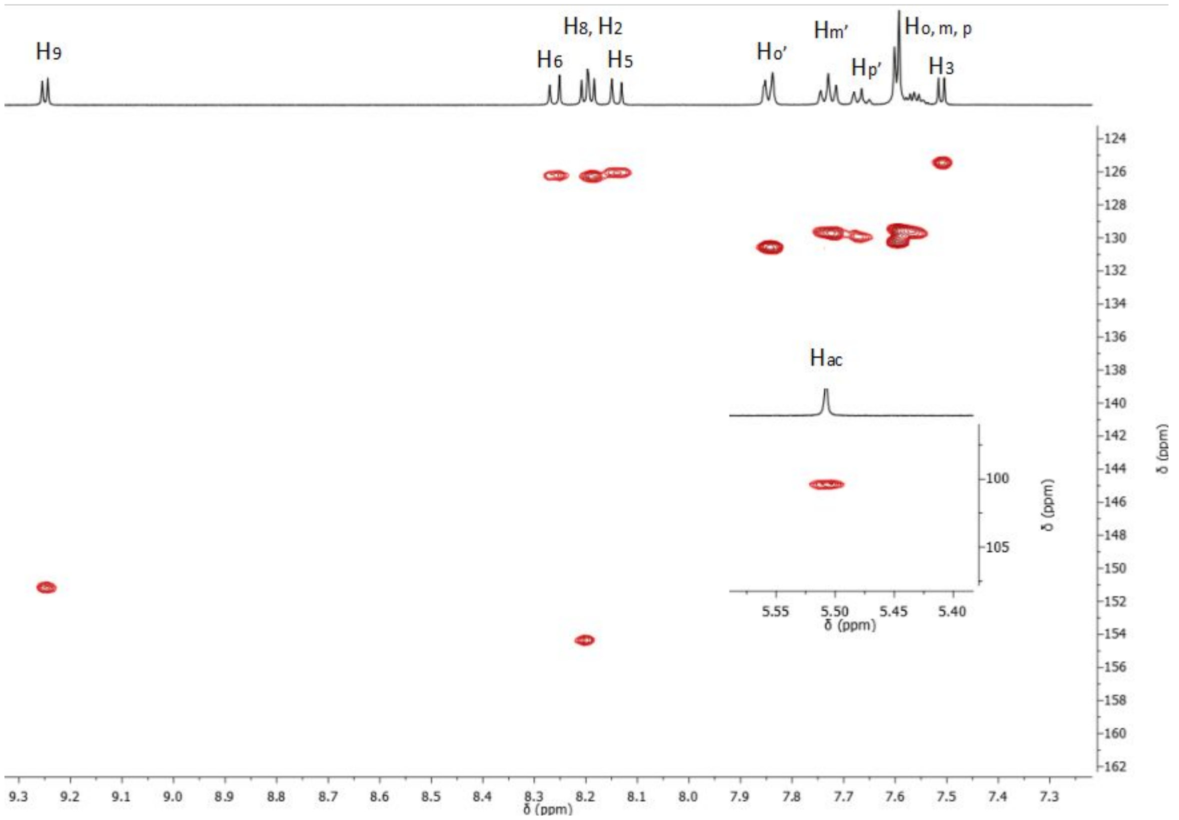

Figure S41. HSQC spectrum (DMSO-d_6) of [Ru(dpphen)$_2$(η^2-acac)]Cl (**15**); see Figure S39 for the labeling scheme.

Spectral characterization of the bis-heteroleptic complexes $[Ru(phen)_2(bpy)][PF_6]_2$ (16) and $[Ru(bpy)_2(phen)][PF_6]_2$ (17)

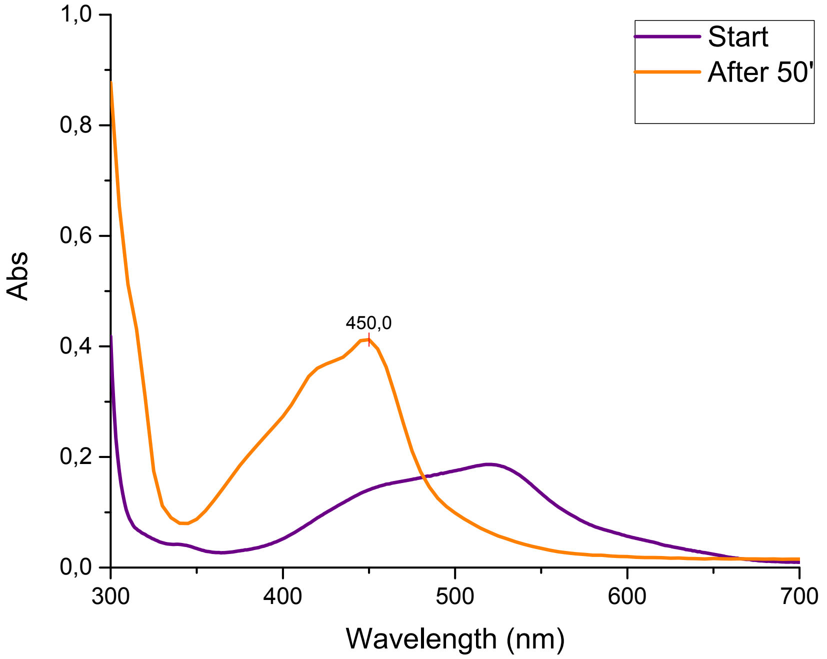

Figure S42. Spectral changes in the UV-vis spectrum during the reaction of $[Ru(phen)_2(\eta^2\text{-mal})]$ (**10**) (purple line) with one equiv. of bpy in refluxing EtOH + 10 equiv. of TFA to afford $[Ru(phen)_2(bpy)]^{2+}$ quantitatively in ca. 1h (orange line).

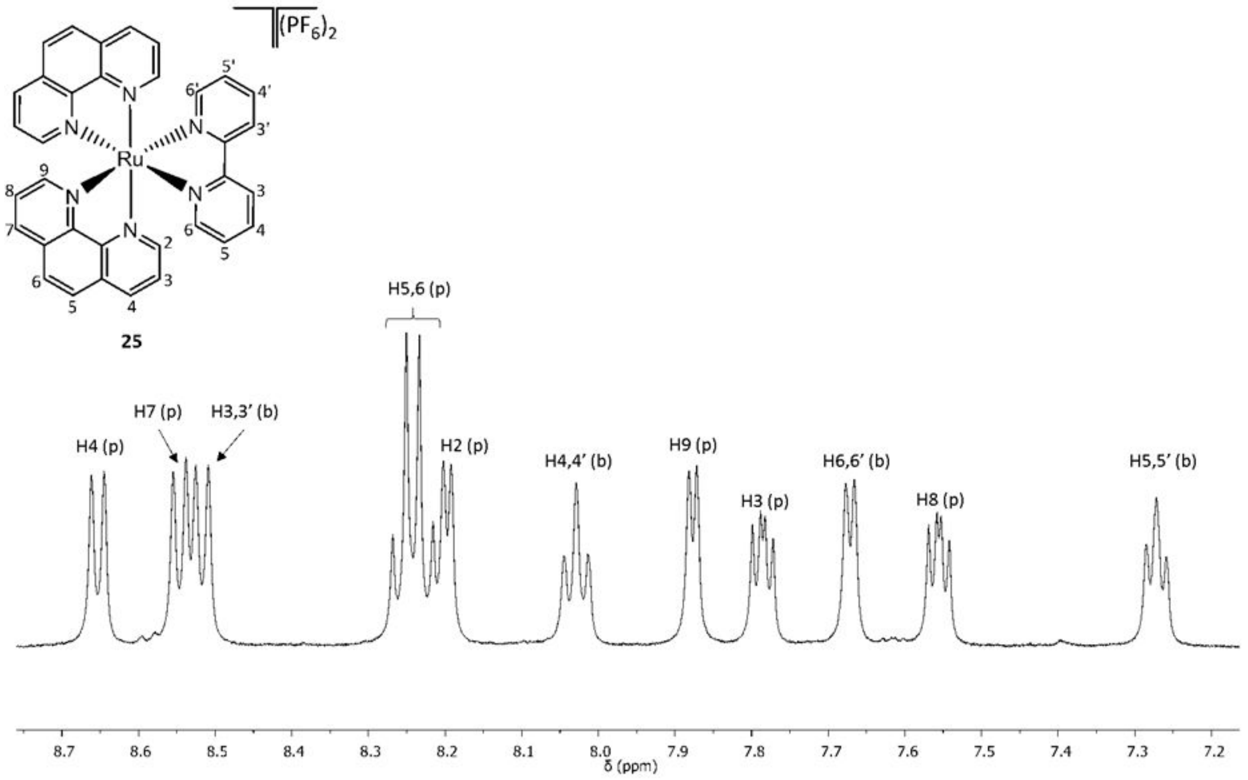

Figure S43. ^1H NMR spectrum (CD_3CN) of $[Ru(phen)_2(bpy)][PF_6]_2$ (**16**) with labelling scheme (p stands for phen, b for bpy).

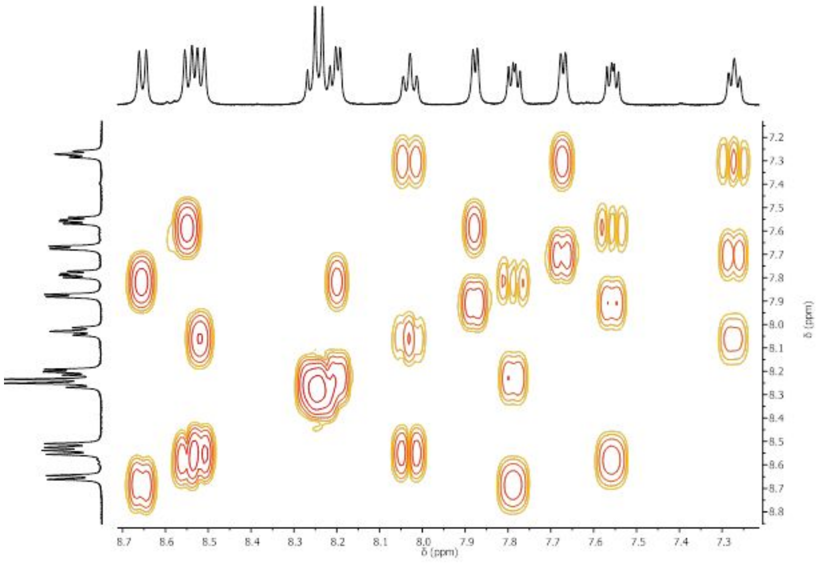

Figure S44. H-H COSY spectrum of (CD_3CN) of [Ru(phen)$_2$(bpy)][PF$_6$]$_2$ (**16**); see Figure S43 for labelling scheme.

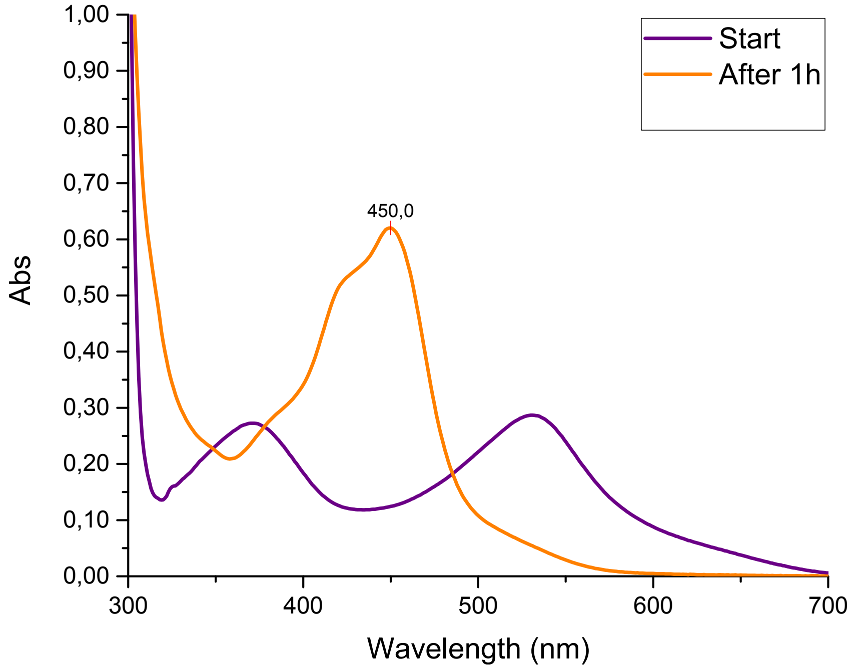

Figure S45. Spectral changes in the UV-vis spectrum during the reaction of [Ru(bpy)$_2$(η^2-mal)] (**9**) (purple line) with one equiv. of phen in refluxing EtOH + 10 equiv. of TFA to afford [Ru(bpy)$_2$(phen)]$^{2+}$ quantitatively in ca. 1h (orange line).

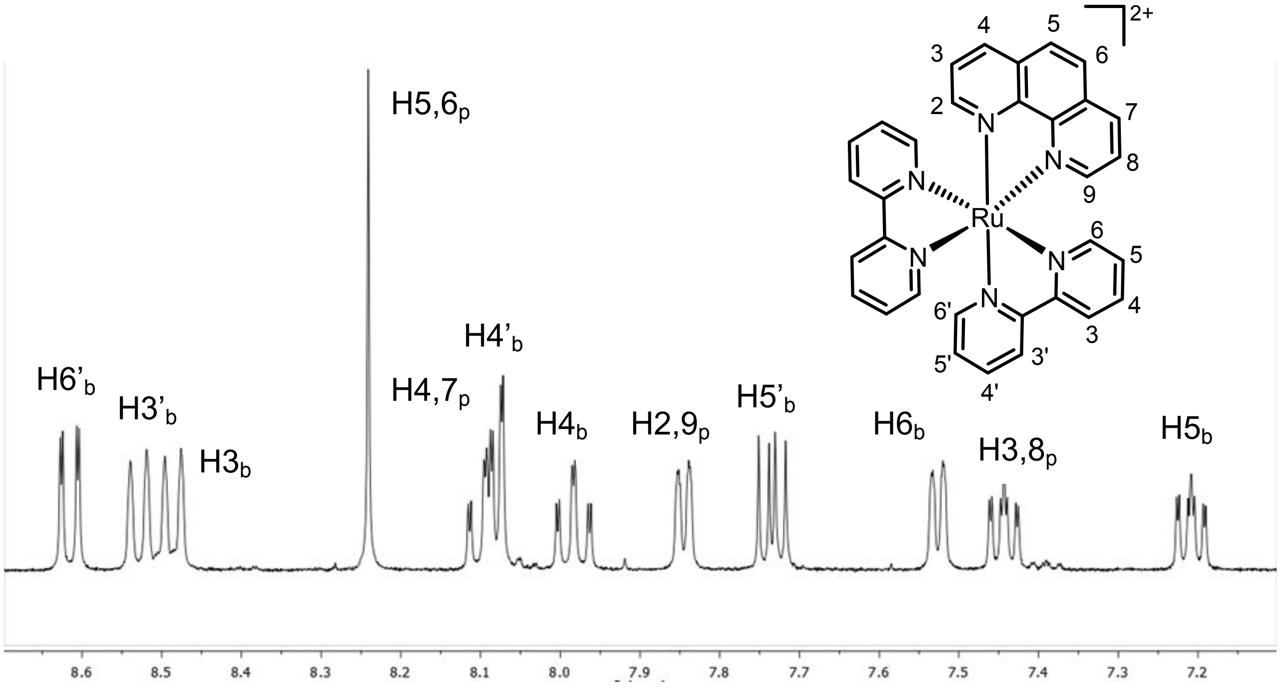

Figure S46. ^{1}H NMR spectrum (CD_3CN) of $[Ru(bpy)_2(phen)][PF_6]_2$ (**17**) with labelling scheme (p stands for phen, b for bpy).

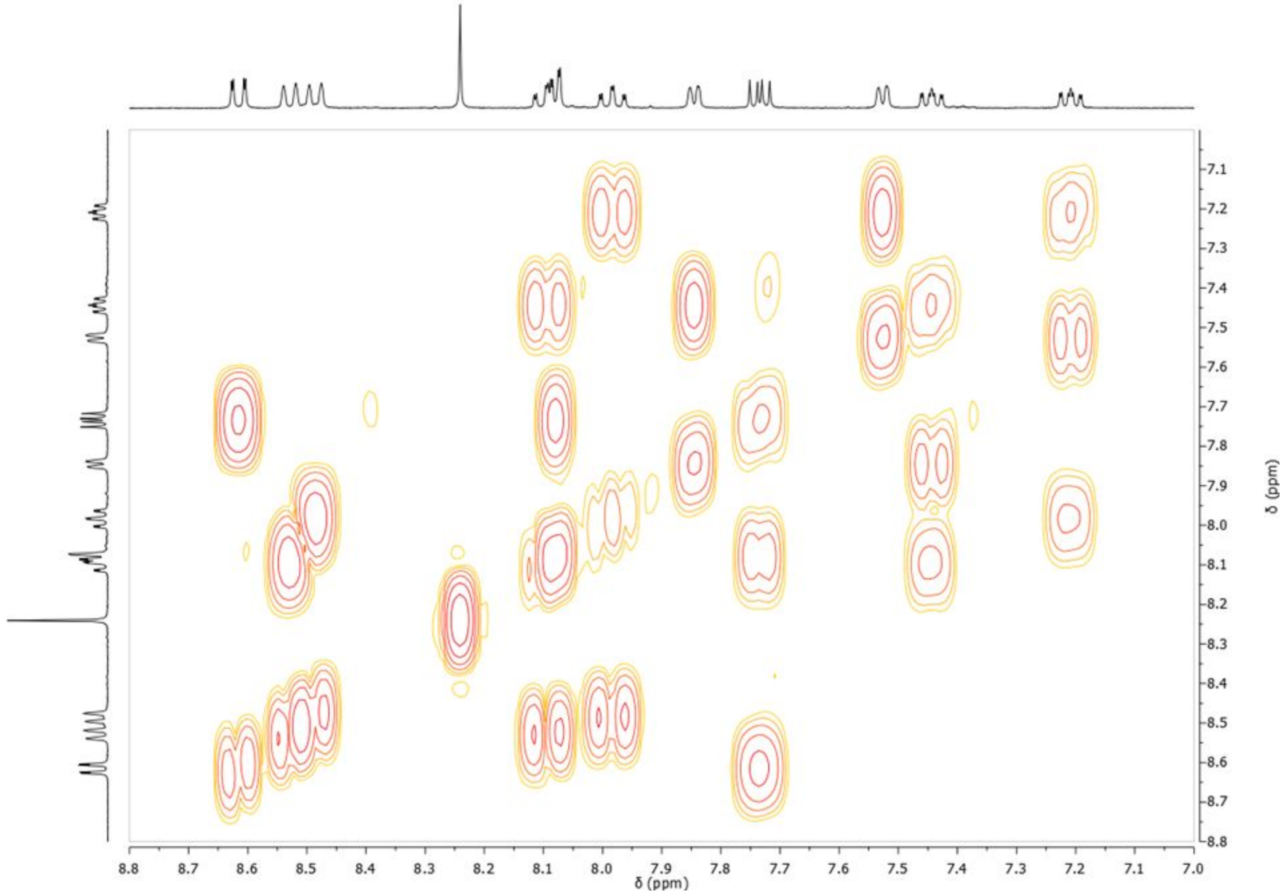

Figure S47. H-H COSY spectrum of (CD_3CN) of $[Ru(bpy)_2(phen)][PF_6]_2$ (**17**); see Figure S46 for labelling scheme.

Table S6. Crystallographic data and refinement details for compounds *fac*-[RuCl(dmso-S)$_3$(η^2-acac)] (**6**), *fac*-[Ru(dmso-O)(dmso-S)$_3$(η^2-acac)][PF_6] (**7**), and *cis,trans*-[$RuCl_2$(dmso-S)$_2$(phen)] (**c**).

	**6**	**7**	**c**
Empirical Formula	$C_{11}H_{25}ClO_5RuS_3$	$C_{13}H_{31}F_6O_6PRuS_4$	$C_{16}H_{20}Cl_2N_2O_2RuS_2$
Formula weight (Da)	470.01	657.66	508.43
Temperature (K)	100(2)	100(2)	100(2)
Wavelength (Å)	0.700	0.700	0.700
Crystal system	monoclinic	monoclinic	monoclinic
Space Group	P 21/n	P 21/c	P 21/c
a (Å)	8.356(2)	12.435(3)	16.164(7)
b (Å)	14.6850(7)	11.338(1)	19.153(9)
c (Å)	14.9920(6)	17.750(1)	13.189(3)
α (°)	90	90	90
β (°)	95.60(1)	91.79(2)	107.54(2)
γ (°)	90	90	90
V ($Å^3$)	1830.9(3)	2501.3(8)	3893(3)
Z	4	4	8
ρ ($g\cdot cm^{-3}$)	1.705	1.746	1.735
F(000)	960	1336	2048
μ (mm^{-1})	1.285	1.036	1.237
θ min, max (°)	1.916, 28.227	1.614, 29.998	1.301, 28.227
Resolution (Å)	0.74	0.70	0.74
Total refl. collctd	29469	89392	61622
Independent refl.	4676	7573	9911
Obs. Refl. [Fo>4σ(Fo)]	4673	7517	9898
I/σ(I) (all data)	63.33	45.80	62.71
I/σ(I) (max res)	49.75	34.68	53.01
Completeness (all data)	0.985	0.988	0.982
R_{merge} (all data)	2.0%	4.0%	2.4%
R_{merge} (max res)	2.0%	3.5%	2.7%
Multiplicity (all data)	6.2	11.5	6.1
Multiplicity (max res)	6.1	9.7	5.8
Data/restraint/parameters	4676/0/191	7573/0/291	9911/0/452
GooF	1.136	1.069	1.166
R[I>2.0σ(I)],[a] wR2 [I>2.0σ(I)][a]	0.0230, 0.0561	0.0306, 0.0816	0.0216, 0.0554
R (all data),[a] wR2 (all data)[a]	0.0230, 0.0561	0.0308, 0.0818	0.0217, 0.0554

Table S6 contd. Crystallographic data and refinement details for compounds [Ru(phen)$_2$(η^2-mal)]·5H_2O (**10**·5H_2O), [Ru(dpphen)$_2$(η^2-mal)]·3.75H_2O (**11**·3.75H_2O), and [Ru(phen)$_2$(η^2-ox)]·H_2O (**12**·H_2O).

	**10**·5H_2O	**11**·3.75H_2O	**12**·H_2O
Empirical Formula	$C_{27}H_{18}N_4O_4Ru·5H_2O$	$C_{51}H_{34}N_4O_4Ru·3.75H_2O$	$C_{26}H_{16}N_4O_4Ru·H_2O$
Formula weight (Da)	653.60	935.45	567.51
Temperature (K)	100(2)	100(2)	100(2)
Wavelength (Å)	0.700	0.700	0.700
Crystal system	triclinic	triclinic	triclinic
Space Group	P –1	P –1	P –1
a (Å)	10.322(3)	12.765(5)	9.5710(7)
b (Å)	10.569(2)	13.403(2)	11.3260(5)
c (Å)	13.064(3)	26.445(3)	12.1490(5)
α (°)	84.832(6)	102.828(8)	83.891(6)
β (°)	76.176(8)	90.20(2)	74.856(4)
γ (°)	72.432(5)	108.05(1)	72.743(6)
V (Å^3)	1319.1(5)	4182.0(2)	1213.4(1)
Z	1	4	2
ρ (g·cm^{-3})	1.646	1.486	1.553
F(000)	668	1926	572
μ (mm^{-1})	0.624	0.416	0.655
θ min, max (°)	1.581, 28.227	0.780,24.014	2.484,28.224
Resolution (Å)	0.74	0.74	0.74
Total refl. collctd	6720	127131	58712
Independent refl.	6720	13220	6156
Obs. Refl. [Fo>4σ(Fo)]	6575	11730	6087
I/σ(I) (all data)	46.09	30.10	100.64
I/σ(I) (max res)	37.72	13.39	69.35
Completeness (all data)	0.989	0.960	0.977
R_{merge} (all data)	3.2%	4.5%	1.7%
R_{merge} (max res)	4.1%	14.7%	2.6%
Multiplicity (all data)	6.0	9.3	9.5
Multiplicity (max res)	5.6	8.8	9.1
Data/restraint/parameters	6720/17/327	13220/66/1081	6156/15/316
GooF	1.072	1.047	1.095
R[I>2.0σ(I)],[a] wR2 [I>2.0σ(I)][a]	0.0459, 0.1531	0.0741, 0.1989	0.0366, 0.0936
R (all data),[a] wR2 (all data)[a]	0.0464, 0.1536	0.0811, 0.2070	0.0368, 0.0938

Table S7. Selected coordination distances (Å) and angles (°) for *fac*-[RuCl(dmso-S)$_3$(η^2-acac)] (**6**).

Selected distances (Å)

Ru1–Cl1	2.4326(4)
Ru1–O1	2.075(1)
Ru1–O2	2.064(1)
Ru1–S1	2.2516(5)
Ru1–S2	2.2622(5)
Ru1–S3	2.2650(4)

Selected angles (°)

O1–Ru1–Cl1	85.58(4)
O1–Ru1–S1	177.39(4)
O1–Ru1–S2	87.41(4)
O1–Ru1–S3	87.89(4)
O2–Ru1–Cl1	86.13(4)
O2–Ru1–O1	91.38(5)
O2–Ru1–S1	86.02(4)
O2–Ru1–S2	174.40(4)
O2–Ru1–S3	90.79(4)
S1–Ru1–Cl1	94.49(1)
S1–Ru1–S2	95.21(2)
S1–Ru1–S3	91.89(2)
S2–Ru1–Cl1	88.33(2)
S2–Ru1–S3	94.62(2)
S3–Ru1–Cl1	172.70(2)

Table S8. Selected coordination distances (Å) and angles (°) for *fac*-[Ru(dmso-O)(dmso-S)$_3$(η^2-acac)][PF_6] (**7**).

Selected distances (Å)

Ru1–O11	2.071(1)
Ru1–O12	2.066(1)
Ru1–O21	2.133(1)
Ru1–S31	2.2490(6)
Ru1–S41	2.2493(6)
Ru1–S51	2.2667(5)

Selected angles (°)

O11–Ru1–O21	86.50(5)
O11–Ru1–S31	90.73(4)
O11–Ru1–S41	174.52(4)
O11–Ru1–S51	87.03(4)
O12–Ru1–O11	90.61(5)
O12–Ru1–O21	82.15(5)
O12–Ru1–S31	90.52(4)
O12–Ru1–S41	90.68(4)
O12–Ru1–S51	174.84(4)
O21–Ru1–S31	172.13(4)
O21–Ru1–S41	88.40(4)
O21–Ru1–S51	93.11(4)
S31–Ru1–S41	94.58(2)
S31–Ru1–S51	94.10(2)
S41–Ru1–S51	91.25(2)

Table S9. Selected coordination distances (Å) and angles (°) for *cis,trans*-[$RuCl_2$(dmso-S)$_2$(phen)] (**c**).

Selected distances (Å)

Ru1–Cl11	2.4292(9)	Ru2–Cl21	2.4360(8)
Ru1–Cl12	2.4301(8)	Ru2–Cl22	2.4286(8)
Ru1–N11	2.071(1)	Ru2–N21	2.078(1)
Ru1–N12	2.062(1)	Ru2–N22	2.05591)
Ru1–S11	2.3170(8)	Ru2–S21	2.3082(7)
Ru1–S12	2.3027(8)	Ru2–S22	2.3177(7)

Selected angles (°)

Cl11–Ru1–Cl12	92.20(3)	Cl21–Ru2–Cl22	91.86(4)
N11–Ru1–Cl11	94.07(4)	N21–Ru2–Cl21	172.89(3)
N11–Ru1–Cl12	173.67(3)	N21–Ru2–Cl22	95.22(5)
N11–Ru1–S11	89.81(4)	N21–Ru2–S21	92.07(4)
N11–Ru1–S12	91.82(4)	N21–Ru2–S22	89.23(4)
N12–Ru1–Cl11	173.94(3)	N22–Ru2–Cl21	92.86(5)
N12–Ru1–Cl12	93.56(4)	N22–Ru2–Cl22	175.24(3)
N12–Ru1–N11	80.14(5)	N22–Ru2–N21	80.06(6)
N12–Ru1–S11	88.31(3)	N22–Ru2–S21	90.27(4)
N12–Ru1–S12	92.63(3)	N22–Ru2–S22	91.99(4)
S11–Ru1–Cl11	89.90(2)	S21–Ru2–Cl21	88.70(2)
S11–Ru1–Cl12	89.30(3)	S21–Ru2–Cl22	89.31(2)
S12–Ru1–Cl11	89.32(2)	S22–Ru2–Cl21	90.27(2)
S12–Ru1–Cl12	89.15(3)	S22–Ru2–Cl22	88.51(2)
S12–Ru1–S11	178.24(1)	S22–Ru2–S21	177.56(1)

Table S10. Selected coordination distances (Å) and angles (°) for [Ru(phen)$_2$(η^2-mal)]·5H_2O (**10**·5H_2O).

Selected distances (Å)

Ru1–N11	2.034(3)
Ru1–N12	2.049(3)
Ru1–N21	2.063(3)
Ru1–N22	2.025(3)
Ru1–O31	2.089(3)
Ru1–O32	2.092(3)

Selected angles (°)

N11–Ru1–N12	80.9(1)
N11–Ru1–N21	95.5(1)
N11–Ru1–O31	88.5(1)
N11–Ru1–O32	171.4(1)
N12–Ru1–N21	174.9(1)
N12–Ru1–O31	86.5(1)
N12–Ru1–O32	90.5(1)
N21–Ru1–O31	97.0(1)
N21–Ru1–O32	93.2(1)
N22–Ru1–N11	95.1(1)
N22–Ru1–N12	96.2(1)
N22–Ru1–N21	80.4(1)
N22–Ru1–O31	175.7(1)
N22–Ru1–O32	86.2(1)
O31–Ru1–O32	90.6(1)

Table S11. Selected coordination distances (Å) and angles (°) for [Ru(dpphen)$_2$(η^2-mal)]·3.75H_2O (**11**·3.75H_2O).

Selected distances (Å)

Ru1–N21	2.046(5)	Ru2–N51	2.035(5)
Ru1–N22	2.034(5)	Ru2–N52	2.048(5)
Ru1–N31	2.047(5)	Ru2–N61	2.057(5)
Ru1–N32	2.023(5)	Ru2–N62	2.019(5)
Ru1–O11	2.100(4)	Ru2–O41	2.092(4)
Ru1–O12	2.097(4)	Ru2–O42	2.100(4)

Selected angles (°)

N21–Ru1–N31	174.5(2)	N51–Ru2–N52	79.7(2)
N21–Ru1–O11	91.6(2)	N51–Ru2–N61	97.3(2)
N21–Ru1–O12	86.2(2)	N51–Ru2–O41	169.9(2)
N22–Ru1–N21	80.5(2)	N51–Ru2–O42	86.3(2)
N22–Ru1–N31	95.0(2)	N52–Ru2–N61	175.1(2)
N22–Ru1–O11	171.8(2)	N52–Ru2–O41	91.1(2)
N22–Ru1–O12	88.1(2)	N52–Ru2–O42	86.7(2)
N31–Ru1–O11	93.1(2)	N61–Ru2–O41	92.1(2)
N31–Ru1–O12	96.7(2)	N61–Ru2–O42	97.0(2)
N32–Ru1–N21	97.3(2)	N62–Ru2–N51	100.0(2)
N32–Ru1–N22	96.0(2)	N62–Ru2–N52	97.1(2)
N32–Ru1–N31	80.2(2)	N62–Ru2–N61	79.6(2)
N32–Ru1–O11	87.1(2)	N62–Ru2–O41	85.2(2)
N32–Ru1–O12	175.0(2)	N62–Ru2–O42	173.1(2)
O12–Ru1–O11	89.2(2)	O41–Ru2–O42	89.0(2)

Table S12. Selected coordination distances (Å) and angles (°) for [Ru(phen)$_2$(η^2-ox)]·H_2O (**12**·H_2O).

Selected distances (Å)

Ru1–N21	2.057(2)
Ru1–N22	2.026(2)
Ru1–N31	2.040(2)
Ru1–N32	2.032(2)
Ru1–O11	2.081(2)
Ru1–O12	2.0822(2)

Selected angles (°)

N21–Ru1–O11	93.13(7)
N21–Ru1–O12	85.37(7)
N22–Ru1–N21	80.54(8)
N22–Ru1–N31	95.05(8)
N22–Ru1–N32	95.37(8)
N22–Ru1–O11	171.41(8)
N22–Ru1–O12	93.82(8)
N31–Ru1–N21	175.37(7)
N31–Ru1–O11	91.11(7)
N31–Ru1–O12	93.54(7)
N32–Ru1–N21	101.26(8)
N32–Ru1–N31	80.48(8)
N32–Ru1–O11	91.55(7)
N32–Ru1–O12	169.44(7)
O11–Ru1–O12	79.80(7)

References

S1) Zakeeruddin, S. M.; Nazeeruddin, Md. K.; Humphry-Baker, R.; Grätzel, M. Stepwise Assembly of Tris-Heteroleptic Polypyridyl Complexes of Ruthenium(II) Inorg. Chem., 1998, 37, 5251-5259.

S2) Spiccia, L.; Deacon, G. B.; Kepert, C. M. Synthetic routes to homoleptic and heteroleptic ruthenium(II) complexes incorporating bidentate imine ligands Coord. Chem. Rev. 2004, 248, 1329-1341.

S3) Toyama, M.; Inoue, K.; Iwamatsu, S.; Nagao, N. Syntheses and Crystal Structures of Mono(2,2′-bipyridine)dichlorobis-(dimethyl sulfoxide-*S*)ruthenium(II) Complexes, [$RuCl_2$(bpy)(dmso-*S*)$_2$] Bull. Chem. Soc. Jpn. 2006, 79, 1525-1234.

S4) Burke, C.; Keyes, T. E. An efficient route to asymmetrically diconjugated tris(heteroleptic) complexes of Ru(II) *RSC Adv.*, **2016**, *6*, 40869-40877.
